# Assessing Vancomycin‐Formyl Peptide Conjugate Binding to the Surface of Resistant *Staphylococcus aureus* via Flow Cytometry

**DOI:** 10.1002/cbic.70495

**Published:** 2026-08-05

**Authors:** Winfrey P. Y. Hoo, Jemma Gullick, Ryan F. Leung, Alex J. Fulcher, Rachel W. Cass, Julien Tailhades, Anton Y. Peleg, Jennifer A. E. Payne, Meredith O’Keeffe, Max J. Cryle

**Affiliations:** ^1^ Infection and Immunity Program Department of Biochemistry and Molecular Biology Monash Biomedicine Discovery Institute Monash University Clayton Victoria Australia; ^2^ ARC Centre of Excellence for Innovations in Peptide and Protein Science Clayton Victoria Australia; ^3^ Monash Micro Imaging Monash University Clayton Victoria Australia; ^4^ Infection Program Department of Microbiology Monash Biomedicine Discovery Institute Monash University Clayton Victoria Australia; ^5^ Centre to Impact AMR Monash University Clayton Victoria Australia; ^6^ Department of Infectious Diseases Alfred Health and School of Translational Medicine Monash University Melbourne Victoria Australia; ^7^ Curiosity Factory Ltd. Surry Hills New South Wales Australia; ^8^ Immunity Program Department of Biochemistry and Molecular Biology Monash Biomedicine Discovery Institute Monash University Clayton Victoria Australia

**Keywords:** flow cytometry, fluorescent peptide, formyl peptide, vancomycin

## Abstract

*Staphylococcus aureus* strains have emerged with resistance mechanisms that reduce the efficacy of last resort antibiotics and evade the immune system. One strategy to combat antimicrobial resistance is to modulate host immunity to eliminate infections more effectively. This has led to the development of immunotherapeutics consisting of vancomycin conjugated to formyl peptides (fPeps), with vancomycin targeting the cell wall and the fPeps engaging host innate immunity. Here, we used flow cytometry to quantify the binding of vancomycin=fPep conjugates to *S. aureus* clinical isolates. This revealed reduced binding of vancomycin=fPeps compared to vancomycin alone and quantified the interaction between the conjugates and the bacterial cell surface, which is important to quantify to then control the chemotactic gradient established by the fPep cargo. The direct antimicrobial activity of these conjugates was also reduced when compared to vancomycin, reflecting the reduced binding of these conjugates to *S. aureus*. This flow cytometry method allows quantification of vancomycin=fPep binding to bacteria and will assist in future studies to understand how attached fPeps and other immune signalling cargoes can stimulate innate immune cell activation leading to bacterial phagocytosis.

## Graphical abstract

A flow cytometry technique was developed to measure the binding of vancomycin‐formyl peptide conjugates to the surface of resistant *Staphylococcus aureus*. Binding results indicate that the labelling site of the vancomycin targeting element has a major effect on binding to the bacterial cell wall, which correlates with reduced antibiotic activity. Understanding the interaction of the vancomycin targeting element with resistant bacteria is important in quantifying the delivery of the conjugated formyl peptide that is used to recruit host innate immune cells, which in turn is important for developing this approach to tackle antimicrobial resistance.

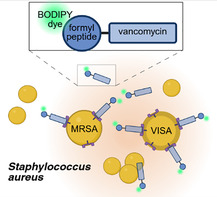

## 1 Introduction

The emergence of antimicrobial resistance (AMR) has given rise to so‐called superbugs, which are exemplified by the ESKAPE panel (*Enterococcus faecium*, *Staphylococcus aureus*, *Klebsiella pneumoniae*, *Acinetobacter baumannii*, *Pseudomonas aeruginosa* and *Enterobacter* spp.) and that are resistant to multiple antimicrobials, leading to increased hospitalisations and mortality [1, 2]. Among the more widely studied of such bacteria, methicillin‐resistant *S. aureus* (MRSA) has seen the highest global increase with 550,000 deaths associated with AMR and 130,000 deaths attributable to AMR in 2021 alone [3]. MRSA is responsible for most of the healthcare‐ and community‐associated infections globally [4], with these infections often treated with last‐line antibiotics such as vancomycin and daptomycin [5, 6]. Increased use of these last resort antibiotics has led to the emergence and spread of strains with mechanisms that reduce their effectiveness [7, 8], which often occurs along with a concomitant reduction in the recognition of these bacteria by the immune system [9, 11]. Here, it is important to note that the composition of the bacterial cell wall and membrane can fluctuate between isolates even within the same strain [12, 13]. In Gram‐positive strains, the peptidoglycan layer can be modified, resulting in a thicker cell wall (vancomycin intermediary resistance [14, 15]), altered surface charge (daptomycin resistance [16]) or target modification such as changes to the structure of Lipid II (vancomycin resistance [17, 18]). This often leads to higher concentrations of antibiotic being required for efficacy, causing toxicity for the patient [12, 13].

**FIGURE 1:**
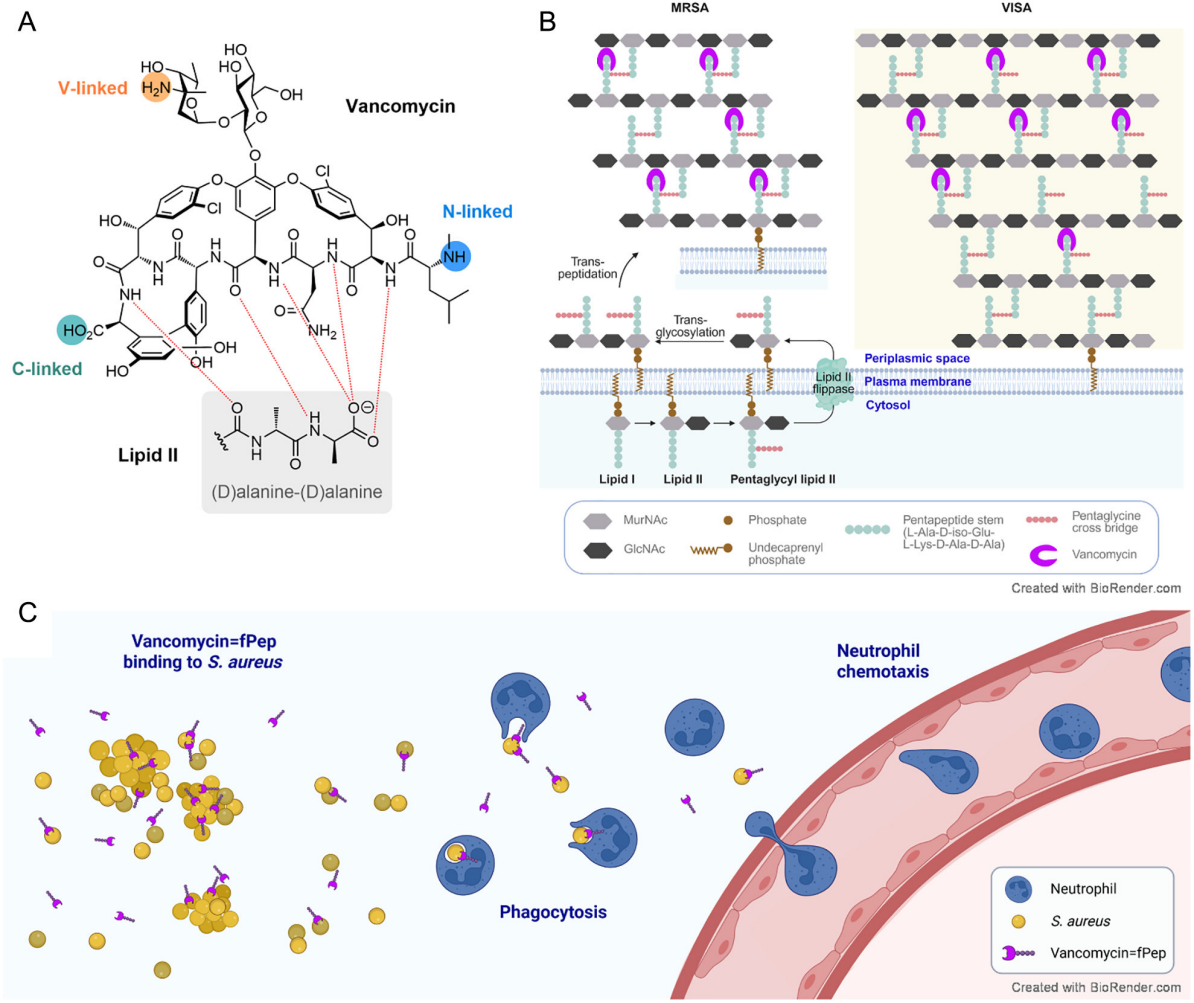
Mechanism of vancomycin in an immunotherapeutic approach. (A) Structure of vancomycin and its binding mechanism to Lipid II precursor. Vancomycin binding is facilitated by five hydrogen bonds (red lines) to the (D)Ala‐(D)Ala terminus of the Lipid II precursor in the cell wall of vancomycin‐susceptible bacteria. Meanwhile, the sites for conjugation of fPep to vancomycin can be made through three sites: the vancosamine primary amine (V‐linked, orange); methylated amine (N‐linked, blue); or the carboxyl group (C‐linked, teal). (B) Vancomycin binding to the cell wall of MRSA and VISA. Diagram of the peptidoglycan biosynthesis of *S. aureus*, where mature peptidoglycan is formed by transglycosylation and transpeptidation of Lipid II precursors. The thickened peptidoglycan layer of VISA (right) differs from MRSA, which increases the cell wall interaction with vancomycin but reduces its activity by preventing vancomycin from reaching to the septum where it can inhibit cell wall synthesis [17, 20, 24]. MurNAc: N‐acetylmuramic acid; GlcNAc: N‐acetylglucosamine. (C) Modulation of host immunity to target *S. aureus*. An immunotherapeutic approach using antibiotic‐chemoattractants generated from the conjugation of fPeps to vancomycin. While vancomycin is used as a cell wall targeting element, the attached fPeps are concomitantly delivered to the surface of *S. aureus*, which attracts innate immune cells (such as neutrophils) as a first line of defence in response to the infection.

Vancomycin, a member of the glycopeptide antibiotic (GPA) family, serves as an example of an antibiotic in clinical use that is facing a rise in bacterial resistance, exemplifying the challenges that exist in the application of last resort antibiotics to the treatment of MRSA [19]. Vancomycin exerts its mechanism by binding to the (D)Ala‐(D)Ala terminus of the Lipid II precursor of the bacterial peptidoglycan cell wall (Figure 1A) [17, 25, 26]. This non‐covalent interaction is mediated by five hydrogen bond interactions between the peptide backbone of the antibiotic and the target, which prevents the actions of transpeptidase enzymes (and to some extent transglycosylase enzymes) that construct the bacterial cell wall, leaving the bacteria prone to osmotic pressure and lysis [27, 28]. The binding mechanism of vancomycin can be analysed in detail using isolated Lipid II components or whole bacteria. Visualisation of molecules that bind to Lipid II is an increasingly prevalent approach to understand the interaction and binding mechanism of antibiotics to bacterial cells [29]. While past studies have improved our understanding of molecular interactions between Lipid II and antibiotics such as β‐lactams [30] and lipoglycopeptides [18], the isolation or chemical syntheses of Lipid II targets remains challenging due to variations in the peptidoglycan precursors (including the terminal peptide moiety of Lipid II, variations of the sugar moieties and the pentaglycine interpeptide bridge crucial for peptidoglycan crosslinking) as part of the evolution of bacterial resistance to antimicrobials [31]. Studies by Yim et al. [32] reported the use of fluorescent labelling of vancomycin to observe glycopeptide antibiotic (GPA) binding to the septum, while Benincasa et al. [33] have used fluorescence quenching to detect interaction between antimicrobial peptides and Gram‐negative bacteria. The accurate quantification of the level of binding of such antimicrobials is therefore a key step in understanding the effects of bacterial resistance.

A systematic review and meta‐analysis performed by Shariati et al. [34] reported a global prevalence of 1.7% for vancomycin‐intermediate *S. aureus* (VISA) isolates among human clinical samples. VISA resistance is due to the thickened cell wall of these strains coupled to the presence of vancomycin binding to decoy sites (incompletely crosslinked peptidoglycan monomers terminating with (D)Ala‐(D)Ala) that reduces the ability of the antibiotic to reach and block cell wall synthesis occurring at the septum close to the cell membrane (Figure 1B) [8, 35, 37]. These decoy binding sites lead to increased vancomycin binding to the cell wall but reduced direct antimicrobial activity, hence requiring an increased concentration of vancomycin for direct killing [38].

Our research efforts have pursued an immunotherapeutic approach that modulates host innate immunity to target *S. aureus* rather than the traditional route of killing bacteria by direct antimicrobial action [39]. This immunotherapeutic approach uses antibiotic‐chemoattractants that consist of vancomycin conjugated to formyl peptides (fPeps), with vancomycin used as a cell wall targeting element to deliver the fPeps to *S. aureus* (Figure 1C). The vancomycin=fPep conjugates bound to the bacteria surface then establish a chemoattractive gradient that serves as a signal for the recruitment of host phagocytes to target and clear the bacterial infection. Previous studies by Payne et al. [39] indicate that vancomycin=fPep conjugates can enhance neutrophil recruitment to MRSA, where the level of phagocytosis triggered upon recruitment was found to be dependent on the fPep sequence. We had previously observed that the C‐terminal conjugation of fPeps to vancomycin showed the highest antimicrobial activity and enhanced neutrophil recruitment to MRSA out of all linkage points tested [39]. Now, we wished to quantify the binding of these immunotherapeutics to surface of *S. aureus* and to examine the potential correlation of binding with antimicrobial activity. This therefore requires the fluorescent labelling of these immunotherapeutics and subsequent assessment of their binding to bacterial strains using flow cytometry [40].

## 2 Results

### 2.1 Binding of Vancomycin=fMLFK Conjugates to the Surface of MRSA and VISA Clinical Isolates

**SCHEME 1:**
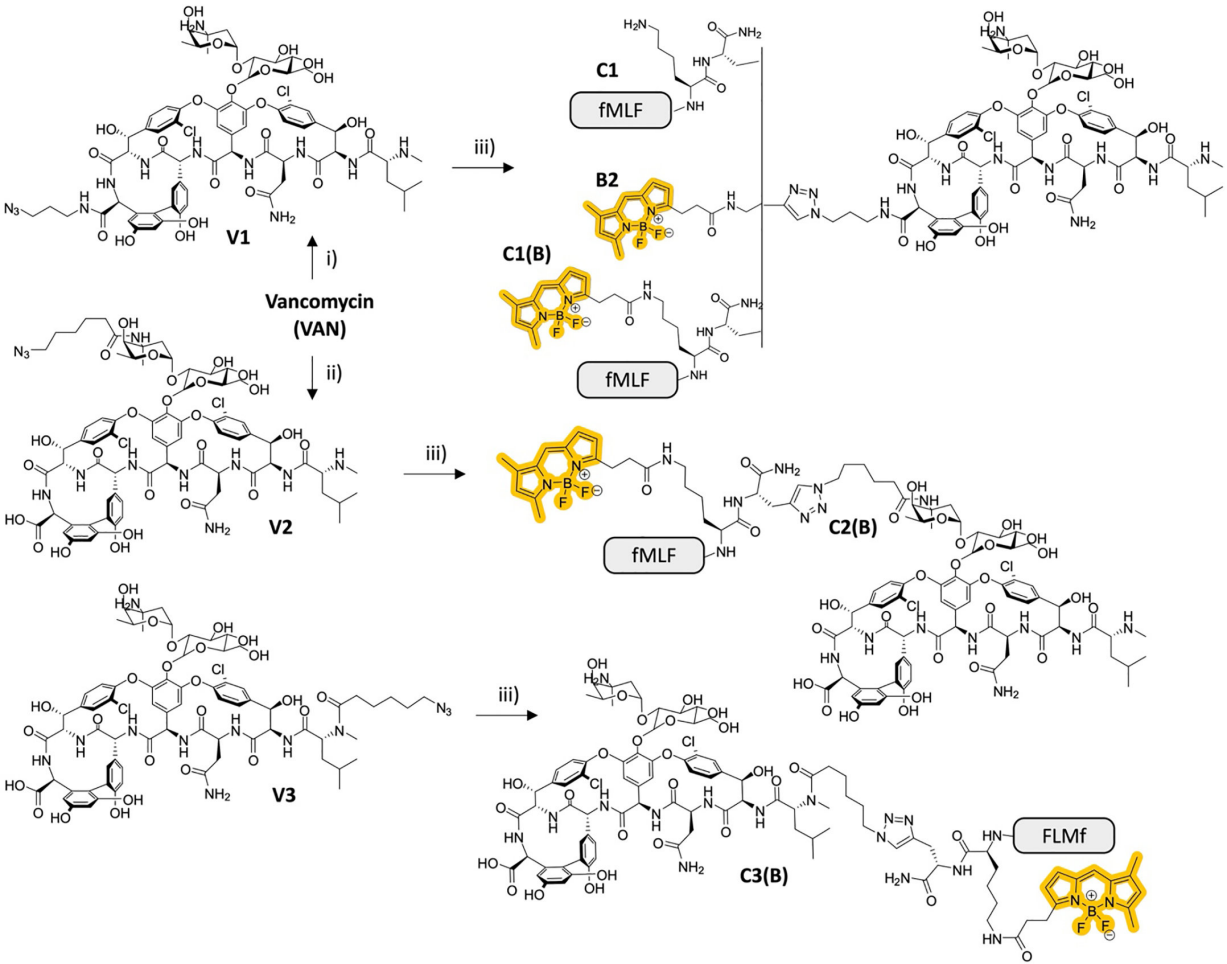
Synthesis of modified vancomycin derivatives. Reaction conditions: i) C‐terminal modification of vancomycin utilises PyBOP (1.5 equivalent (eq.)) and DIEA (3 eq.) in 40 mM DMSO, 5 min, 4 °C; then addition of 3‐azido‐1‐propanamine (2 eq.), 15 min, room temperature; ii) amine‐targeted modification of vancomycin utilises a micture of 6‐azidohexanoic acid (1.5 eq.), PyBOP (1.5 eq.) and DIEA (3 eq.) in 40 mM DMSO, 5 min, 4 °C; then addition of vancomycin (1 eq.), 30 min, room temperature, with separation via HPLC providing the vancosamine modified GPA and the N‐terminally modified GPA; iii) conjugation of fMLFK to the modified vancomycin derivatives using CuSO_4_ (2.5 eq.) and sodium ascorbate (5 eq.) in H_2_O; then addition of propargyl probes in 40% DMF, 1 h, room temperature. VAN is vancomycin; **C1** is vancomycin=(C‐linked) fMLFK; **B2** is BODIPY‐labelled vancomycin; **C1(B)** is BODIPY‐labelled vancomycin=(C‐linked) fMLFK; **C2(B)** is BODIPY‐labelled vancomycin=(V‐linked) fMLFK; **C3(B)** is BODIPY‐labelled vancomycin=(N‐linked) fMLFK; BODIPY dye (**B**) is highlighted in yellow.

To investigate conjugate binding, we utilised the fPep fMLFK‐Pra for incorporation into conjugates, including a propargylglycine (Pra—alkyne) residue to enable downstream coupling with azido‐modified GPAs (via the C‐terminus, N‐terminus and vancosamine moiety). We adjusted our previous synthetic strategy by linking the BODIPY fluorescent dye through its corresponding succinimide ester to the lysine (K) primary amine group of the fMLFK‐Pra peptide to generate fluorescent fPep conjugates. Click chemistry was used to link the BODIPY‐labelled fMLFK‐Pra (**B1**) to the three different azido‐modified GPAs (Scheme 1). Altogether, this provided the synthesis of five compounds: the unlabelled vancomycin=fMLFK conjugate (VAN‐C; **C1**), BODIPY‐labelled vancomycin (VAN‐(B*); **B2**) and three BODIPY‐labelled vancomycin=fMLFK conjugates (**C1(B)**, **C2(B)** and **C3(B)** (Scheme 1). Significant levels of methionine sidechain oxidation were noted in these reactions, which has previously been reported for such copper catalysed click chemistry conjugations [41].

**FIGURE 2:**
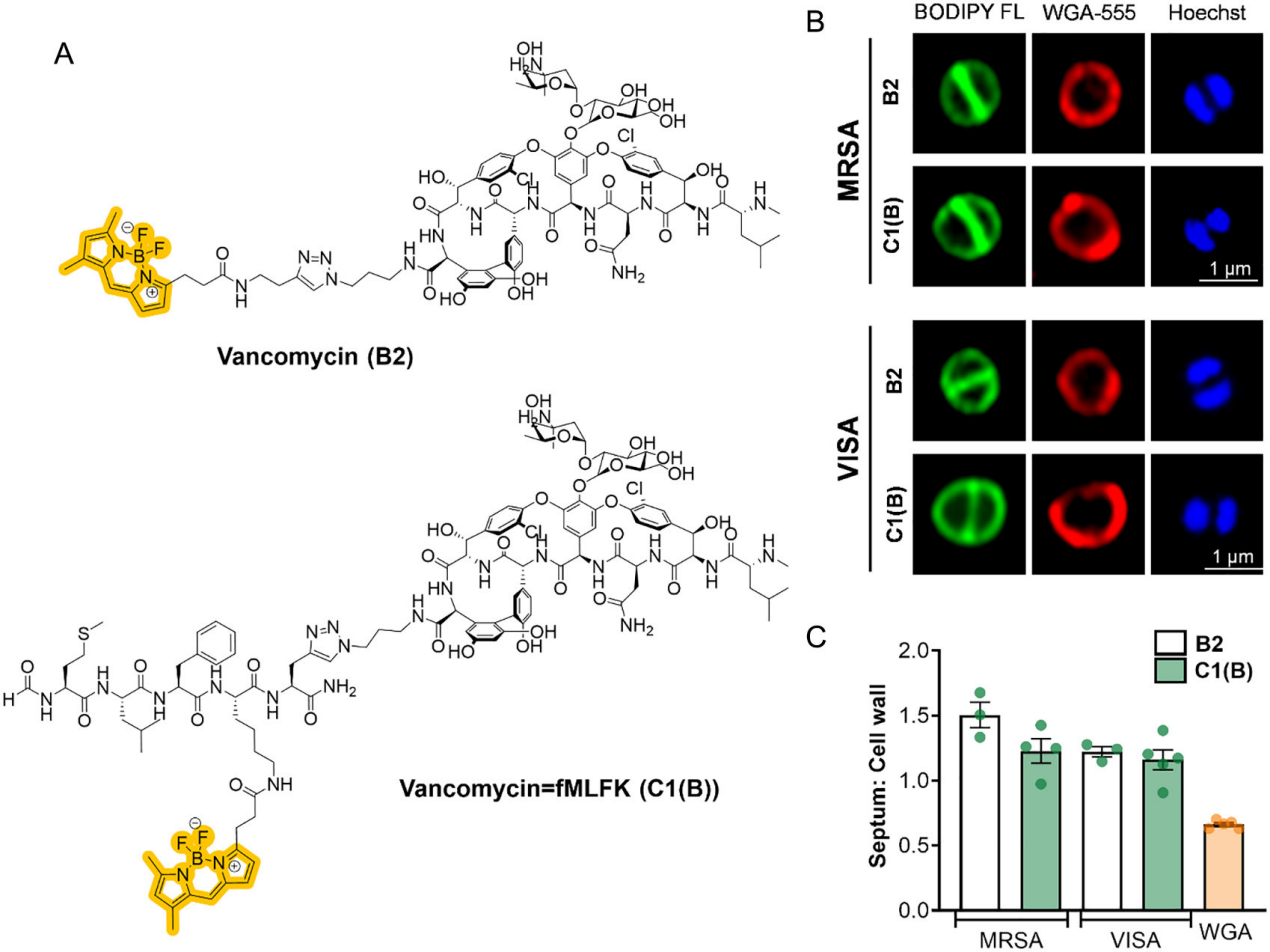
The immunotherapeutic binds to both MRSA A8090 and VISA A8094 strains. (A) Structure of BODIPY‐labelled vancomycin and fPep conjugated‐vancomycin. BODIPY labelling was performed at the C‐terminus of vancomycin, **B2**. fMLFK was linked at the C‐terminus of vancomycin, while BODIPY was labelled at the lysine residue of fMLFK, **C1(B)**. BODIPY dye (**B**) is highlighted in yellow. (B) Fluorescently labelled BODIPY vancomycin, **B2** or the vancomycin=(C‐linked)‐fMLFK conjugate, **C1(B)** imaged by Airyscan super‐resolution microscopy shows localisation to the wall and septum of MRSA and VISA clinical isolates. Scale bars are 1 μm; nuclear staining by Hoechst 33342; cell wall stained by Wheat germ agglutin (WGA‐555). (C) The average ratio in peak fluorescence intensity was calculated at the septum vs the wall of the *S. aureus* cell from Airyscan microscope images of at least 120 cells per condition across at least three biological replicates and plotted. Error bars are SEM.

To be able to compare conjugate binding between MRSA and VISA, we used paired clinical isolates for all our experiments. These paired clinical isolates consist of MRSA/VISA (A8090/94) strains, where MRSA is the vancomycin‐susceptible parent strain and VISA the resistant daughter strain isolated from the same patient before and after drug treatment that led to clinical failure [42, 43]. Firstly, we validated the binding of targeting element vancomycin to the surface of *S. aureus* using fluorescently labelled BODIPY: vancomycin (**B2 and C1(B)**) and vancomycin=fMLFK conjugates via microscopy (Figure 2A,B). Wheat germ agglutin (WGA) was used as a control for *S. aureus* cell wall staining, as WGA is known to bind to N‐acetylglucosamine (GlcNAc) residues present in Gram‐positive bacterial cell wall but does not bind strongly to the septum [44, 46], which is the binding site of vancomycin responsible for its antimicrobial activity [38]. The peak fluorescence intensity at the septum and the wall of *S. aureus* was used to calculate the median fluorescence ratio to compare binding, where ratio of more than 1 indicates preferential septum binding. A median ratio of 1.5 observed for vancomycin, and 1.3 for conjugate binding to MRSA (Figure 2C) [39]. This indicates higher fluorescence at the septum compared to the wall, although binding to the septum is reduced with the conjugate [39]. A similar decrease in septum binding was observed with the VISA strain for both vancomycin and the conjugate (Figure 2C), indicating a reduced amount of vancomycin reaching the septum, which is expected due to the thickening of the cell wall and increasing (D)Ala‐(D)Ala binding sites. This interaction mode was supported by the lower ratio of 0.6 in WGA controls, indicating greater binding to the cell wall than septum [39]. As this indicated that the binding of the conjugate to both MRSA and VISA were maintained, we then further assessed the antimicrobial activity of the conjugates and quantified the binding of the conjugates via flow cytometry.

**FIGURE 3:**
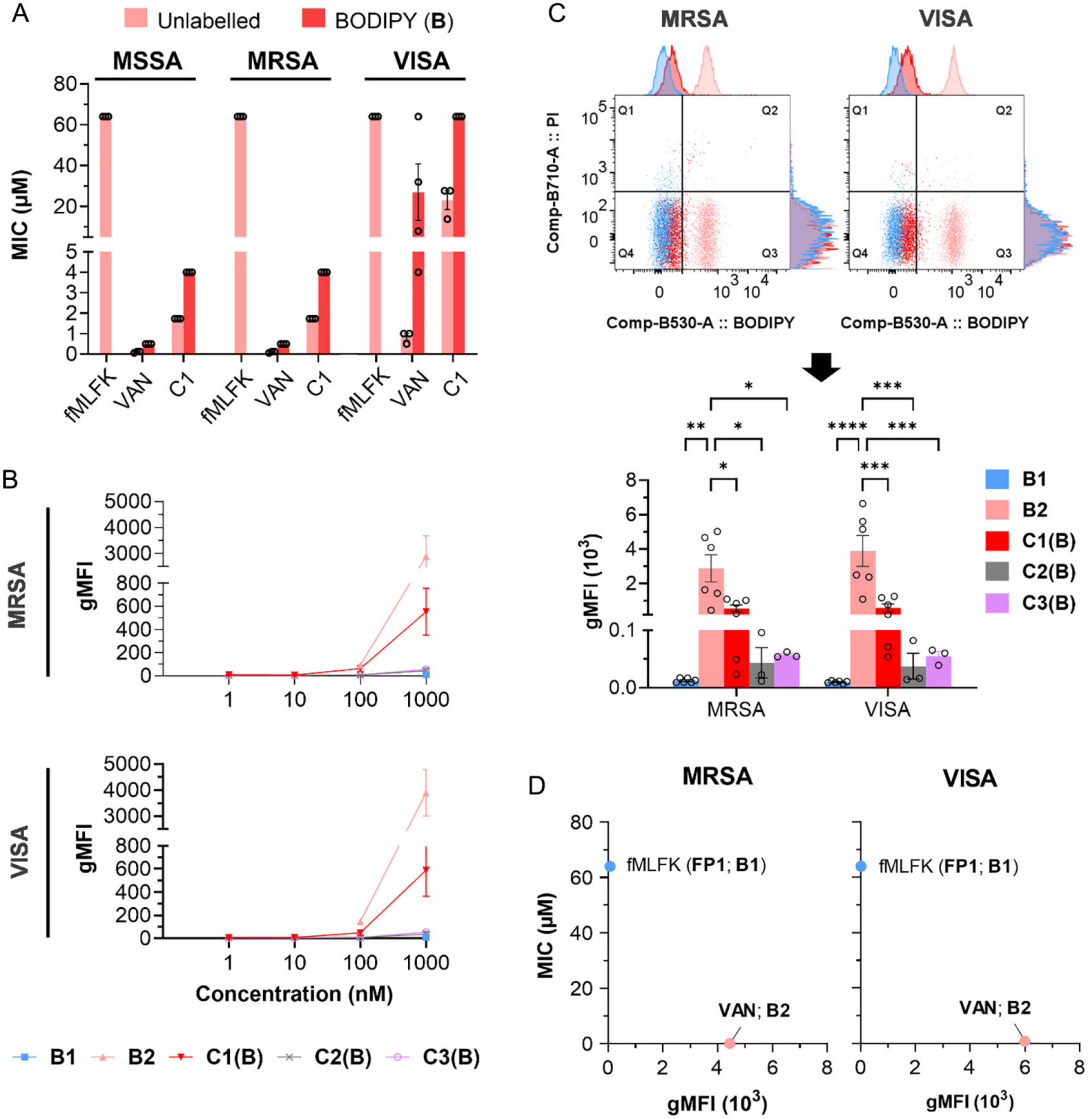
Fluorescently labelled vancomycin=(C‐linked)‐fMLFK showed reduced binding to clinical pair isolates MRSA A8090 and VISA A8094 compared to BODIPY vancomycin. (A) Conjugation of fPep to vancomycin and labelling with a fluorescent dye reduce antimicrobial activity against MSSA ATCC 29213, MRSA A8090 and VISA A8094 clinical isolates. Growth inhibition of clinical isolates of MSSA, MRSA and VISA in the presence of the conjugated compounds were determined via microbroth dilution assays. Treatments using unlabelled vancomycin (**VAN**, light red bars) and vancomycin=(C‐linked)‐fMLFK (VAN=fMLFK or **C1**, light red bars) were compared with BODIPY‐labelled: vancomycin (**B2**, red bars) and VAN=fMLFK (**C1(B)**, red bars). Growth was relative to the no protein control, after 24 h at 35 °C. Microbroth dilution assays were performed in biological replicates (*n* = 3); error bars are SEM. (B) Concentration gradient of vancomycin=fMLFK and vancomycin binding to *S. aureus* clinical isolates. Flow cytometry was performed to measure the binding levels of BODIPY‐labelled free vancomycin (**B2**, pink salmon), vancomycin=(C‐linked)‐fMLFK (**C1(B)**, dark red), vancomycin=(V‐linked)‐fMLFK (**C2(B)**, grey) and vancomycin=(N‐linked)‐fMLFK (**C3(B)**, brown). Treatments were performed at concentrations of 0, 1, 10, 100 and 1000 nM against MRSA A8090 (**A**), VISA A8094 (**B**). fMLFK (**B1**, grey) was used as a negative control in binding to cell surface of bacteria. Flow cytometry was performed in biological replicates (*n* = 3 or 6); geometric mean fluorescence intensity is gMFI; error bars are SEM. (C) Binding levels to resistant *S. aureus* isolates quantified by flow cytometry using BODIPY‐labelled: vancomycin (**B2**, light red bars) and vancomycin=(C‐linked)‐fMLFK (**C1(B)**, dark red bars). While the labelling of fluorescent BODIPY at the lysine of fMLFK shows better binding affinity of vancomycin to *S. aureus*, BODIPY‐labelled: vancomycin=(V‐linked)‐fMLFK (**C2(B)**, grey bars) and vancomycin=(N‐linked)‐fMLFK (**C3(B)**, brown bars) show low to no binding to MRSA and VISA clinical isolates. fMLFK (**B1**, blue bars) was used as a negative control for binding. Treatments were performed at a concentration of 1000 nM. Flow cytometry was performed in biological replicates (*n* = 3 or 6). Data were analysed using two‐way ANOVA and values were determined by Tukey’s post‐hoc tests. Statistical significance denoted by: *p ≤ 0.05, **p ≤ 0.01, ***p ≤ 0.001, ****p ≤ 0.0001 and ns (not significant) for *p* > 0.05. Propidium iodide is PI; geometric mean fluorescence intensity is gMFI; error bars are SEM. (D) MIC of vancomycin=fPep relative to its binding to surface of MRSA/VISA (A8090/94) strains. MIC of unlabelled vancomycin (**VAN**), vancomycin=fMLFK conjugate (**C1**) and free fMLFK (**FP1**) against resistant *S. aureus* isolates were compared to the binding levels of BODIPY‐labelled vancomycin (**B2**), vancomycin=fMLFK conjugate (**C1(B)**) and free fMLFK (**B1**) (as measured by gMFI at 100% peptide purity; Table S2) to surface of resistant *S. aureus* isolates. MIC values were reported in μM; gMFI is geometric mean fluorescence intensity; **VAN** is vancomycin (light red); **VAN=fMLFK** is vancomycin=(C‐linked)‐fMLFK (dark red); **fMLFK** (light blue).

To test the antibiotic efficacy of the conjugates against these *S. aureus* strains, we carried out standard Minimum Inhibitory Concentration (MIC) growth inhibition assays (Figure 3A, Table S1). Vancomycin inhibits growth of methicillin‐susceptible *S. aureus* (MSSA, ATCC 29213) and MRSA at low concentrations, compared to the higher concentrations required to inhibit the growth of VISA (Figure 3A, Table S1). We observed that growth inhibition required a 5–10 times lower concentration of vancomycin for MRSA A8090, and 8–16 times lower vancomycin concentration for VISA A8094 than had been reported by Sakoulas et al. [47] and Peleg et al. [42]. When fMLFK is attached to vancomycin (**C1**) the growth inhibition activity is reduced across all *S. aureus* strains (Figure 3A, Table S1). Nevertheless, this still indicated the ability of compounds to interact with bacteria using the targeting element vancomycin. Fluorescently labelled vancomycin (**B2**), and conjugates (**C1(B)**) modified with BODIPY dye resulted in a reduction in activity against all strains compared to the unlabelled compounds (Table S1). This suggests the presence of the fluorescent dyes restricts vancomycin from reaching its target at bacterial cell surface, similar to the lower inhibitory activity reported for fluorescently labelled vancomycin by Walker’s group [48]. The fPep, fMLFK, is known as a chemoattractant, and no antimicrobial activity was observed against any strains tested even at the highest concentration of 32 µM for free fMLFK (**FP1**). Although vancomycin has reduced antibiotic activity against the VISA strain, the thickened cell wall with decoy binding sites (Figure 1B) would suggest a greater propensity for actual binding of vancomycin to the bacterial cell surface. Indeed, Pereira et al. [49] have previously shown an increase in in vivo vancomycin binding capacity to the vancomycin‐resistant strains and reported the lack of correlation between the increased binding of vancomycin and its antimicrobial activity.

A recent study by Zhang et al. [50] incorporated the use of fluorescent probes to visualise the site of the interaction of antibiotics towards both Gram‐positive and Gram‐negative strains that was further quantified by flow cytometry. Here, we use BODIPY‐labelled compounds to understand the binding to the resistant *S. aureus* clinical isolates, as well as to establish a general flow cytometry assay to determine the interaction of compounds to bacteria rather than relying on inhibition activity as a proxy for binding (Figure S1). *E. coli* ATCC 25922 was used as a negative control strain as this has no exposed binding sites for vancomycin binding (Figure S2), while the chemoattractant fMLFK was used as negative control compound as this at most very limited binding to the *S. aureus* isolates (Figure S2). Concentration gradients of free vancomycin and fPep conjugates linked via the C‐terminus of vancomycin were performed from 1 to 1000 nM across all the clinical bacterial isolates, where the binding of compounds to the isolates increased with increasing concentration as expected (Figures 3B and S3). The nanomolar concentration range was performed up to 1000 nM, a range that is much lower compared to the MIC of these immunotherapeutics. This range provides an effective concentration for the attached fPep (sequence specific) to establish a chemotactic gradient that can recruit neutrophils (donor dependent) to target bacteria without risking direct bacterial killing and lysis [51, 54].

Using this flow cytometry method, BODIPY fluorescence was detected across all *S. aureus* strains when incubated with labelled vancomycin (Figure 3C). Higher BODIPY fluorescence indicates increased binding, especially for VISA, which may be due to increased binding of vancomycin to the thickened cell wall of VISA containing decoy binding sites. A decrease in BODIPY fluorescence was observed when treated with vancomycin=fMLFK conjugates compared to free vancomycin in MRSA (C‐linked conjugate, *p*‐value = 0.0216; V‐linked conjugate, *p*‐value = 0.0226; N‐linked conjugate, *p*‐value = 0.0237) and in VISA (C‐linked conjugate, *p*‐value = 0.0003; V‐linked conjugate, *p*‐value = 0.0005; N‐linked conjugate, *p*‐value = 0.0006) (Figure 3C). This corresponds to the decrease in growth inhibition activities observed (Figure 3A, Table S1). Meanwhile, studies by Payne et al. [39] have previously reported a reduced antimicrobial activity for fMLFG conjugates of vancomycin linked via the vancosamine primary amine (V‐linked) and no antimicrobial activity observed when linked via the N‐terminal amine moiety (N‐linked). A similar labelling strategy was performed to introduce BODIPY at the lysine residue of fMLFK and then link this cargo to the vancosamine primary amine (V‐linked, **C2(B)**) and amino‐terminal GPAs (N‐linked, **C3(B)**) (Scheme 1, Figures S4 and S5). The resultant compounds vancomycin=(V‐linked)‐fMLFK (**C2(B)**) and vancomycin=(N‐linked)‐fMLFK (**C3(B)**) showed an approximately 13× and 10× decrease in binding to MRSA and an approximately 16× and 11× decrease in binding to VISA when compared to binding of vancomycin=(C‐linked)‐fMLFK (**C1(B)**) as determined by flow cytometry (*p*‐value > 0.05) (Figure 3B,C). The binding of the compounds normalised with compound purity was also compared to direct antimicrobial activity, where the conjugates that show antimicrobial activities against *S. aureus* clinical isolates also demonstrate higher levels of binding across the cell surface of *S. aureus* (Figure 3D, Table S2).

### 2.2 Linkage Location and Linker Length are Important for Vancomycin Binding to Cell Surface *S. aureus* Clinical Isolates

**SCHEME 2:**
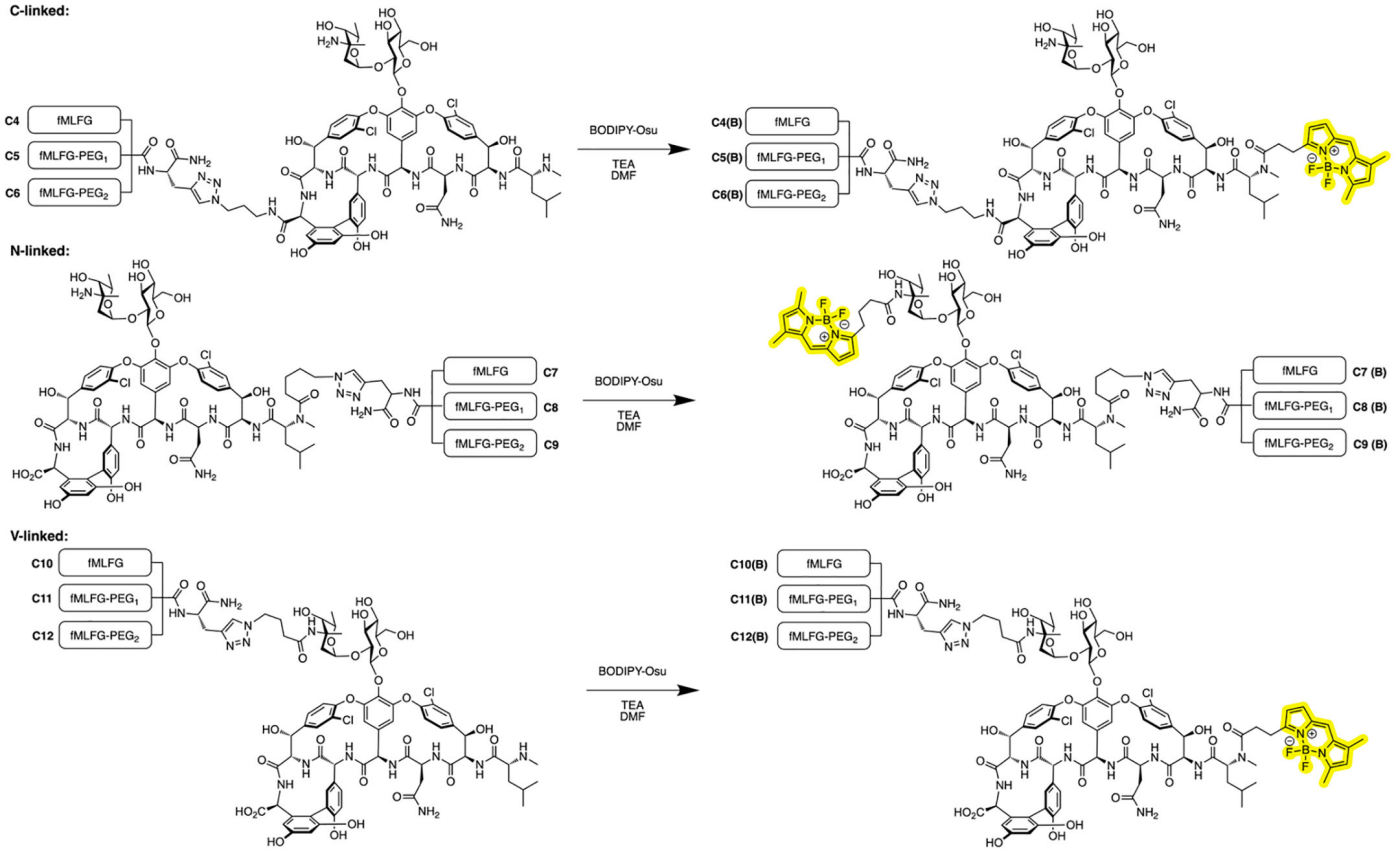
Labelling of the vancomycin=fPep library with BODIPY on the vancosamine or N‐terminal (D)‐leucine residue. Each vancomycin=fMLFG conjugate (1 eq.) was mixed with BODIPY FL NHS Ester (1.5 eq.) and TEA (4 eq.) in DMF. Reactions were left overnight and purified by solid‐phase extraction using a gradient in acetonitrile from 10% to 100% acetonitrile. **C4(B)**, **C5(B)** and **C6(B)** are BODIPY‐labelled vancomycin=(C‐linked) fMLFG; **C7(B)**, **C8(B)** and **C9(B)** are BODIPY‐labelled vancomycin=(N‐linked) fMLFG; **C10(B)**, **C111(B)** and **C12(B)** are BODIPY‐labelled vancomycin=(V‐linked) fMLFG; BODIPY dye (**B**) is highlighted in yellow.

**FIGURE 4:**
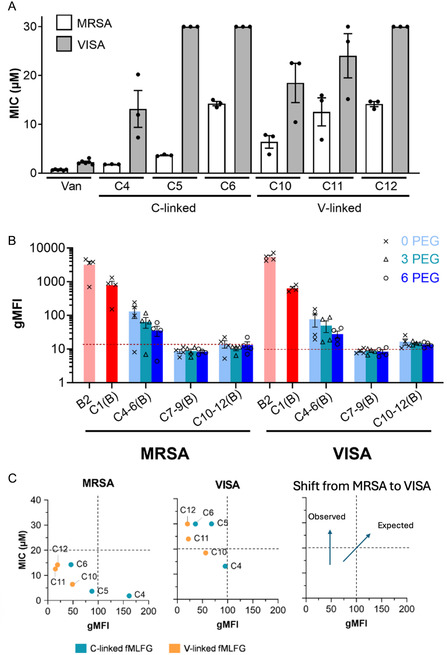
Conjugation of fPep at the C‐terminal of vancomycin is crucial for its interaction with cell surface of MRSA and VISA. (A) Linkage to vancomycin at different sites changes the antimicrobial activity against VISA. Three different length PEG linkers were trialled: no linker (0PEG), 3 PEG units (3PEG) or 6 PEG units (6PEG) at these attachment sites, leading to different vancomycin=fMLFG conjugations: C‐linked (**C4‐6**) and V‐linked (**C10‐12**). Using a microbroth dilution assay, the growth of clinical isolates of MRSA and VISA in the presence of the conjugated compounds were determined. Growth was relative to the no protein control, after 24 h at 35 °C. Microbroth dilution assays were performed in biological replicates (*n* = 3); error bars are SEM. MIC of compounds against MRSA A8090 was previously reported [39]. ‍(B) Binding of vancomycin=fMLFG analogues to resistant *S. aureus* clinical pair isolates. Vancomycin modifications were designed to attach fMLFG to vancomycin using different PEG linker lengths of 0 spacers (light blue bars), 3 spacers (teal bars) and 6 spacers (dark blue bars) at the C‐terminus (C‐linked, **C4‐6**), N‐terminus (N‐linked, **C7‐9**) and vancosamine residue (V‐linked, **C10‐12**). Binding levels to MRSA and VISA quantified by flow cytometry were compared between BODIPY‐labelled vancomycin=fMLFG (**C4‐12(B)**) at concentrations of 1000 nM. Binding of vancomycin conjugates was also compared with vancomycin (**B2**) and C‐linked fMLFK (**C1(B)**). fMLFK (**B1**, data not shown) was used as a negative control for binding, and the lower boundary of binding is indicated by the red dotted line. Flow cytometry was performed in biological replicates (*n* = 4); geometric mean fluorescence intensity is gMFI; error bars are SEM. (C) MIC of vancomycin=fMLFG analogues relative to their binding to surface of MRSA/VISA (A8090/94) strains. MIC of unlabelled vancomycin=fMLFG analogues against resistant *S. aureus* isolates were compared to the binding levels of BODIPY‐labelled vancomycin=fMLFG analogues (as measured by gMFI at 100% peptide purity; Table S2) to surface of resistant *S. aureus* isolates. The interaction and activity of conjugates between MRSA and VISA were expected to shift from lower left quadrant (low binding, high activity) to upper right quadrant (high binding, low activity), however the shift was observed towards the upper left quadrant (low binding, low activity). MIC values were reported in μM; gMFI is geometric mean fluorescence intensity; vancomycin=(C‐linked)‐fMLFG (**C4‐6**, teal); vancomycin=(V‐linked)‐fMLFG (**C10‐12**, orange).

In previous work [39], we had generated a library of vancomycin=fPep conjugates (**C4‐12**) that incorporated different PEG linker lengths as spacers between the fPep cargo and 3 different sites on vancomycin (C‐terminus (C‐linked), N‐terminus (N‐linked) or vancosamine residue (V‐linked) (Scheme 2, Figure 1A)). This panel of compounds showed a range of antimicrobial activities against MRSA (Figure 4A), with the introduction of the fPep retaining more activity when conjugated to the C‐terminus of vancomycin and with improved activity seen for short linkers between vancomycin and the fPep cargo. Now with a method to interrogate the binding of these molecules to bacteria, we extended this work to include the comparison between resistant *S. aureus* paired clinical isolates MRSA A8090 and VISA A8094. Similar trends in growth inhibition of VISA were observed when compared to MRSA (Figure 4A) [39], although the antimicrobial activity of these conjugates was reduced compared to their activity towards MRSA as previously noted by Payne et al. [39]. fMLFG conjugation at the vancosamine residue of vancomycin showed reduced antimicrobial activity, while N‐terminal conjugation almost completely abrogated antimicrobial activity towards both MRSA [39] and VISA (Figure 4A). We then modified this library (**C4‐12**) by adding a fluorescent BODIPY group by coupling BODIPY succinimide ester on one of the free amino groups located at either vancosamine or N‐terminal (D)‐leucine residue (Scheme 2). We generated this second library (**C4‐12(B)**) (Scheme 2), which was purified by solid‐phase extraction and characterised by LCMS (Figures S6–S14).

Fluorescently labelled conjugates were then used to quantify compound binding to both MRSA and VISA (Figures S15–S17). BODIPY conjugation to fMLFG linked via the C‐terminus of vancomycin (**C4‐6(B)**) (Figure 4B) showed that higher fluorescence levels correlate with increased binding of vancomycin=(C‐linked)‐fMLFG to MRSA (Figure 4B) and slightly reduced binding observed towards VISA (Figure 4B). This was somewhat unexpected as VISA contains decoy vancomycin binding sites that increases the binding of vancomycin to the bacterial cell wall. While studies have shown that the binding of vancomycin to such resistant strains is not correlated with MIC, the reduced vancomycin binding that we observed with VISA could be due to the compositional changes around the septum of the resistant strain reducing the diffusion rate of vancomycin and limiting the amount that can reach the septum of VISA [49]. The reduced vancomycin binding observed with VISA could also be due to the size of the conjugate, which reduces the accessibility to the additional decoy binding sites deeper within the cell wall matrix due to steric effects.

A decrease in BODIPY fluorescence was observed for vancomycin=(C‐linked)‐fMLFG molecules with increasing length PEG spacers (Figure 4B) for both *S. aureus* strains. This reduced binding affinity correlates with the increasing bulk of the analogue affecting the interaction of vancomycin with the cell surface of *S. aureus*. Little to no binding was observed for compounds in which fMLFG was linked via the N‐terminus of vancomycin (**C7‐9(B)**) or to the vancosamine residue of vancomycin (**C10‐12(B)**) (Figure 4B), suggesting that conjugation of fMLFG (and the BODIPY dye) at these sites greatly reduced the binding of vancomycin to targeting site on the *S. aureus* cell surface. The conjugates displaying high antimicrobial activities against *S. aureus* clinical isolates displayed correspondingly high binding affinities (after normalisation with compound purity) to the cell surface of *S. aureus* (Figure 4C, Table S2). Comparing the MRSA and VISA results, we had anticipated higher binding to VISA due to the presence of D‐Ala‐D‐Ala decoy sites, whilst instead we observed the shift upwards in terms of MIC and no shift from left to right in terms of binding (Figure 4C).

## 3 Discussion

In this study, we compared the results of antimicrobial assays with a flow cytometry‐based assay binding assay, with the binding of vancomycin and its conjugates to *S. aureus* unable to be decoupled from direct antimicrobial activity; this was unexpected for the VISA strain due to additional GPA binding sites on the cell surface of this strain (Figure 1B). This means that fluorescence studies (Figure 2), flow cytometry‐based methods (Figures 3 and 4) and broth microdilution assays can all be used to understand the binding of fPep‐GPA conjugates to the surface of *S. aureus*. The likelihood of the fPep cargo inhibiting GPA binding and activity through a peptide/peptide interaction similar to the cognate GPA/(D)Ala‐(D)Ala interaction is highly unlikely, with numerous studies having demonstrated that very small changes to the (D)Ala‐(D)Ala target can have major effects on binding and hence activity of GPAs [55, 57]. Examples relevant here include the dramatic losses in activity seen with alterations to the methyl sidechains of the (D)Ala‐(D)Ala dipeptide target, with a total loss of GPA binding seen when the stereochemistry of the terminal Ala is altered from (D) to (L) [58]. Addition of a methyl group at the penultimate (D)Ala (i.e. an Aib residue) also results in complete loss of binding, whilst removal of either methyl group (i.e. a Gly residue) causes 10‐ to 100‐fold losses in binding affinity [58]. Thus, it is clear that the fMLFK sequence bearing (1) all (L)‐configured residues, (2) being presented in the wrong orientation (i.e. terminating in the peptide N‐terminus rather than the C‐terminus seen with (D)Ala‐(D)Ala) and (3) possessing large, bulky residues, shows no similarity to the native target and hence cannot compete directly for GPA binding with (D)Ala‐(D)Ala molecules on the bacterial cell wall.

An immunotherapeutic strategy using fPep‐GPA conjugates requires the addition of a fluorescently labelled formylated peptide at sites that do not interfere with the hydrogen bonding interactions between vancomycin and its (D)Ala‐(D)Ala target (i.e. C‐terminal carboxyl group, primary/secondary amine groups) [50, 59, 60]. We observed that the modification of vancomycin with fPep conjugation at the C‐terminus of vancomycin was less detrimental to antimicrobial activity and binding to the cell wall of *S. aureus* as compared to fPep conjugation at the N‐terminus and vancosamine moieties. The antimicrobial activity and the binding affinity seen with the C‐linked conjugates and resistant *S. aureus* strains were reduced when compared to vancomycin alone, likely due to the bulkiness of the fPep and dye conjugate affecting the interaction of vancomycin with the cell surface of *S. aureus*. Therefore, the selection of fluorophores with low molecular weight and minimal charge is preferable to maintain antimicrobial activity like unlabelled parent antibiotics targeting the cell surface of bacteria [50, 61, 62]. Other glycopeptide antibiotic conjugates have previously showed strong bias for modification at the C‐terminus to improve activity towards vancomycin‐resistant strains, which also shows the more benign nature of this site for cargo conjugation in GPA conjugates. A range of modifications seem to be broadly tolerated at the C‐terminus of the GPA and have largely included cationic and hydrophobic moieties to improve selectivity and activity against MRSA, VISA, VRE or to target Gram‐negative strains [63, 65]. The N‐terminus of vancomycin, on the other hand, plays an important role in binding to the Lipid II target, with analogues bearing N‐terminal modifications highly susceptible to loss of antimicrobial activity [66]. Studies reporting successful N‐terminal modifications include the addition of lipo‐cationic modifications [65], cell‐penetrating peptides [66], N‐alkylation [64] or the introduction of sulfonium moieties, groups dissimilar to fPeps. Modifications that are large and hydrophobic appear to occlude binding of the GPA conjugate to Lipid II, which is likely relevant for the conjugates tested here. Similar effects appear when modifying the vancosamine residue, with small hydrophobic groups (such as a chloro‐biphenyl group) leading to increase dimerisation and antimicrobial activity [33, 61, 62]. There is no reported precedent for the conjugation of large molecules such as the fluorescently labelled formylated peptides tested in this study, suggesting that the same trends seen with N‐terminal modification also holds for linkage via the vancosamine moiety. Taken together this suggests C‐terminal conjugates directly linked to the GPA C‐terminus as the optimal design for future studies.

## 4 Conclusion

Vancomycin=fPep immunotherapeutics demonstrate that modified antibiotic derivatives can be used as elements to target resistant *S. aureus* bacteria. We have established a flow cytometry assay to determine the binding levels of vancomycin=fPep conjugates to the bacterial surface, which has revealed that the most active immunotherapeutics are linked to their fPep cargoes via the carboxyl‐terminus of vancomycin. It is important to note that our immunotherapeutic strategy does not rely on the antimicrobial action of vancomycin (and indeed such activity is undesirable), hence the rationale for developing a flow cytometry method to measure antibiotic binding to the bacterial cell surface. While such binding is generally reduced upon conjugation of fPep and fluorescent labelling, the residual interaction between vancomycin and the bacterial cell surface can still be harnessed for the delivery of immunotherapeutic payloads to target bacteria. Future studies will improve our understanding of innate immune cell recruitment and activation when using such conjugates, which provide an alternative to circumvent antimicrobial resistance and immune evasion mechanisms seen for resistant bacterial infections [67, 71].

## 5 Materials and Methods

### 5.1 Vancomycin Functionalisation

Azide functionalisation of vancomycin for click chemistry was performed as previously described in Payne et al. [39]. Pre‐activation of the C‐terminal carboxylic acid of vancomycin HCl (1 eq.) was performed with COMU (1 eq.) and triethylamine (TEA, 1 eq.) in DMF at ∼30 mM [72] for 5 min. 3‐azido‐1‐propanamine (2 eq.) or propargylamine (2 eq.) were then added, stirred overnight and analysed by liquid chromatography‐mass spectrometry (LCMS). The product was subjected to diethyl ether (Et_2_O) precipitation, washed twice with Et_2_O and purified by preparative RP‐HPLC. Meanwhile, vancomycin functionalisation of the N‐terminal amine or on vancosamine moiety was performed by pre‐activation of 5‐azidopentanoic acid (1.1 eq.) or 6‐azidohexanoic acid (1.1 eq) (for **C2(B)** and **C3(B)**) with COMU (1.1 eq.) and TEA (2 eq.) in DMF for 5 min. After pre‐activation, the 5‐azidopentanoic acid or 6‐azidohexanoic acid was added to a solution of vancomycin (HCl salt, 1 eq.) in DMF at ∼20–50 mM, stirred overnight and analysed by LCMS. The product was further precipitated in Et_2_O, washed twice with Et_2_O and purified by preparative RP‐HPLC. The subsequent LCMS analysis confirmed the loading of azidopentanoyl or azidohexanoyl on the amines on the N‐terminus or the vancosamine moiety by small‐scale hydrolysis.

### 5.2 Solid‐Phase Peptide Synthesis on SynPhase Lanterns and Rink‐Amide NovaPEG Resins

Peptides were synthesised on SynPhase PSDRAM lanterns (37 µmol/lantern) and Rink‐amide NovaPEG resin (Novabiochem) (681.8 mg, loading 0.3 mmol) using an Fmoc strategy [73] as described in Payne et al. [39]. Lanterns and resins were swelled in dichloromethane (DCM) for 30 min and flushed with DMF for 3–5 times (3 min for lanterns and 30 s for resins). Fmoc deprotection steps were carried out in 20% (v/v) piperidine/DMF for 20 min, followed by four DMF washes as described above. Coupling conditions using lanterns were carried out with 4 eq. amino acid, 3.8 (2)‐(6‐chloro‐1H‐benzotriazole‐1‐yl)−1,1,3,3‐tetramethylaminium hexafluorophosphate (HCTU) and 8 eq. DIEA for 1 h with shaking, followed by four DMF washes as described above. Split/mix peptide coupling using lanterns was performed to generate the formylated peptide library. Meanwhile, coupling reactions on resins were performed using pre‐activated amino acids (2 eq.) in HCTU (1.95 eq.) and DIEA (4 eq.). Peptides were cleaved from lanterns using mixture of trifluoroacetic acid/ water/ triisopropyl silane or TFA/H_2_O/TIPS (95/2.5/2.5; v/v’/v”) for 1 h. Meanwhile, resins were first dried with DCM prior to peptide cleavage under the same conditions. Lanterns and resins were filtered and washed with TFA, where the remaining solutions were concentrated under N_2_, precipitated in cold Et_2_O, washed three times with cold Et_2_O and air‐dried overnight. The isolated peptides were then analysed by LCMS and purified by preparative RP‐HPLC.

### 5.3 Formylation, Formylated‐Methionine Coupling and Acetylation on Solid Support

Formylation and formylated‐methionine coupling were carried out after the final deprotection steps. Formic acid or fMet‐OH (3 eq.) and N‐Hydroxysuccinimide or HOSu (3 eq.) were dissolved in DCM at 0 °C with stirring followed by gradual addition of EDC.HCl (3 eq.) before the lanterns were added. DIEA (1 eq.) was then added to maintain a basic pH; the coupling reaction was left overnight at room temperature. Acetylation was carried out in mixture of acetic anhydride/DIEA/DMF or Ac_2_O/DIEA/DMF (5:5:90) with shaking overnight following the final deprotection steps.

### 5.4 Vancomycin=fPep Conjugation

Conjugation of fPep to azide‐functionalised vancomycin via click chemistry was performed as previously described in Payne et al. [39]. Reactions were performed at ∼3 mM concentration, where the fPep (1 eq.) was dissolved in DMF (50 μL/mg) and added to azide‐functionalised vancomycin (1.1 eq.) dissolved in aqueous sodium ascorbate buffer (22 mM, pH 8.0) [72]. An aqueous solution of CuSO_4_ (3 eq., 0.1 M) was added and that formed a precipitate, with the reaction incubated with shaking for 1 h. The reaction was analysed by LCMS, and the product was purified by preparative RP‐HPLC.

### 5.5 BODIPY Labelling of Vancomycin=fPep Conjugates

Conjugated vancomycin=fPeps were labelled with BODIPY FL NHS ester (succinimidyl ester) (Thermo Fisher Scientific, USA) and purified via solid phase extraction (SPE) prior to assessment of vancomycin binding to surface of bacteria via flow cytometry. Strata‐X SPE columns (Phenomenex) were pre‐activated with 3 mL of methanol (MeOH) with 0.1% (v/v) formic acid (FA) and washed with 3 mL of water with 0.1% (v/v) FA. Columns were equilibrated with 1 mL of 10% (v/v) acetonitrile (ACN). Compounds where fMLFG were previously conjugated at the N‐terminus, vancosamine moiety‐ or C‐terminus of vancomycin, were each mixed with BODIPY FL NHS Ester (1.5 eq.) and TEA (4 eq.) in DMF, followed by DMF removal in a vacuum concentrator overnight. The concentrated reactions were each solubilised in 50% (v/v) ACN, further diluted to ∼12.5% (v/v) ACN and individually loaded onto the activated and equilibrated SPE columns. Loaded compounds were then eluted with 2 mL of ACN solution at different concentrations (10‐100% (v/v)) and subsequently analysed by LCMS to determine the fractions containing BODIPY‐labelled compounds. Meanwhile, BODIPY was introduced at the C‐terminus of vancomycin and fPep‐conjugated vancomycin to minimise the effect of the fluorescent dye upon the binding of vancomycin to the bacteria cell surface when assessed by flow cytometry. BODIPY was first used to label the fPep sequence, fMLFK or propargylamine using BODIPY FL NHS Ester (1.1 eq.) in DMF (5 mM), generating BODIPY‐alkyne compounds. TEA (1 eq.) was then added to complete the coupling reactions before generating BODIPY‐labelled vancomycin and conjugates via click chemistry between the BODIPY‐alkyne and azide‐functionalised vancomycin. The products were analysed by LCMS.

### 5.6 LCMS Analysis and Preparative RP‐HPLC Conditions

LC/MS analyses were carried out as described in Payne et al. [39] using a Shimadzu High Performance Liquid Chromatograph coupled to a Mass Spectrometer LCMS‐2020 (Supporting Information, operating both in positive and negative mode) equipped with an SPD‐20A Prominence Photo Diode Array Detector and a LC‐20AD solvent delivery module running the Lab Solutions v5.97SP1 software. RP‐HPLC purifications were performed as described in Payne et al. [39] using a Shimadzu High Performance Liquid Chromatograph equipped with an SPD‐M20A Prominence Photo Diode Array Detector and two LC‐20AP pumps running the Lab Solutions v5.97SP1 software.

### 5.7 Bacterial Strains, Media and Culturing Conditions

The bacterial clinical isolates used in this work are listed in Table 1 and were stored in brain heart infusion (BHI) broth (Becton, Dickinson and Company, USA) with 20% (v/v) glycerol at −80 °C. These bacterial strains were streaked from glycerol stocks onto BHI agar or broth and grown overnight (16 to 18 h) at 35 °C with shaking. Meanwhile, bacteria were grown in cation‐adjusted Mueller Hinton Broth II (MHB) (Becton, Dickinson and Company, USA) for antimicrobial susceptibility test via broth microdilution.

**TABLE 1:** List of bacterial clinical isolates. MSSA is methicillin‐susceptible *S. aureus*, MRSA is methicillin‐resistant *S. aureus*, VISA is vancomycin‐intermediate *S. aureus*, and ATCC is American Type Culture Collection.

Species	Resistance	Abbreviation	Strains
*Staphylococcus aureus*	—	MSSA	ATCC 29213
	Methicillin	MRSA	A8090
	Vancomycin‐intermediate	VISA	A8094
*Escherichia coli*	—	*E. coli*	ATCC 25922

### 5.8 Imaging the Binding of Vancomycin to Surface of *S. aureus*

Visualisation of the binding of compounds to the surface of bacterial strains was performed as previously described in Payne et al. [39]. *S. aureus* clinical paired isolates MRSA/VISA (A8090/94) were grown to mid‐exponential phase, where cultures were adjusted to OD_600_ of 1.00 (∼2 × 10^8^ CFU/mL) and treated with 16 μM of the BODIPY‐labelled compounds: vancomycin (**B2**), fMLFK (**B1**), or antibiotic‐vancomycin=fMLFK conjugate (**C1(B), C2(B)**) for 30 min at room temperature. Treated cells were washed by centrifugation (370 × *g*) 3 times to remove unbound compound before resuspension in fresh phosphate buffered saline (PBS). Samples were imaged using a Zeiss LSM 980 Airyscan 2 (Carl‐Zeiss, Jena, Germany) with a 63× oil C‐PlanApo 1.4 NA objective using the optimal super‐resolution mode. Images captured were analysed using the FIJI image processing package within ImageJ for analysis of more than 120 cells for each treatment across three biological replicates. The fluorescence intensity of the compounds bound to the septum and cell wall of each cell was determined where the average ratio in peak fluorescence intensity for each cell was calculated. All data was then visualised using the GraphPad Prism v9 software.

### 5.9 Antimicrobial Susceptibility Test via Broth Microdilution

Antimicrobial susceptibility of the unlabelled and labelled vancomycin=fPep conjugates was determined via broth microdilution according to the Clinical and Laboratory Standards Institute (CLSI) guidelines. ATCC strains of *S. aureus* (ATCC 29213) and *E. coli* (ATCC 25922) and clinical paired isolates MRSA/VISA (A8090/94) were treated with compounds listed in Table 2. Compounds were prepared as twofold serial dilutions at a final DMSO concentration of 0.2% (v/v). The assays were set up in 96‐well clear, flat‐bottom polystyrene microplates (Greiner Bio‐One, Germany) by adding bacterial cells adjusted to 5 × 10^5^ CFU/mL in 90 μL cation‐adjusted MHB, followed by the addition of 10 μL serially diluted compounds in increasing concentrations. Plates were covered with Breathe‐Easy permeable, polyurethane sealing membranes (Diversified Biotech, Sigma–Aldrich, USA). Growth inhibition was observed for 24 h at 35 °C. The minimum inhibitory concentration (MIC) was determined by the lowest concentration of an antimicrobial compound that inhibits visible growth of bacteria in broth dilution. The average of three biological replicates were plotted using the GraphPad Prism v10.4.1 software.

**TABLE 2:** List of unlabelled and labelled compounds for MIC determination via antimicrobial susceptibility test. Compounds were labelled with BODIPY dye. The fPep sequence used for conjugation to vancomycin via click chemistry is fMLFK. B* is BODIPY, and C is C‐terminal.

Compound	Dye	Abbreviation	Reference
Vancomycin=(C‐linked) fMLFK	—	**C1**	[39]
	BODIPY	**C1(B)**	[39]
Vancomycin	—	**VAN**	—
	BODIPY	**B2**	[39]
fMLFK	—	**FP1**	[39]
	BODIPY	**B1**	[39]

### 5.10 Flow Cytometry Quantification of BODIPY‐Labelled Compounds Bound to *S. aureus* Clinical Isolates

Clinical paired isolates MRSA/VISA (A8090/94) were treated with compounds labelled with BODIPY for fluorescence detection via flow cytometry (Table 3). The ATCC strain for *E. coli* (ATCC 25922) was subjected to the treatment as a negative control for targeting by vancomycin and fPep. Cell concentrations were first adjusted to 2 × 10^8^ CFU in 100 μL PBS for each treatment condition. Bacteria were then treated with labelled‐compounds in 10‐fold concentration series (1–1000 nM), while fMLFK (**B1**) was used as negative control. fMLFK was used to ensure consistent labelling of fluorescent dyes at the lysine residue of the fPep sequence while minimising the bulk effect of the fluorescent dye during the binding assay via flow cytometry. After 30 min of treatment of the bacteria with compounds, all subsequent steps were performed at 4 °C. Treatment was followed by washing twice using PBS with 1 mM ethylenediamine‐tetraacetic acid (EDTA) (VWR Chemicals BDH Prolabo, Belgium), with samples centrifuged at 3000 rpm for 4 min to remove unbound compounds from the bacterial cells. Bacterial pellets were resuspended in PBS with 1 mM EDTA and then incubated with 5 µg/mL propidium iodide (Sigma–Aldrich, USA) for 5–10 min for viability discrimination when acquired by flow cytometry.

**TABLE 3:** Compounds labelled with fluorescent dye BODIPY to assess their binding to *S. aureus* clinical paired isolates. The fPep sequences used for conjugation to vancomycin via click chemistry are fMLFK and fMLFG. Vancomycin=fMLFK conjugates were labelled with BODIPY dye at the lysine of the fMLFK **(C1(B), C2(B), C3(B))**, while SPE was used to label BODIPY on vancomycin=fMLFG conjugates **(C4(B)** to **C12(B))**. B* is BODIPY, N is N‐terminal, V is vancosamine, C is C‐terminal, and PEG is polyethylene glycol.

Compound	Abbreviation	Length of PEG spacer between conjugates	Position of BODIPY labelling	Reference
fMLFK	**B1**	—	Lysine residue of fMLFK	[39]
Vancomycin	**B2**	—	C‐terminal of vancomycin	[39]
Vancomycin= (C‐linked) fMLFK	**C1(B)**	0 spacers	Lysine residue of fMLFK	[39]
Vancomycin= (V‐linked) fMLFK	**C2(B)**	0 spacers	Lysine residue of fMLFK	—
Vancomycin= (N‐linked) fMLFK	**C3(B)**	0 spacers	Lysine residue of fMLFK	—
Vancomycin= (C‐linked) fMLFG	**C4(B)**	0 spacer	Amine at the N‐terminal of vancomycin	[39]
	**C5(B)**	3 spacers		
	**C6(B)**	6 spacers		
Vancomycin= (N‐linked) fMLFG	**C7(B)**	0 spacer	Amine on vancosamine residue of vancomycin	[39]
	**C8(B)**	3 spacers		
	**C9(B)**	6 spacers		
Vancomycin= (V‐linked) fMLFG	**C10(B)**	0 spacer	Amine at the N‐terminal of vancomycin	[39]
	**C11(B)**	3 spacers		
	**C12(B)**	6 spacers		

The treatments include single staining of each bacterium population as controls to adjust for compensation during flow cytometry. All samples were acquired using BD LSRFortessa X‐20 cell analysers (BD Biosciences, USA) at FlowCore in Monash University, Clayton, Australia. BODIPY (excitation: 503 nm, emission: 512 nm) was measured in blue laser channel at 488 nm using detector B530 which has a filter range 530/30. Additionally, propidium iodide (PI, excitation: 535 nm, emission: 615 nm) was used as a viability stain measured in blue laser channel at 488 nm using detector B710 which has a filter range 710/50. Samples of 3000–10,000 events were acquired, saved as FCS 3.0 files and analysed in FlowJo v10.8.1. Populations were gated for viable single cells (Figures S1, S15−S17), and the binding levels were quantified using geometric mean fluorescence intensity (gMFI). gMFI values were plotted using the GraphPad Prism v10.4.1 software.

## Funding

This study was supported by the National Health and Medical Research Council (APP2003325) and Australian Research Council (CE200100012).

## Conflicts of Interest

The authors declare no conflicts of interest.

## Supporting information

Supplementary Material

## Data Availability

The data that support the findings of this study are available from the corresponding author upon reasonable request.
