# Supplementary material for: Assessing Vancomycin‐Formyl Peptide Conjugate Binding to the Surface of Resistant Staphylococcus aureus via Flow Cytometry
Source: Chembiochem. 2026 Aug 5;27(15):e70495. doi: 10.1002/cbic.70495 (PMC13441204; doi:10.1002/cbic.70495)
Supplement: Supplementary file 1 [file CBIC-27-e70495-s001.pdf]

# Supporting Information

# Assessing Vancomycin-Formyl Peptide Conjugate Binding to the Surface of Resistant *Staphylococcus aureus* via Flow Cytometry

Winfrey P. Y. Hoo,[a,b] Jemma Gullick,[a,b] Ryan F. Leung,[a] Alex J. Fulcher,[c] Rachel W. Cass,[a,b] Julien Tailhades,[a,b] Anton Y. Peleg,[e-g] Jennifer A. E. Payne,*[a,b,h] Meredith O'Keeffe,*[d] Max J. Cryle*[a,b,f]

[a] Infection and Immunity Program, Department of Biochemistry and Molecular Biology, Monash Biomedicine Discovery Institute, Monash University, Clayton, Victoria, Australia

[b] ARC Centre of Excellence for Innovations in Peptide and Protein Science, Clayton, Victoria, Australia

[c] Monash Micro Imaging, Monash University, Clayton, Victoria, Australia

[d] Immunity Program, Department of Biochemistry and Molecular Biology, Monash Biomedicine Discovery Institute, Monash University, Clayton, Victoria, Australia

[e] Infection Program, Department of Microbiology, Monash Biomedicine Discovery Institute, Monash University, Clayton, Victoria, Australia

[f] Centre to Impact AMR, Monash University, Clayton, Victoria, Australia

[g] Department of Infectious Diseases, Alfred Health and School of Translational Medicine, Monash University, Melbourne, Victoria, Australia

[h] Curiosity Factory Ltd, Surry Hills, NSW, Australia

* Max J. Cryle
Infection and Immunity Program, Department of Biochemistry and Molecular Biology, Monash Biomedicine Discovery Institute, Monash University, Clayton, Victoria, Australia
Email: max.cryle@monash.edu

* Meredith O'Keeffe
Immunity Program, Department of Biochemistry and Molecular Biology, Monash Biomedicine Discovery Institute, Monash University, Clayton, Victoria, Australia
Email: meredith.okeeffe@monash.edu

* Jennifer A. E. Payne
Curiosity Factory Ltd, Surry Hills, NSW, Australia
Email: ja2payne@gmail.com

# Table of Contents


1. **Supplementary Table S1**. Minimum inhibitory concentration (MIC) of unlabelled- and labelled-compounds: fMLFK, vancomycin, and fMLFK conjugated vancomycin against S. aureus clinical isolates: MRSA and VISA. BODIPY® dye was used for the respective labelling of compounds. S. aureus ATCC 29213 and E. coli ATCC 25922 were used as quality control strains. MIC assay was performed for 24 h in biological replicates (n=3 or 4); concentrations were reported in μM.

2. **Supplementary Figure S1.** Flow cytometry gating strategy to measure the binding of BODIPY®-labelled vancomycin and vancomycin=fMLFK conjugate (C-linked).

3. **Supplementary Figure S2.** Binding of vancomycin=fMLFK and vancomycin to *E. coli*.

4. **Supplementary Figure S3.** Concentration gradient showing the binding of BODIPY®-labelled: vancomycin **(B2)**, vancomycin=(C-linked)-fMLFK (**C1(B)**), vancomycin=(V-linked)-fMLFK **(C2(B)**) and vancomycin=(N-linked)-fMLFK **(C3(B)**) to resistant *S. aureus* isolates.

5. **Supplementary Figure S4.** Characterisation of vancomycin=(V-linked)-fMLFK. Structure, and LC and MS profiles for **C2(B).**

6. **Supplementary Figure S5.** Characterisation of vancomycin=(N-linked)-fMLFK. Structure, and LC and MS profiles for **C3(B).**

7. **Supplementary Table S2.** Binding of BODIPY®-labelled compounds: fMLFK, vancomycin, and fPep-conjugated vancomycin to the surface S. aureus clinical isolates: MRSA and VISA. Binding was quantified by flow cytometry in biological replicates (n=3 or 6). BODIPY® fluorescence was then reported as geometric mean fluorescence intensity (gMFI) value.

8. **Supplementary Figure S6.** Characterisation of vancomycin=(C-linked)-fMLFG with 0 PEG spacer. Structure, and LC and MS profiles for **C4(B).**

9. **Supplementary Figure S7.** Characterisation of vancomycin=(C-linked)-fMLFG with 3 PEG spacers. Structure, and LC and MS profiles for **C5(B).**

10. **Supplementary Figure S8.** Characterisation of vancomycin=(C-linked)-fMLFG with 6 PEG spacers. Structure, and LC and MS profiles for **C6(B).**

11. **Supplementary Figure S9.** Characterisation of vancomycin=(N-linked)-fMLFG with 0 PEG spacer. Structure, and LC and MS profiles for **C7(B).**

12. **Supplementary Figure S10.** Characterisation of vancomycin=(N-linked)-fMLFG with 3 PEG spacers. Structure, and LC and MS profiles for **C8(B).**

13. **Supplementary Figure S11.** Characterisation of vancomycin=(N-linked)-fMLFG with 6 PEG spacers. Structure, and LC and MS profiles for **C9(B).**

14. **Supplementary Figure S12.** Characterisation of vancomycin=(V-linked)-fMLFG with 0 PEG spacer. Structure, and LC and MS profiles for **C10(B).**

15. **Supplementary Figure S13.** Characterisation of vancomycin=(V-linked)-fMLFG with 3 PEG spacers. Structure, and LC and MS profiles for **C11(B).**

16. **Supplementary Figure S14.** Characterisation of vancomycin=(V-linked)-fMLFG with 6 PEG spacers. Structure, and LC and MS profiles for **C12(B).**

17. **Supplementary Figure S15.** Flow cytometry gating strategy to measure the binding of BODIPY®-labelled vancomycin and vancomycin=fMLFG conjugates in MRSA A8090.

18. **Supplementary Figure S16.** Flow cytometry gating strategy to measure the binding of BODIPY®-labelled vancomycin and vancomycin=fMLFG conjugates in VISA A8094.

19. **Supplementary Figure S17.** Flow cytometry gating strategy to measure the binding of BODIPY®-labelled vancomycin and vancomycin=fMLFG conjugates in E. coli ATCC 25922.

20. **References**

**Supplementary Table S1.** Minimum inhibitory concentration (MIC) of unlabelled- and labelled-compounds: fMLFK, vancomycin, and fMLFK conjugated vancomycin against *S. aureus* clinical isolates: MRSA and VISA. BODIPY® dye was used for the respective labelling of compounds. *S. aureus* ATCC 29213 and *E. coli* ATCC 25922 were used as quality control strains. MIC assay was performed for 24 h in biological replicates (n=3 or 4); concentrations were reported in μM.

| **MIC assay (μM)** | | **Dye** | ***S. aureus*** **ATCC 29213** | **MRSA A8090** | **VISA A8094** | ***E. coli*** **ATCC 25922** |
|---|---|---|---|---|---|---|
| **fMLFK** | **FP1** | - | >32 | >32 | >32 | >32 |
| **Vancomycin‡** | **VAN** | - | 0.0625 - 0.125 | 0.0625 - 0.125 | 0.5 - 1 | >32 |
| | **B2** | BODIPY® | 0.5 | 0.5 | 4 - >32 | >32 |
| **VAN=fMLFK** | **C1** | - | 1 - 2 | 1 - 2 | ≥16 | >32 |
| | **C1(B)** | BODIPY® | 4 | 4 | >32 | >32 |

‡ Reference MIC of 1 μg/mL (equivalent to 0.67 μM) for MRSA A8090 and 8 μg/mL (equivalent to 5.3 μM) for VISA A8094.[1, 2]

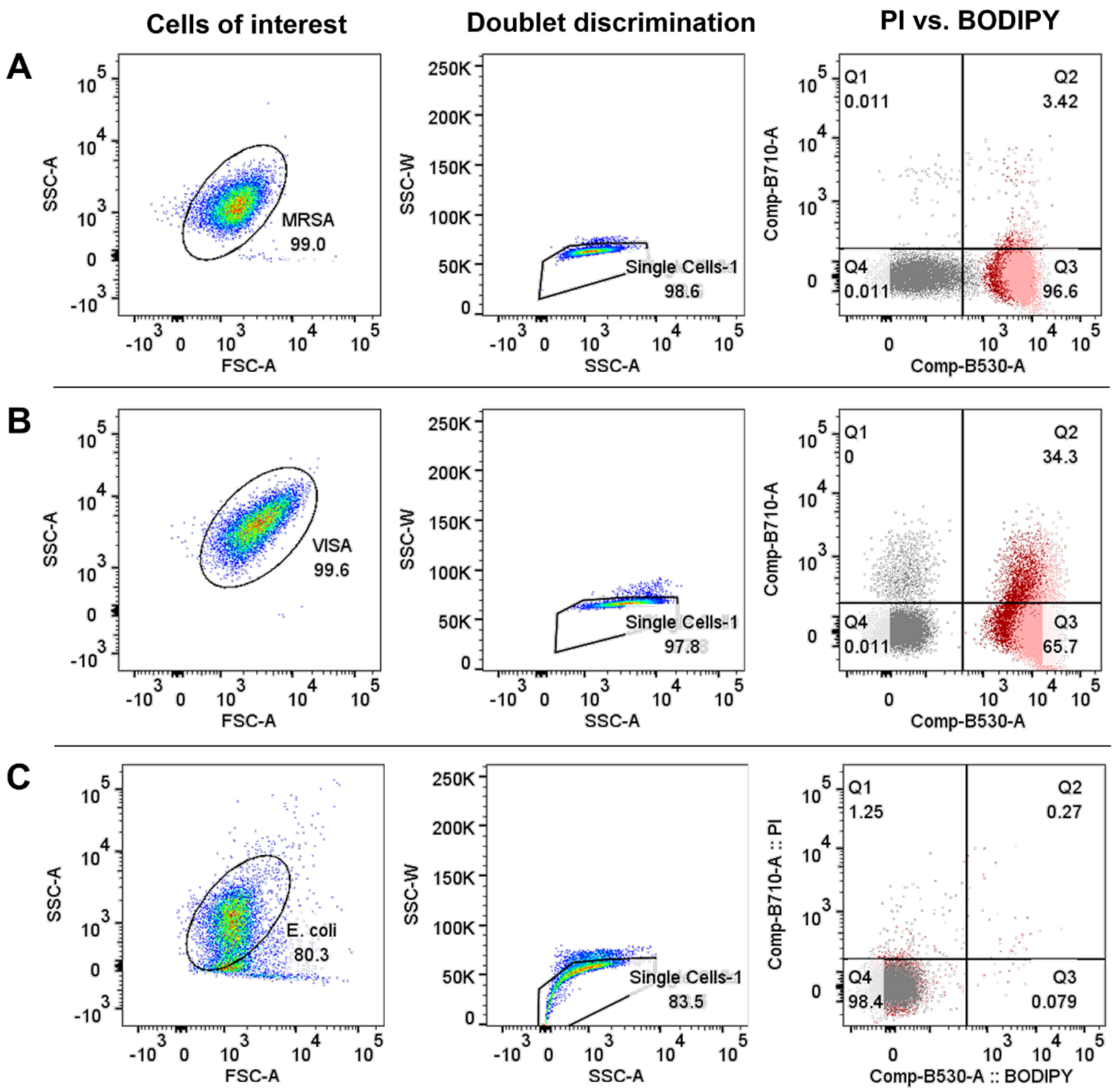


**Supplementary Figure S1.** Flow cytometry gating strategy to measure the binding of BODIPY®-labelled vancomycin and vancomycin=fMLFK conjugate (C-linked). Clinical isolates of MRSA A8090 (**A**) and VISA A8094 (**B**) were individually treated with BODIPY®-labelled: fMLFK (grey); VAN (pink); and VAN=fMLFK (dark red). *E. coli* ATCC 25922 (**C**) was used as a control for negative interactions between targeting element vancomycin and surface of *E. coli*, while fMLFK was used as a control for negative interaction between fPep as a chemoattractant and bacterial cell surface. After washing to remove excess unincorporated compounds, bacteria were resuspended and added with propidium iodide (PI) as a viability stain prior to sample acquisition on flow cytometer. Bacteria were first gated based on size (FSC-A) and granularity (SSC-A), followed by exclusion of doublets using SSC-W against SSC-A plot. In PI vs. BODIPY dot plots, viable cells would be observed in Q3 and Q4 while non-viable cells (based on positive signal from PI staining) would be observed in Q1 and Q2. Cells were further analysed for BODIPY® fluorescence where population sitting in Q2 and Q3 would indicate binding of compounds to bacteria while population sitting in Q1 and Q4 would indicate no interaction between compounds and bacteria. Populations of MRSA A8090 and VISA A8094 showed no interaction with fMLFK but showed positive interactions with VAN and VAN=fMLFK. Meanwhile, population of *E. coli* ATCC 25922 showed no interactions with fMLFK, VAN nor VAN=fMLFK. BODIPY® fluorescence was then reported as geometric mean fluorescence intensity (gMFI) value to compare the interaction of the compounds to cell surface of *S. aureus* and *E. coli*.

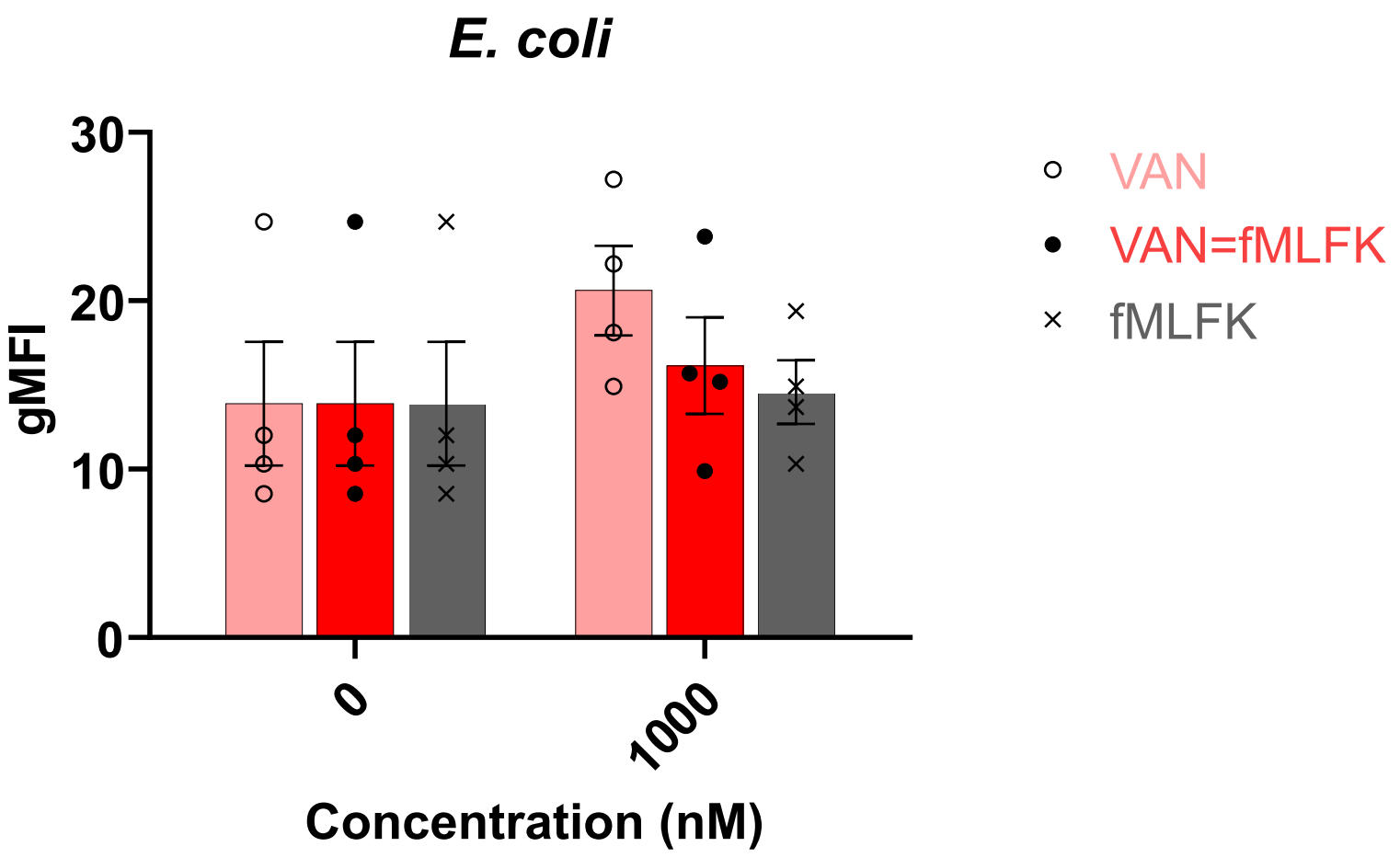


**Supplementary Figure S2.** Binding of vancomycin=fMLFK and vancomycin to *E. coli*. Flow cytometry was performed to measure the binding levels of BODIPY®-labelled free vancomycin (pink salmon) and fMLFK conjugated vancomycin (dark red). Treatments were performed at concentrations of 0 nM and 1000 nM against *E. coli* ATCC 25922. fMLFK (grey) was used as a negative control in binding to cell surface of bacteria. Flow cytometry was performed in biological replicates (n=3 or 4); geometric mean fluorescence intensity is gMFI; error bars are SEM.

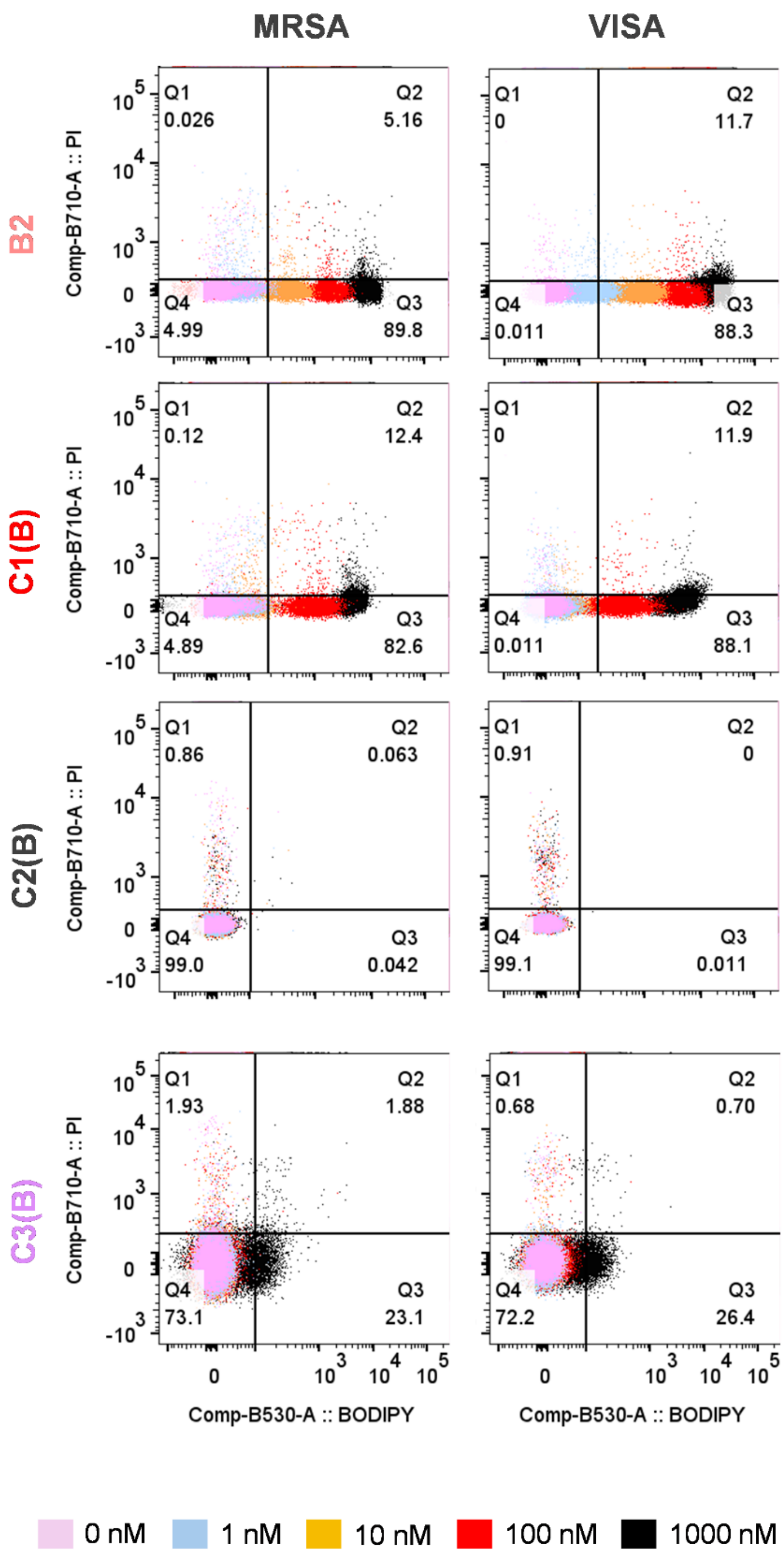


**Supplementary Figure S3.** Concentration gradient showing the binding of BODIPY®-labelled: vancomycin (**B2**), vancomycin=(C-linked)-fMLFK (**C1(B)**), vancomycin=(V-linked)-fMLFK (**C2(B)**) and vancomycin=(N-linked)-fMLFK (**C3(B)**) to resistant *S. aureus* isolates quantified by flow cytometry. Treatments were performed at following concentrations: 0 nM (light purple), 1 nM (light blue), 10 nM (orange), 100 nM (dark red), and 1000 nM (black). Increase in binding was indicated by the increase in BODIPY® fluorescence as the concentrations of **B2** and **C1(B)** increase. Meanwhile, **C2(B)** and **C3(B)** showed no binding across all concentration range. Data is plotted as BODIPY® fluorescence versus propidium iodide (PI) uptake.

## C2(B)

Chemical Formula: $C_{118}H_{146}BCl_2F_2N_{21}O_{32}S$
Exact Mass: 2519.96
Molecular Weight: 2522.35

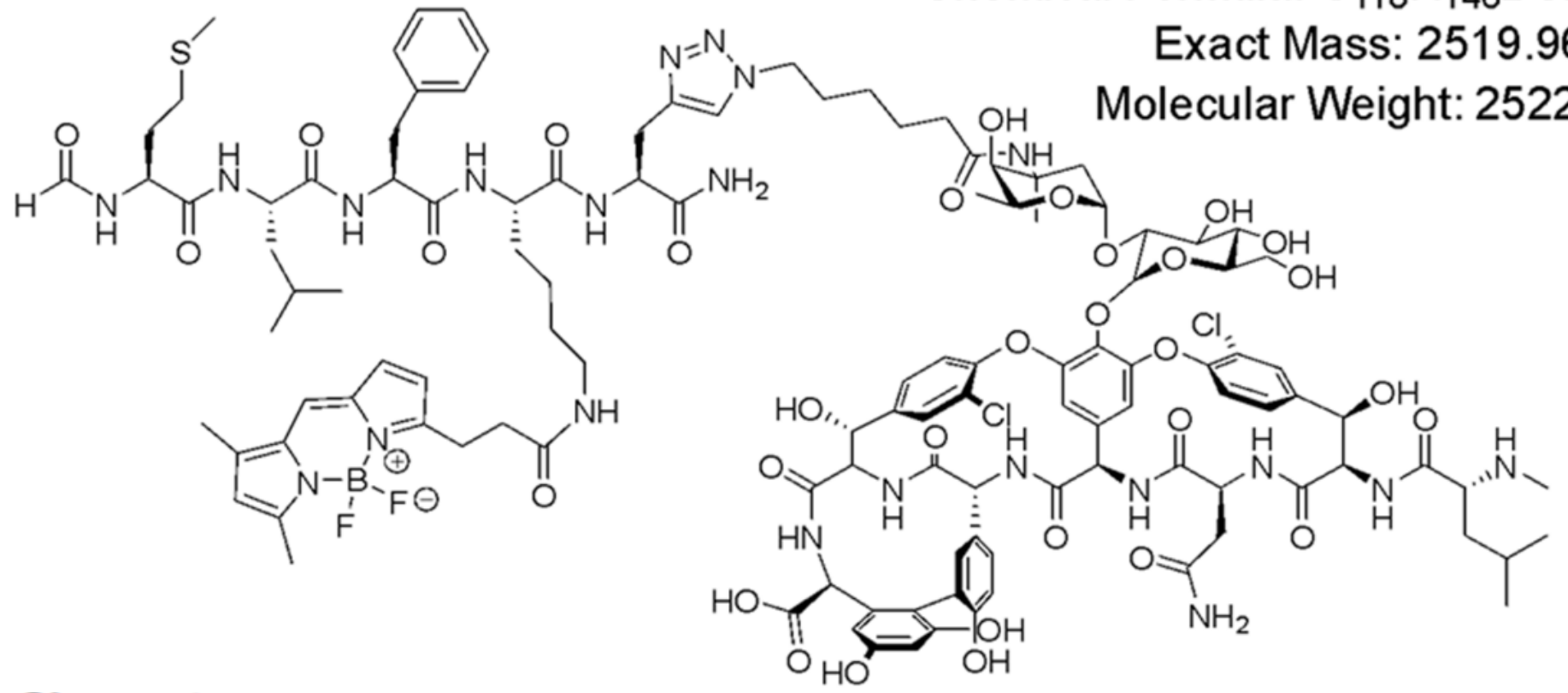

**<Chromatogram>**

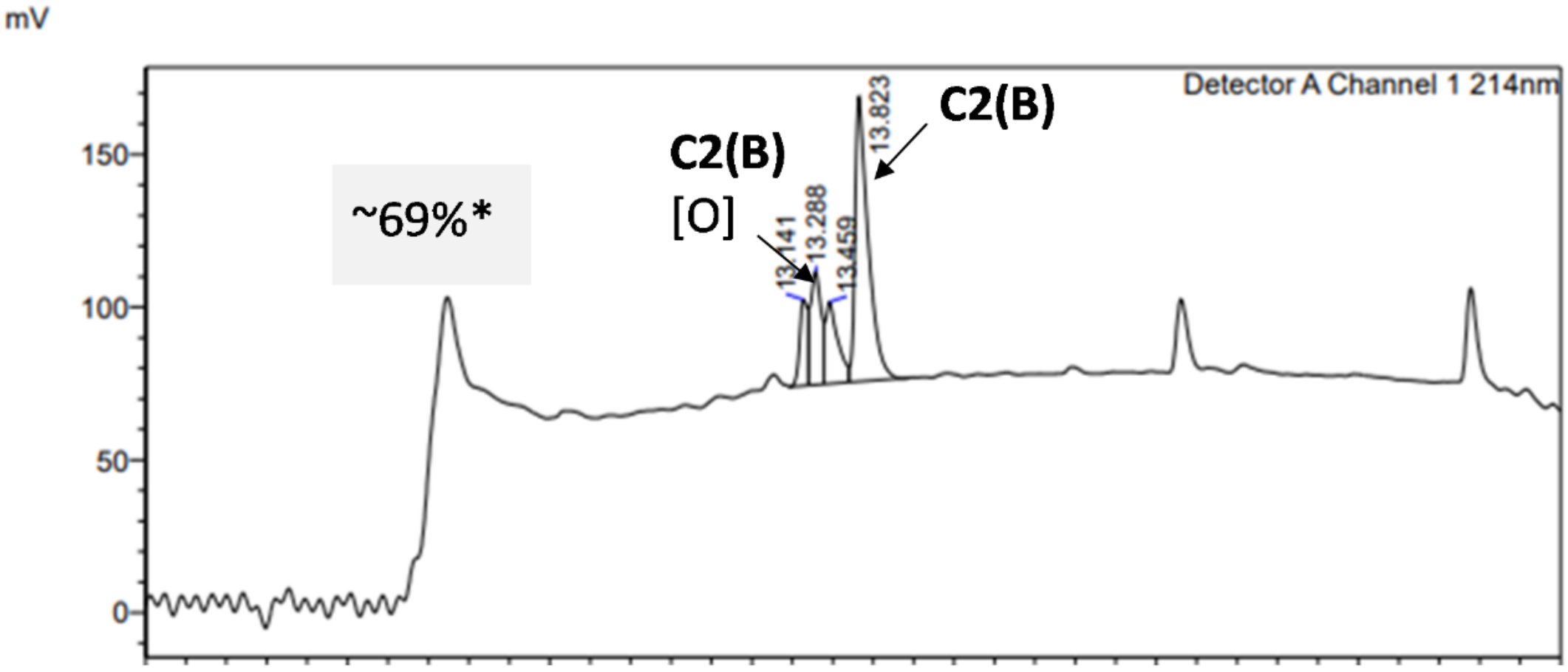


## <Spectrum>

Line#:1 R.Time:13.933(Scan#:837)
MassPeaks:2044
RawMode:Single 13.933(837) BasePeak:1262.1(1184325)
BG Mode:None Segment 1 - Event 1

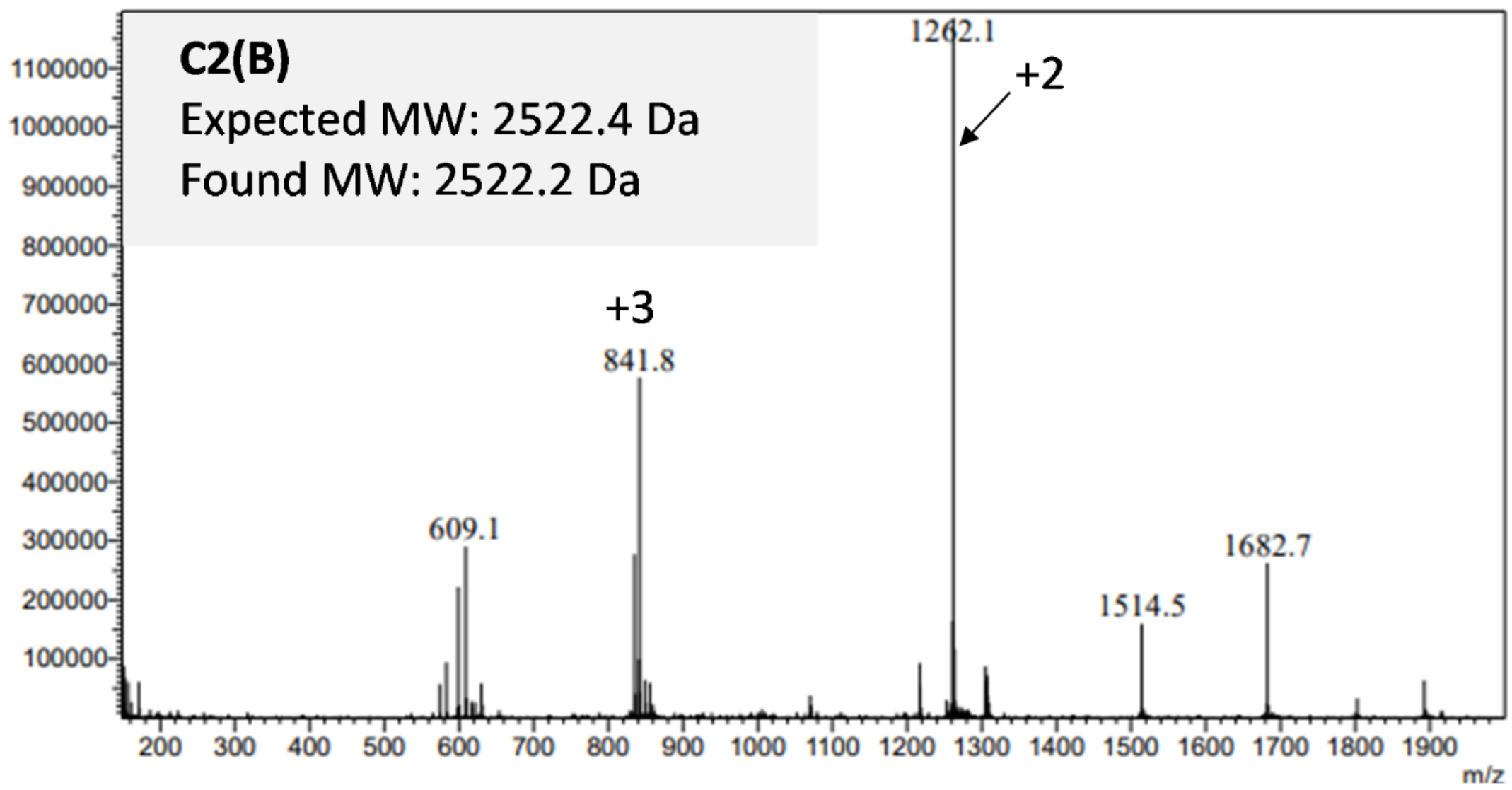


**Supplementary Figure S4.** Characterisation of vancomycin=(V-linked)-fMLFK. Structure, and LC and MS profiles for **C2(B)**. [O] – oxidised methionine species [M+16 Da].

Chemical Formula: $C_{118}H_{146}BCl_2F_2N_{21}O_{32}S$

Exact Mass: 2519.96

Molecular Weight: 2522.35

**C3(B)**

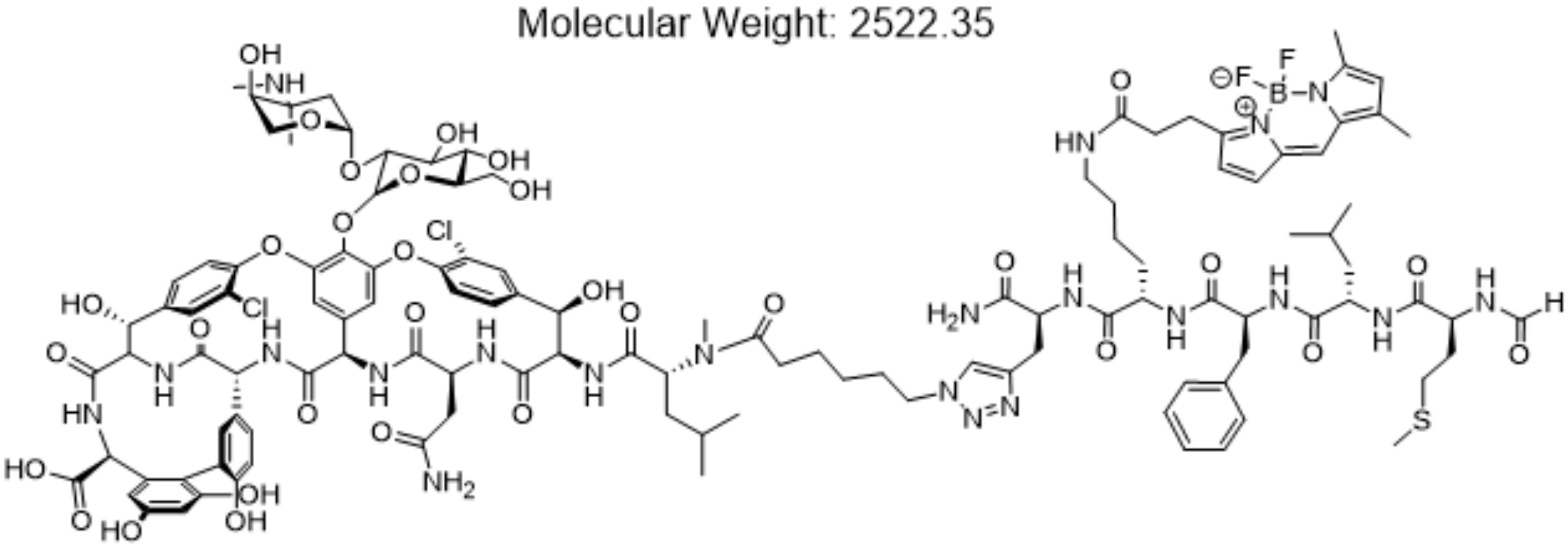

**<Chromatogram>**

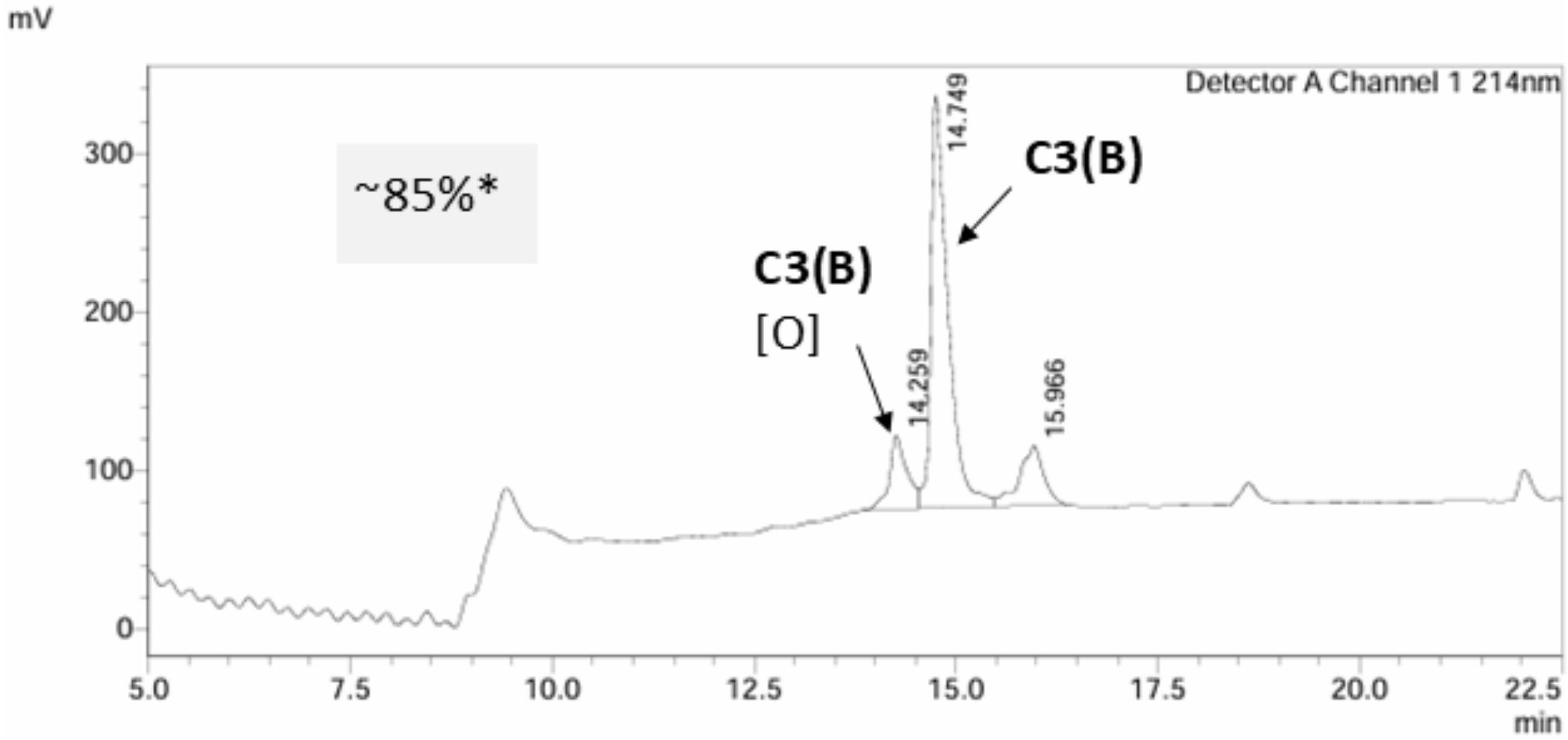


**<Spectrum>**

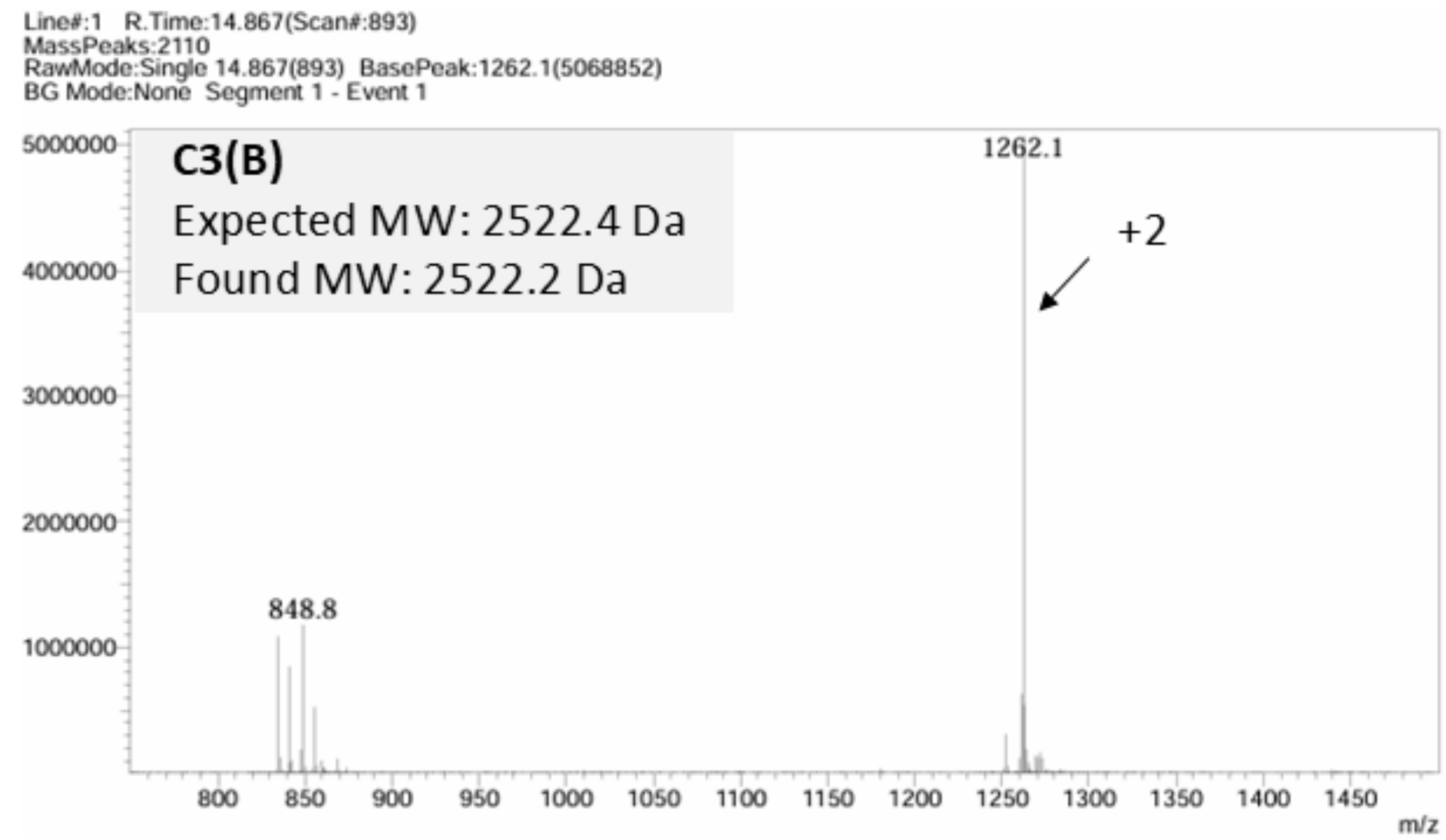


**Supplementary Figure S5.** Characterisation of vancomycin=(N-linked)-fMLFK. Structure, and LC and MS profiles for **C3(B)**. [O] – oxidised methionine species [M+16 Da].

**Supplementary Table S2.** Binding of BODIPY®-labelled compounds: fMLFK, vancomycin, and fPep-conjugated vancomycin to the surface *S. aureus* clinical isolates: MRSA and VISA. Binding was quantified by flow cytometry in biological replicates (n=3 or 6). BODIPY® fluorescence was then reported as geometric mean fluorescence intensity (gMFI) value. Purity calculation shown includes both the non-oxidized and the oxidized (O) species shown in the relevant HPLC traces.

| BODIPY® labelled compound | Abbr. | fPep | fMLFK linkage to VAN | PEG spacer | Molecular weight | Purity % | MRSA | | VISA | |
|---|---|---|---|---|---|---|---|---|---|---|
| | | | | | | | Average gMFI | Average gMFI at 100% purity | Average gMFI | Average gMFI at 100% purity |
| fMLFK | B1 | fMLFK | - | - | 933.4 | 84 | 0.012 | 0.015 | 0.010 | 0.012 |
| Vancomycin | VAN; B2 | - | - | - | 1860.7 | 65 | 2.889 | 4.445 | 3.895 | 5.993 |
| VAN=(C-linked)-fMLFK* | C1(B) | fMLFK | C | 0 | 2462.9 | 100 | 0.554 | 0.554 | 0.589 | 0.589 |
| VAN=(V-linked)-fMLFK | C2(B) | fMLFK | V | 0 | 2522.4 | 69 | 0.043 | 0.063 | 0.038 | 0.054 |
| VAN=(N-linked)-fMLFK | C3(B) | fMLFK | N | 0 | 2522.4 | 85 | 0.057 | 0.067 | 0.055 | 0.065 |
| VAN=(C-linked)-fMLFG | C4(B) | fMLFG | C | 0 | 2394.2 | 81 | 130.533 | 161.151 | 76.850 | 94.877 |
| VAN=(C-linked)-fMLFG | C5(B) | fMLFG | C | 3 | 2597.4 | 73 | 63.650 | 87.192 | 49.550 | 67.877 |
| VAN=(C-linked)-fMLFG | C6(B) | fMLFG | C | 6 | 2800.7 | 77 | 35.353 | 45.912 | 27.575 | 35.812 |
| VAN=(N-linked)-fMLFG | C7(B) | fMLFG | N | 0 | 2437.2 | 74 | 8.515 | 11.507 | 8.723 | 11.787 |
| VAN=(N-linked)-fMLFG | C8(B) | fMLFG | N | 3 | 2640.4 | 79 | 8.683 | 10.991 | 8.628 | 10.921 |
| VAN=(N-linked)-fMLFG | C9(B) | fMLFG | N | 6 | 2843.7 | 78 | 8.315 | 10.660 | 8.338 | 10.689 |
| VAN=(V-linked)-fMLFG | C10(B) | fMLFG | V | 0 | 2453.2 | 29 | 14.123 | 48.698 | 16.350 | 56.379 |
| VAN=(V-linked)-fMLFG | C11(B) | fMLFG | V | 3 | 2640.4 | 68 | 10.165 | 14.949 | 14.875 | 21.875 |
| VAN=(V-linked)-fMLFG | C12(B) | fMLFG | V | 6 | 2859.7 | 68 | 13.020 | 19.147 | 13.525 | 19.890 |

* C1(B) previously purified; see Payne at al. [3]

**C4(B)**

Chemical Formula: $C_{111}H_{134}BCl_2F_2N_{21}O_{30}S$
Exact Mass: 2391.88
Molecular Weight: 2394.18

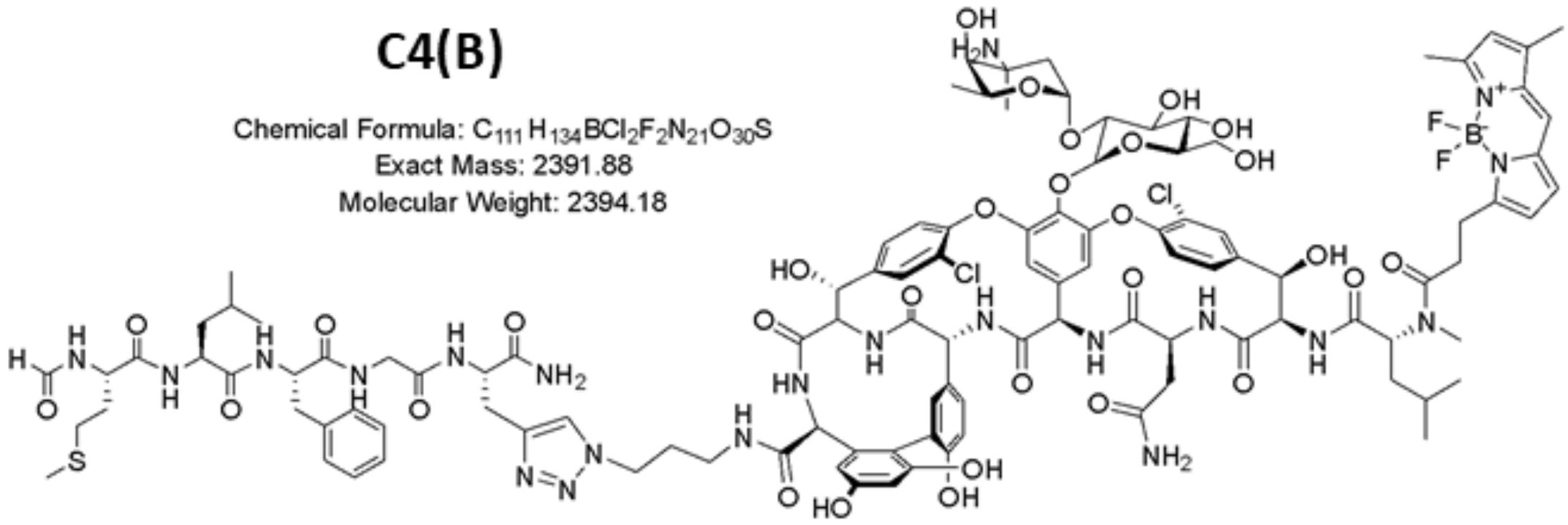

**<Chromatogram>**

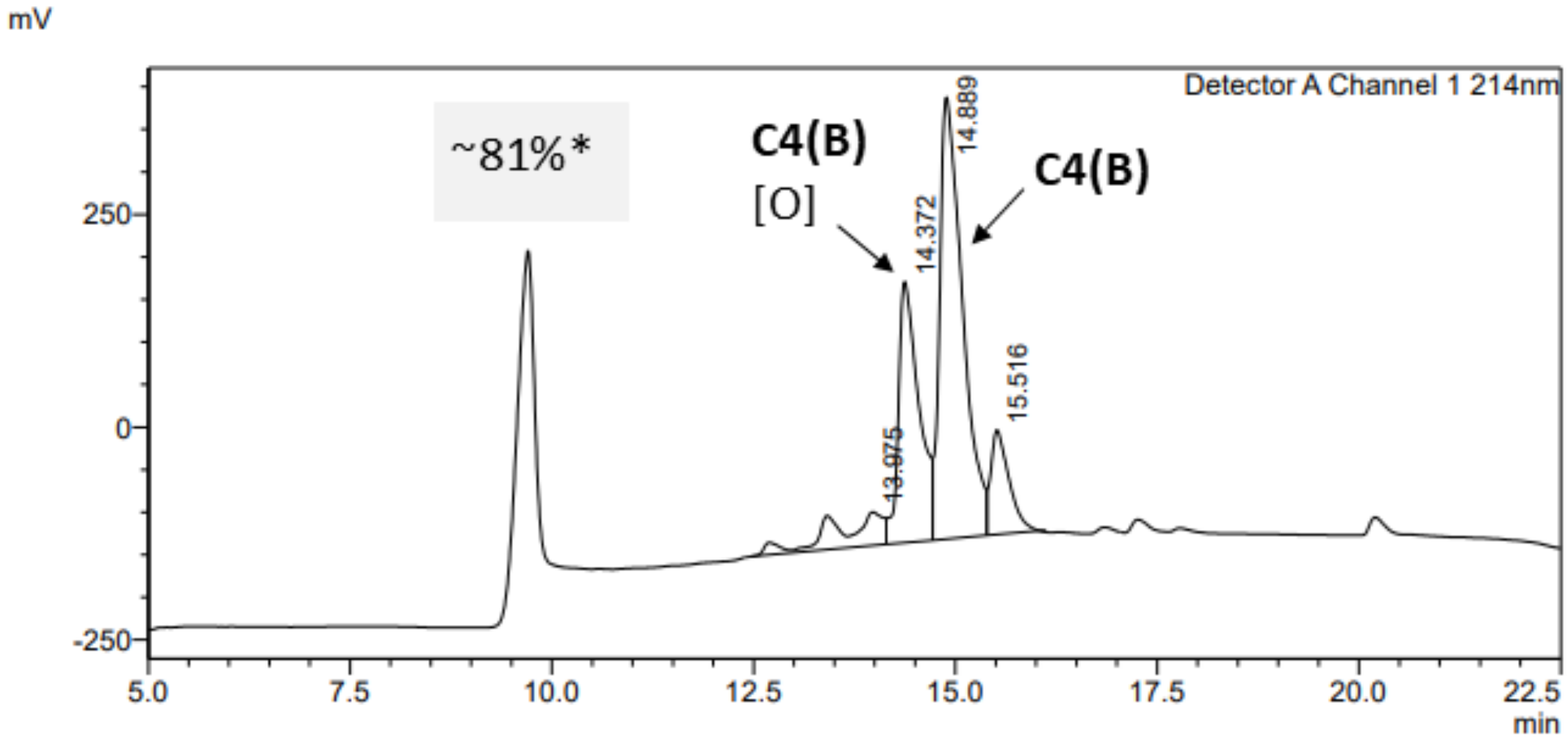


**<Spectrum>**

Line#:1 R.Time:15.000(Scan#:901)
MassPeaks:2009
RawMode:Single 15.000(901) BasePeak:792.3(3666329)
BG Mode:None Segment 1 - Event 1

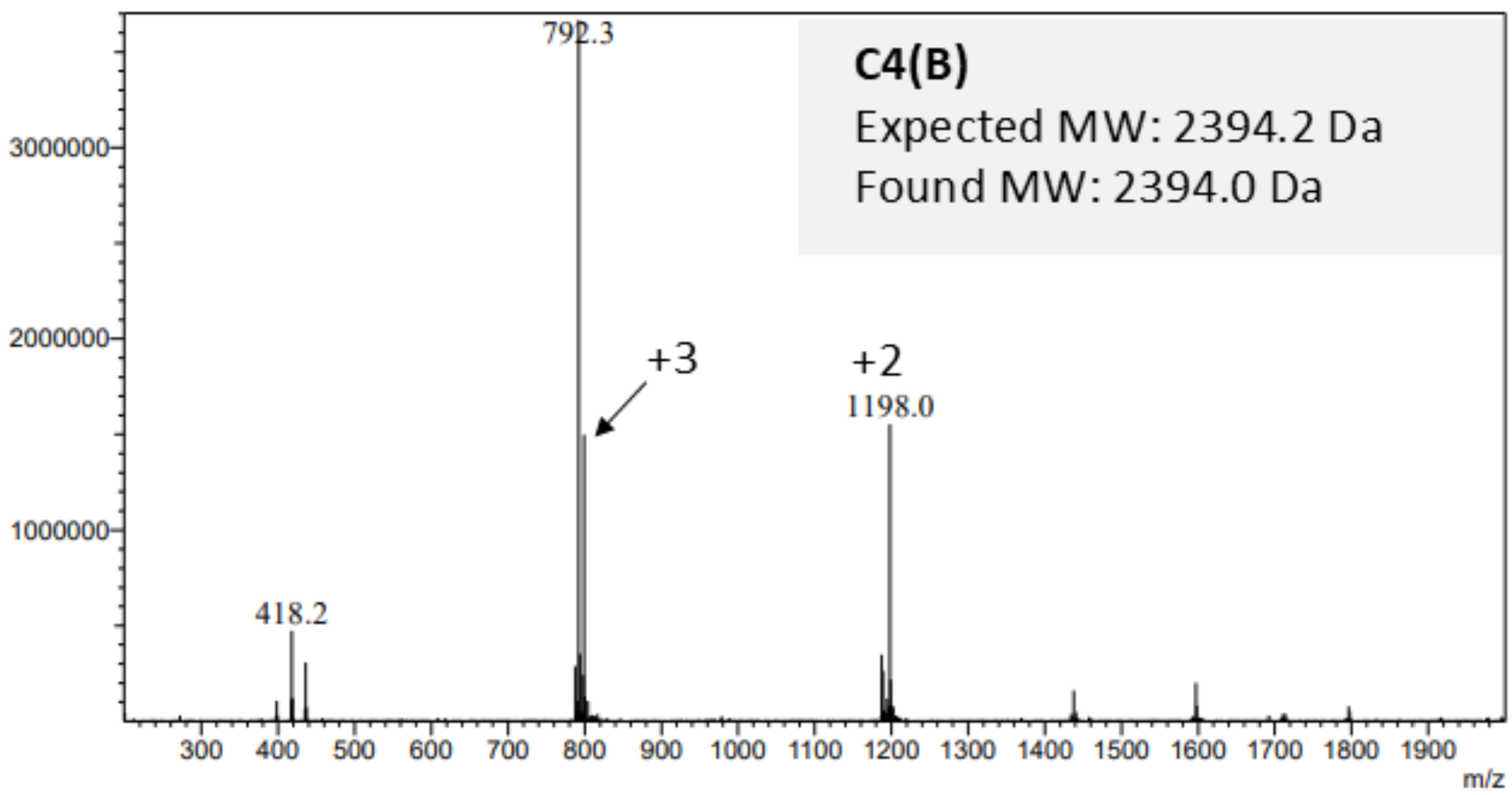


**Supplementary Figure S6.** Characterisation of vancomycin=(C-linked)-fMLFG with 0 PEG spacer. Structure, and LC and MS profiles for **C4(B)**. [O] – oxidised methionine species [M+16 Da].

## C5(B)

Chemical Formula: $C_{120}H_{151}BCl_2F_2N_{22}O_{34}S$
Exact Mass: 2594.99
Molecular Weight: 2597.41

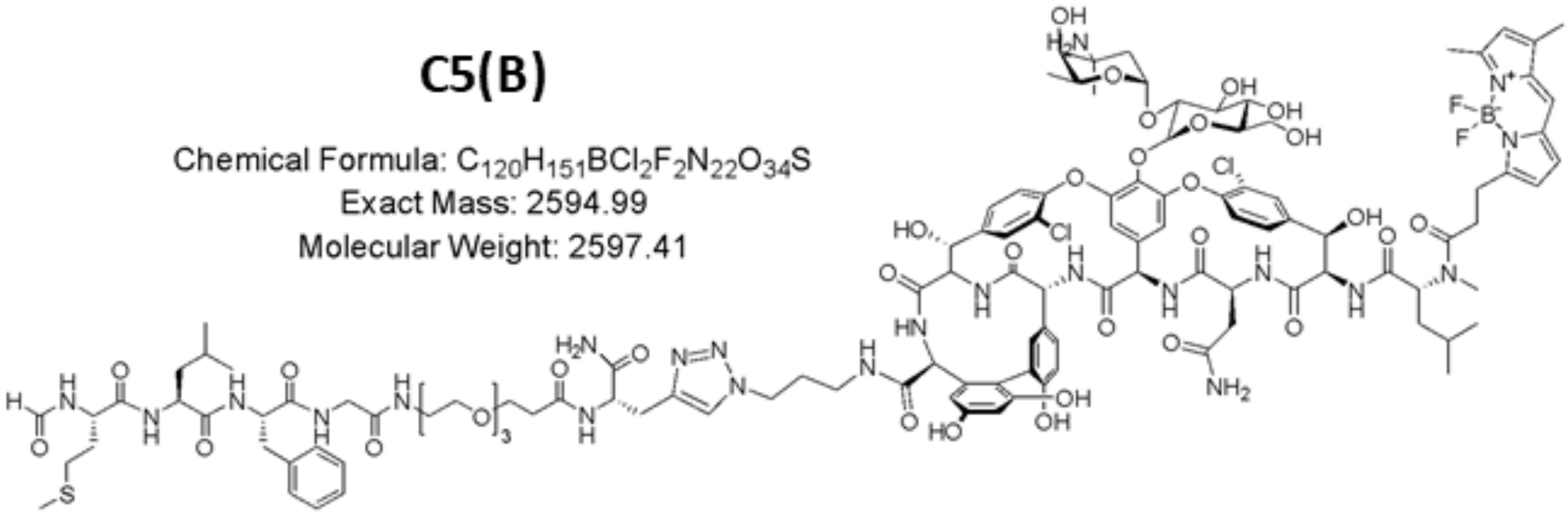

**<Chromatogram>**

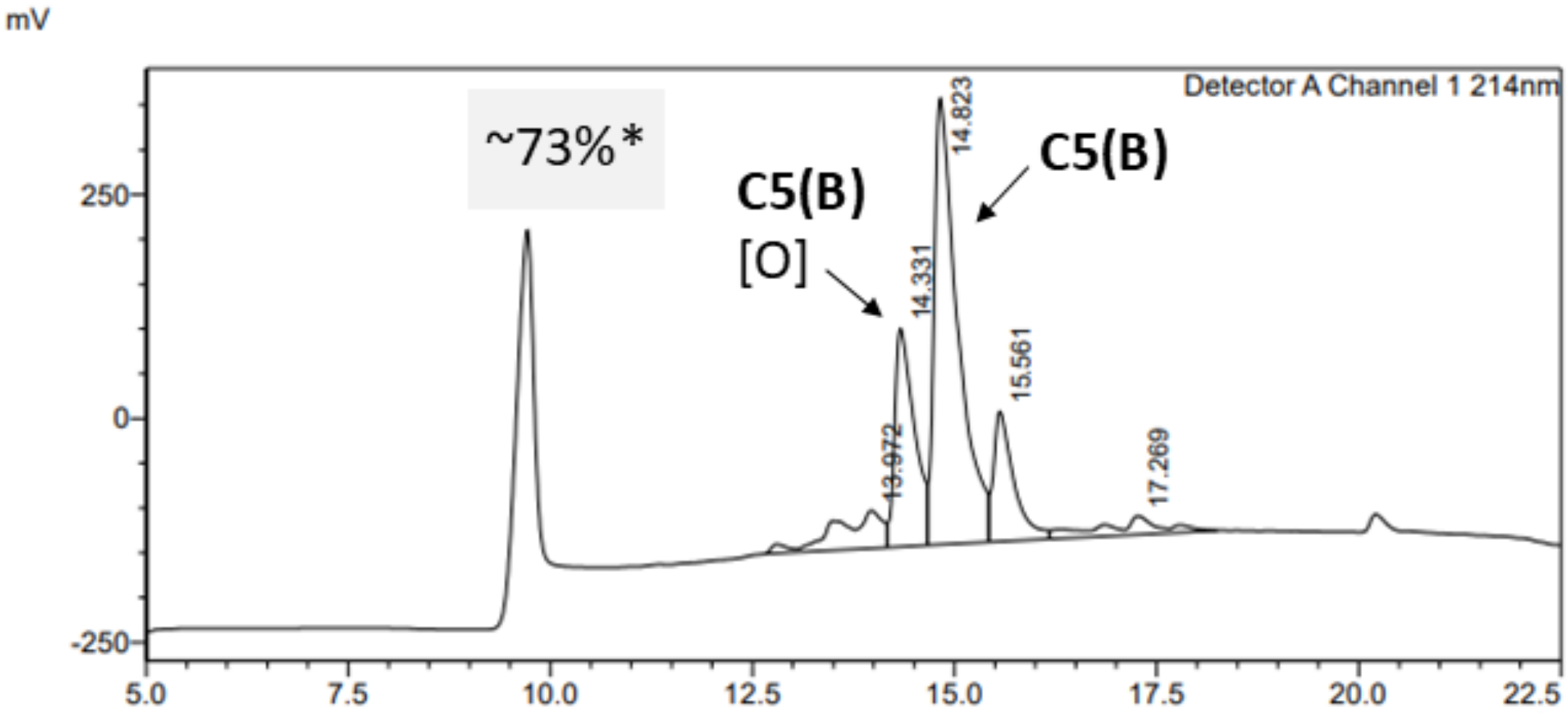


## <Spectrum>

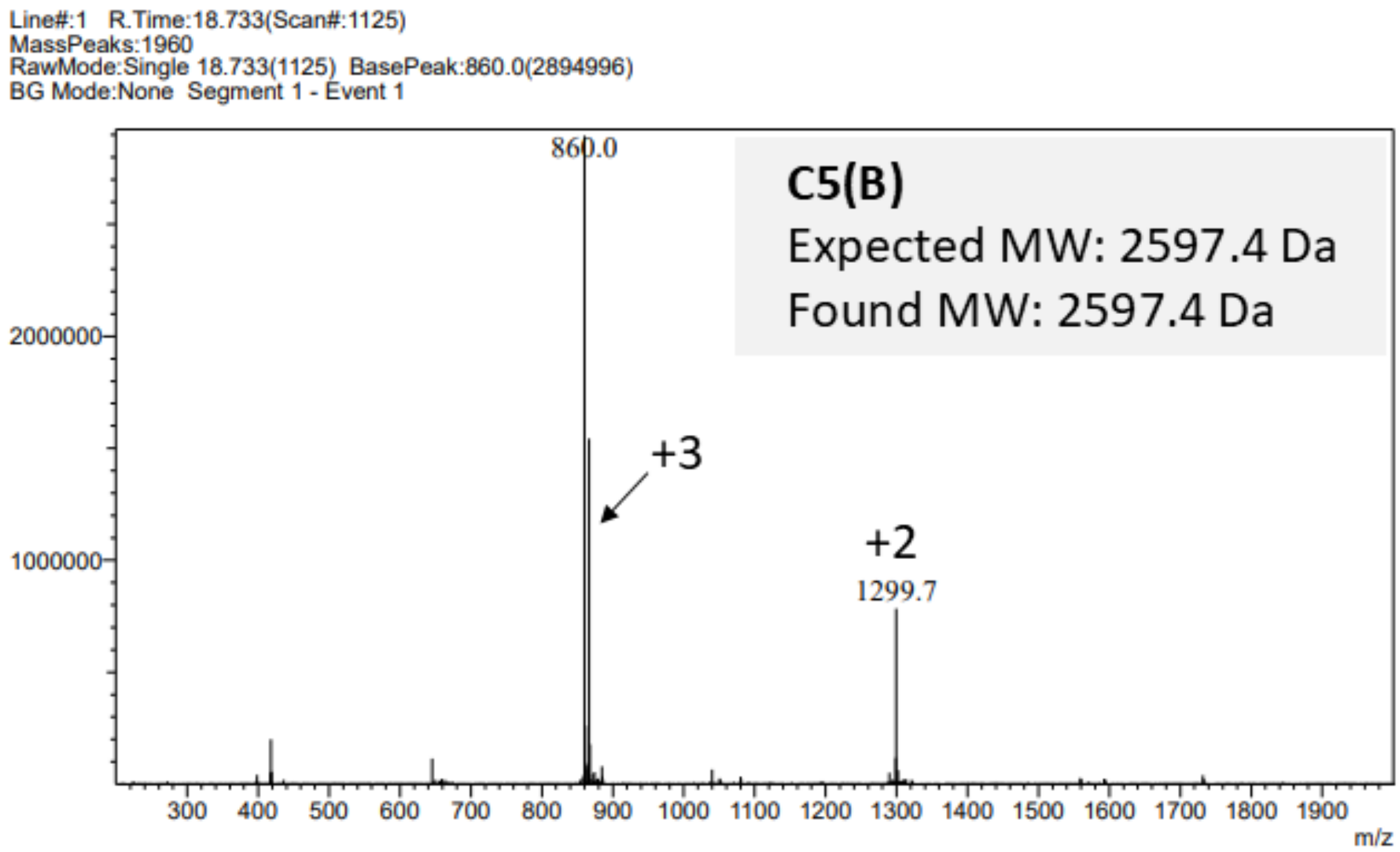


**Supplementary Figure S7.** Characterisation of vancomycin=(C-linked)-fMLFG with 3 PEG spacers. Structure, and LC and MS profiles for **C5(B)**. [O] – oxidised methionine species [M+16 Da].

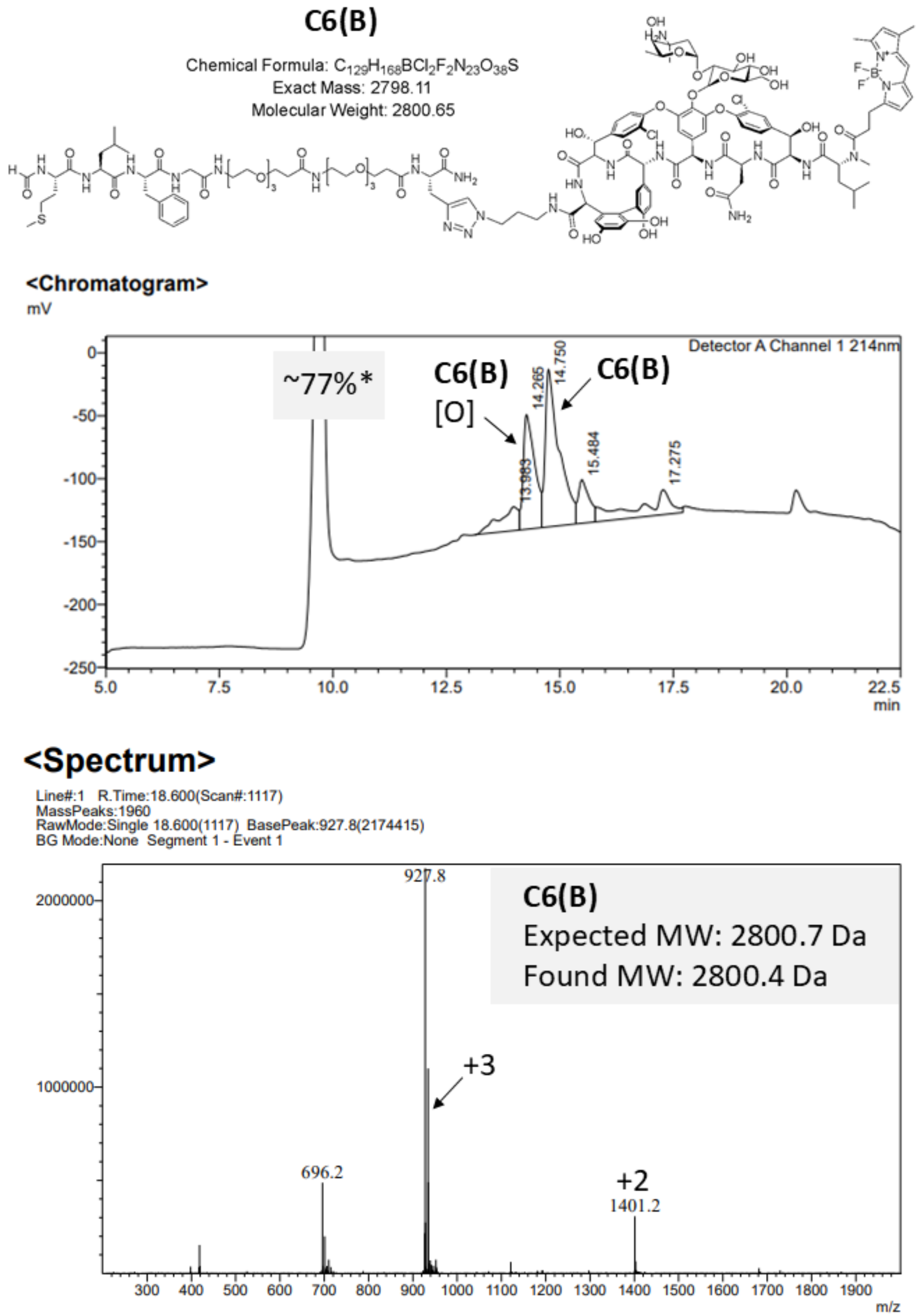


**Supplementary Figure S8.** Characterisation of vancomycin=(C-linked)-fMLFG with 6 PEG spacers. Structure, and LC and MS profiles for **C6(B)**. [O] – oxidised methionine species [M+16 Da].

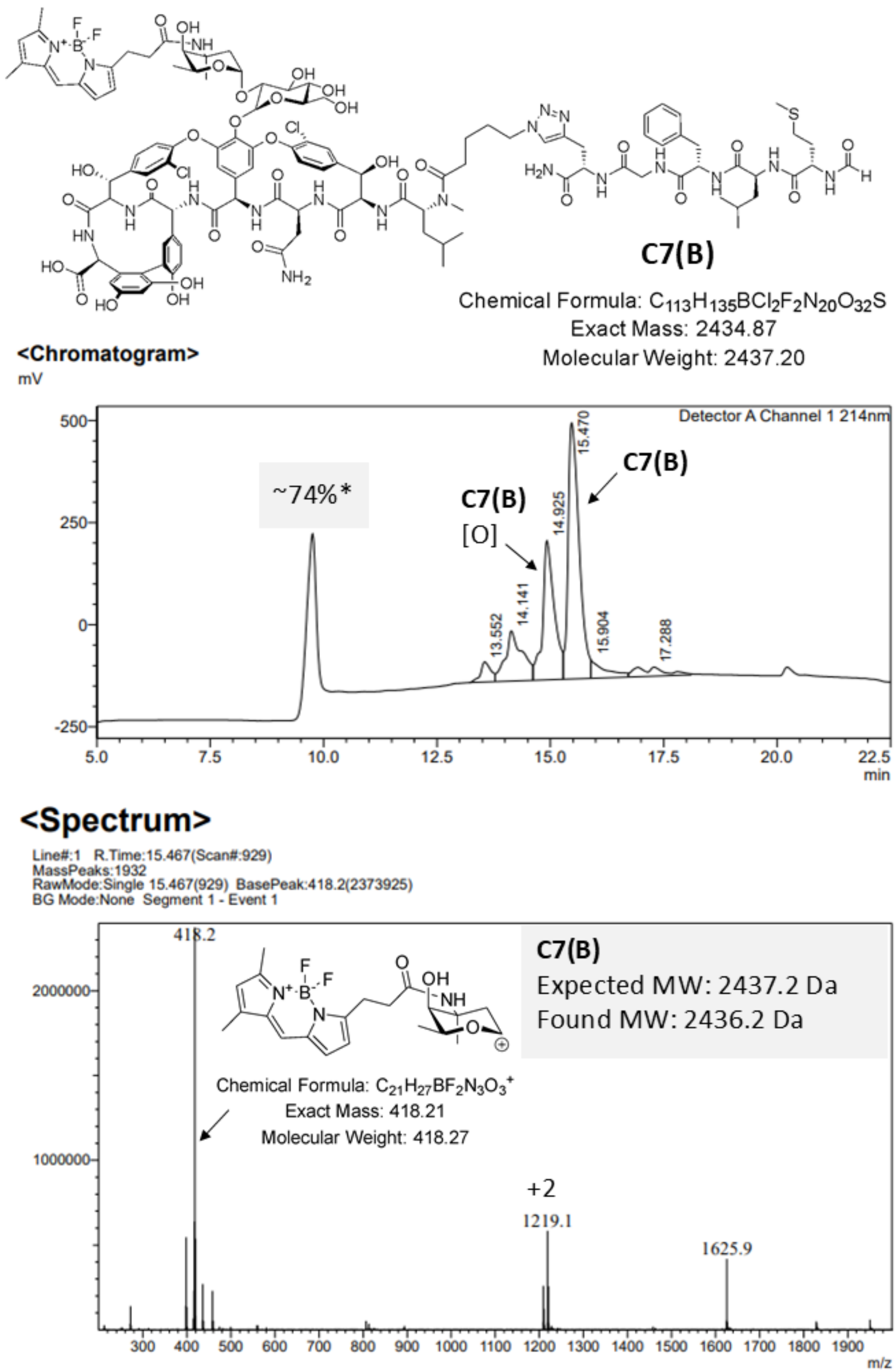


**Supplementary Figure S9.** Characterisation of vancomycin=(N-linked)-fMLFG with 0 PEG spacer. Structure, and LC and MS profiles for **C7(B)**. [O] – oxidised methionine species [M+16 Da].

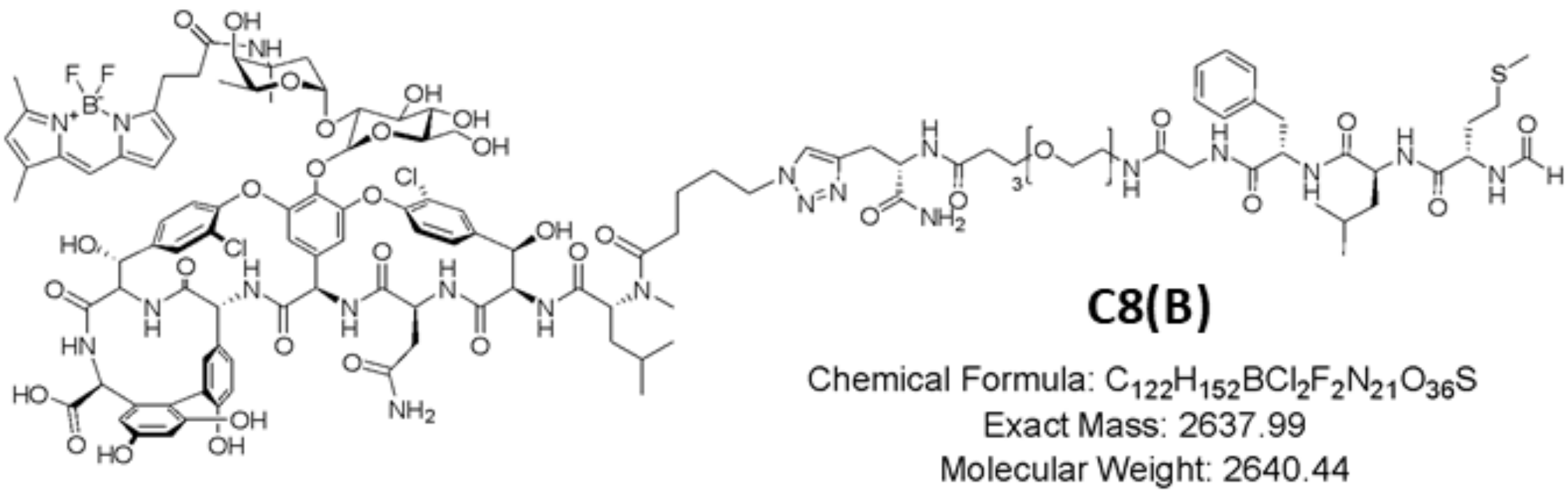

**C8(B)**

Chemical Formula: $C_{122}H_{152}BCl_2F_2N_{21}O_{36}S$
Exact Mass: 2637.99
Molecular Weight: 2640.44

**<Chromatogram>**

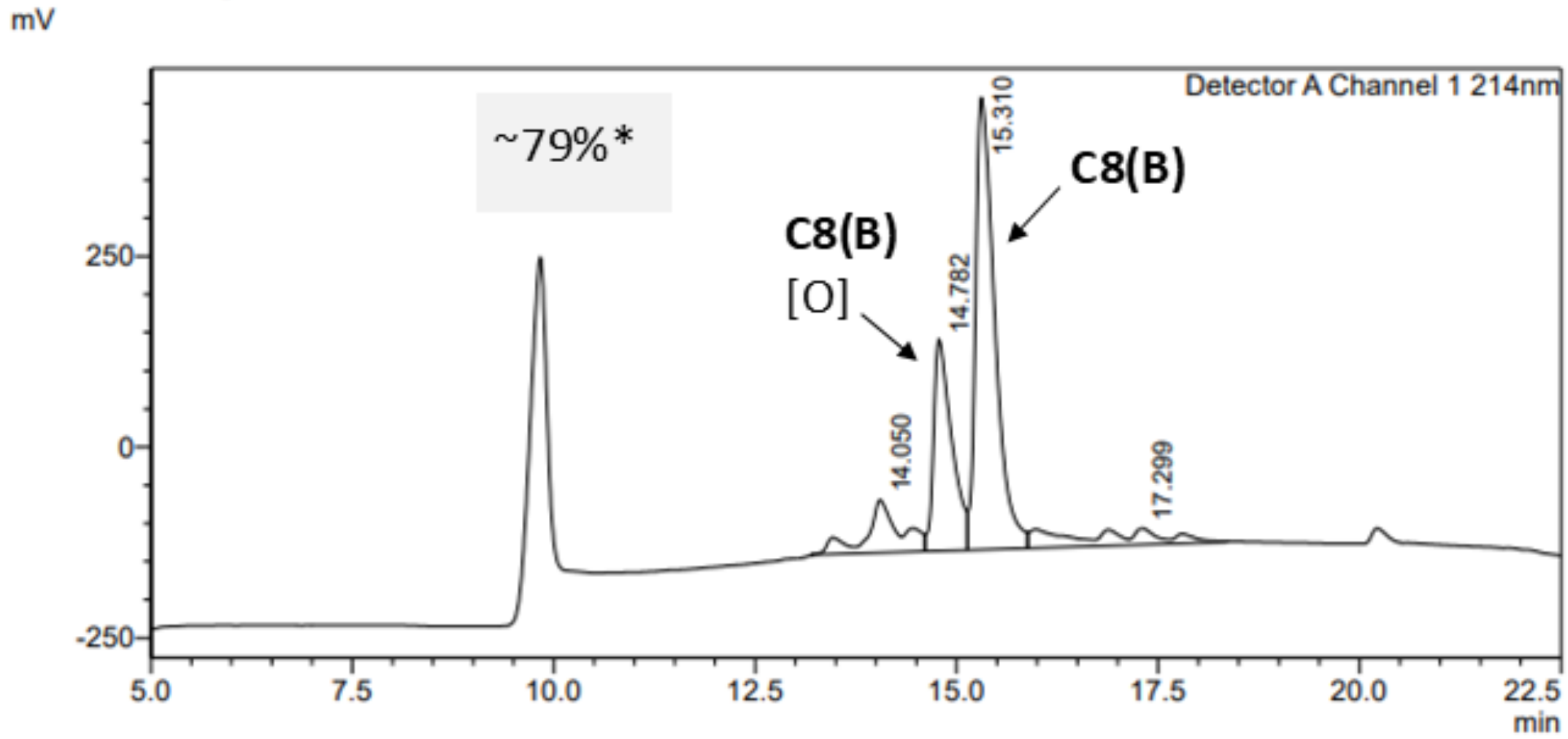


## <Spectrum>

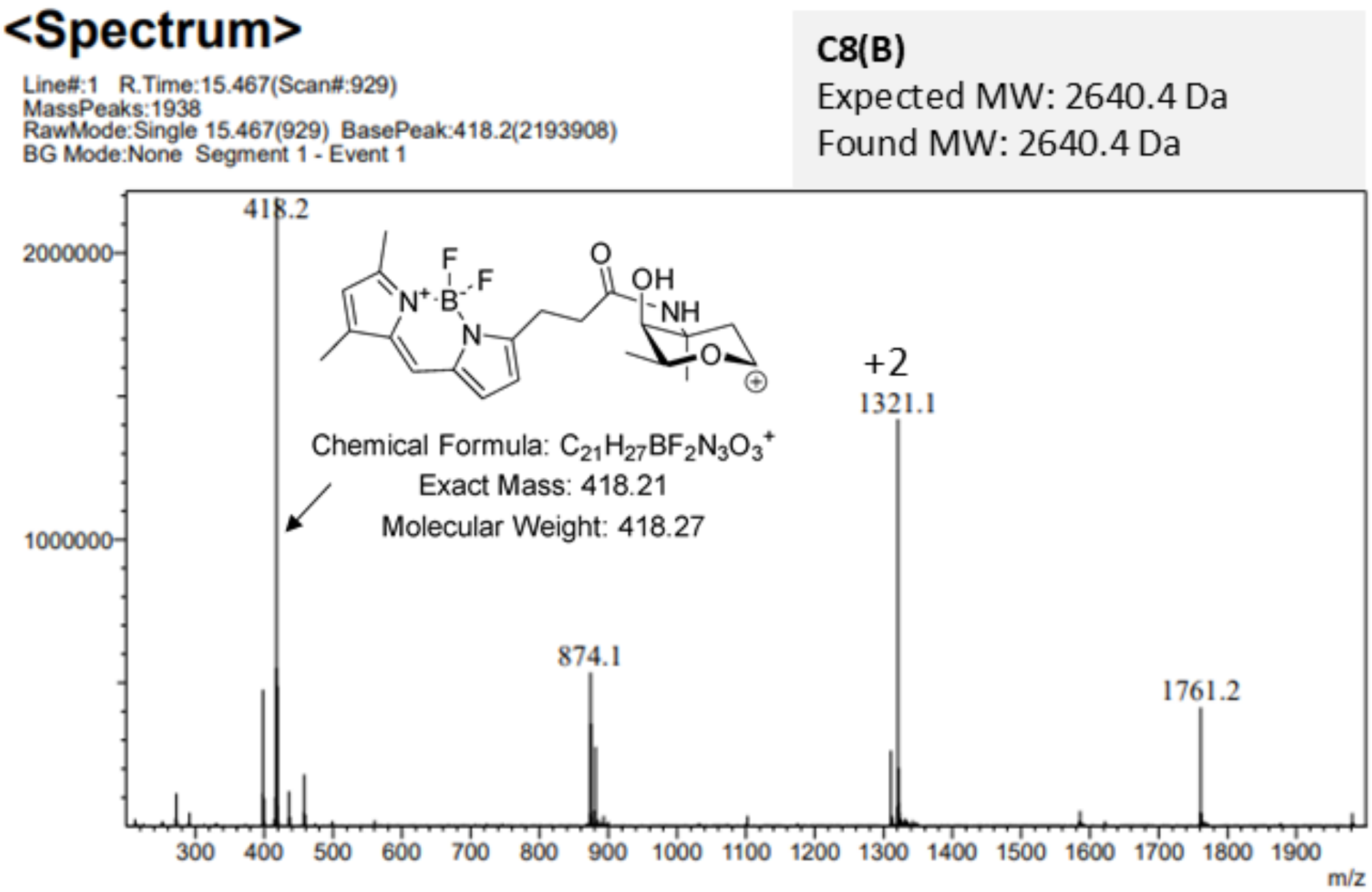


**Supplementary Figure S10.** Characterisation of vancomycin=(N-linked)-fMLFG with 3 PEG spacers. Structure, and LC and MS profiles for **C8(B)**. [O] – oxidised methionine species [M+16 Da].

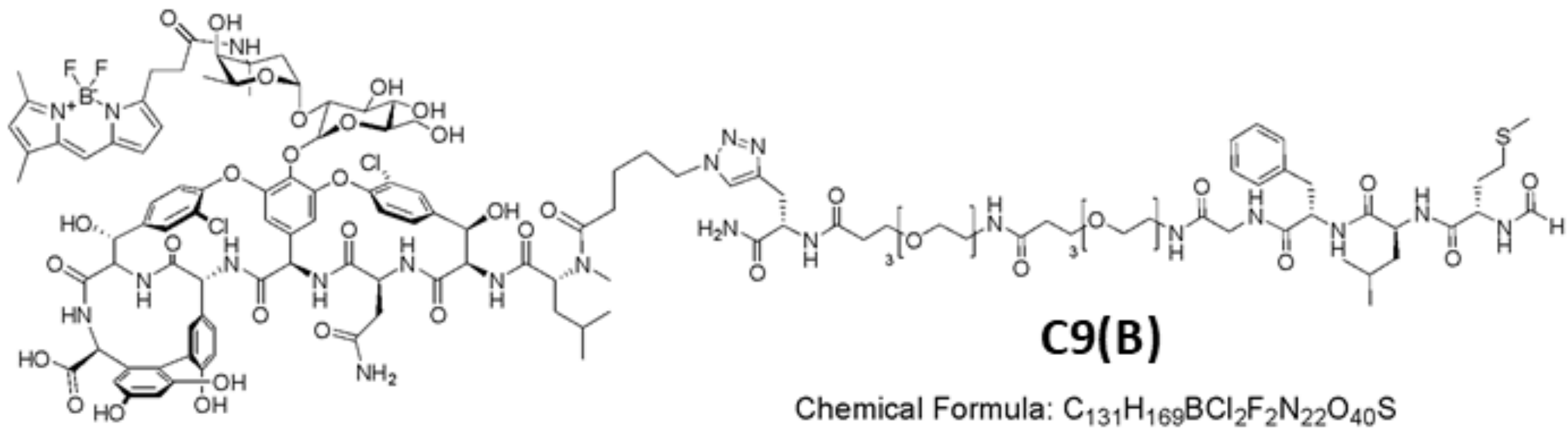

C9(B)

Chemical Formula: $C_{131}H_{169}BCl_2F_2N_{22}O_{40}S$
Exact Mass: 2841.10
Molecular Weight: 2843.67

<Chromatogram>

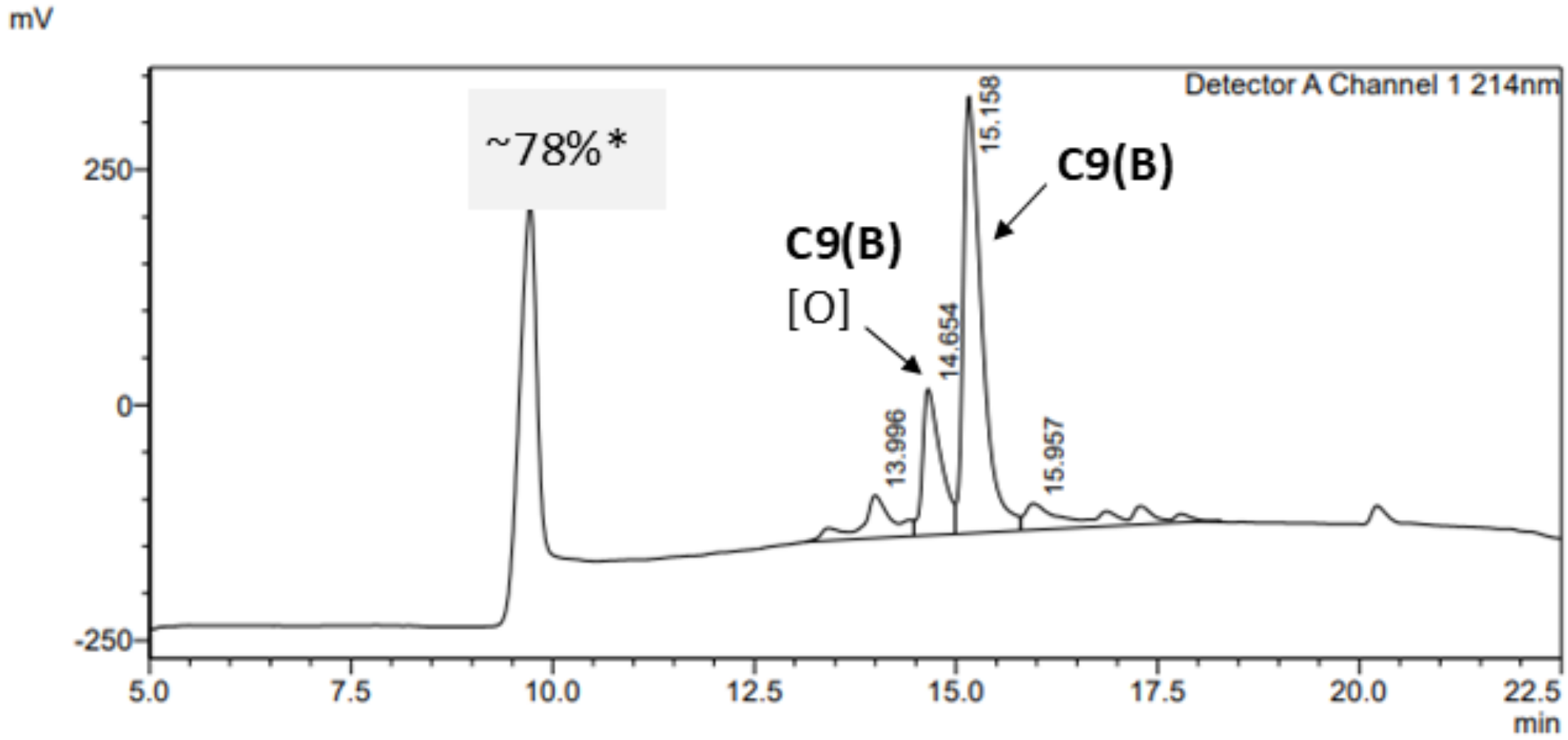


<Spectrum>

Line#:1 R.Time:15.267(Scan#:917)
MassPeaks:1950
RawMode:Single 15.267(917) BasePeak:418.2(1925928)
BG Mode:None Segment 1 - Event 1

C9(B)
Expected MW: 2843.7 Da
Found MW: 2843.6 Da

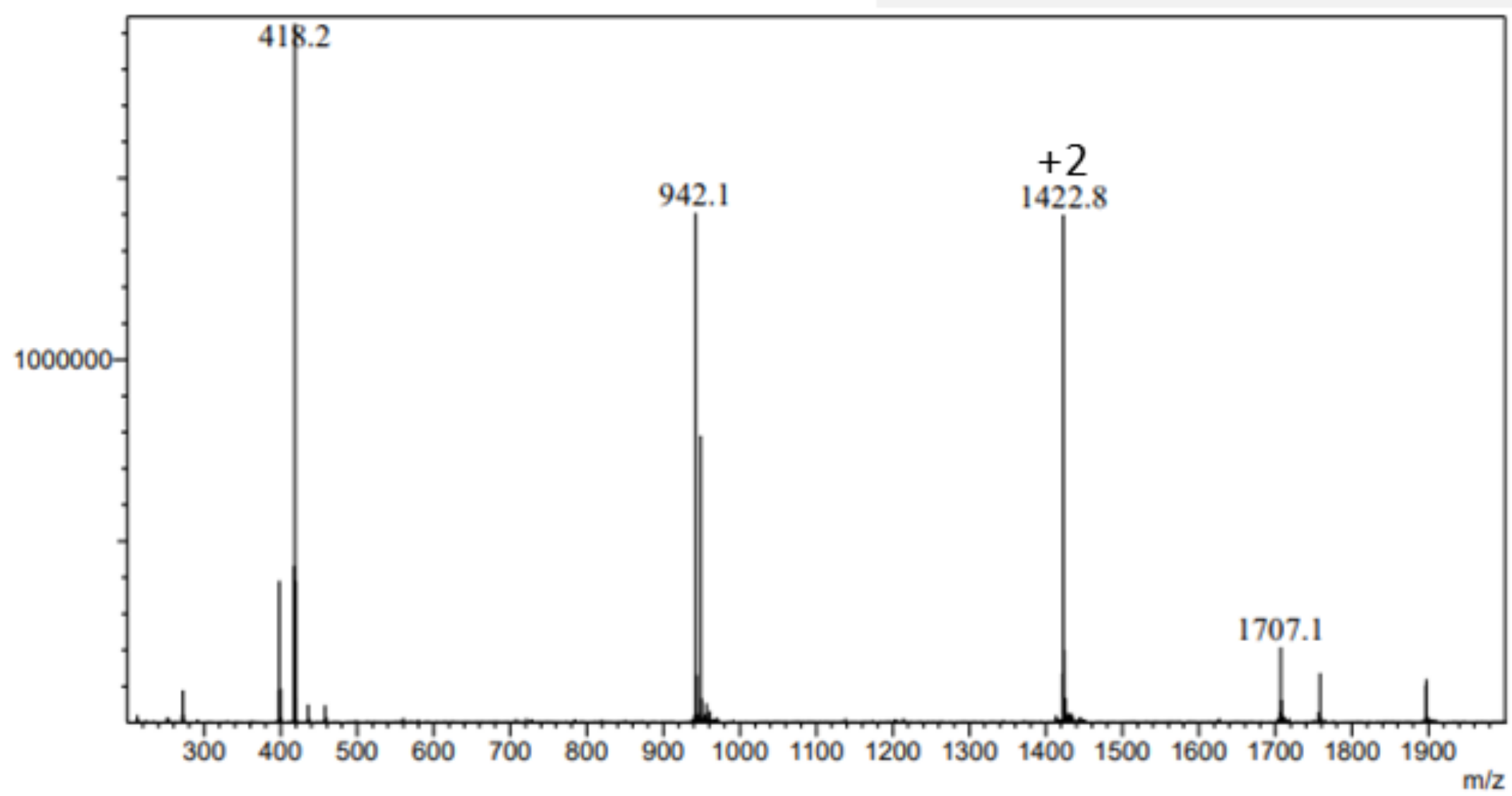


**Supplementary Figure S11.** Characterisation of vancomycin=(N-linked)-fMLFG with 6 PEG spacers. Structure, and LC and MS profiles for **C9(B)**. [O] – oxidised methionine species [M+16 Da].

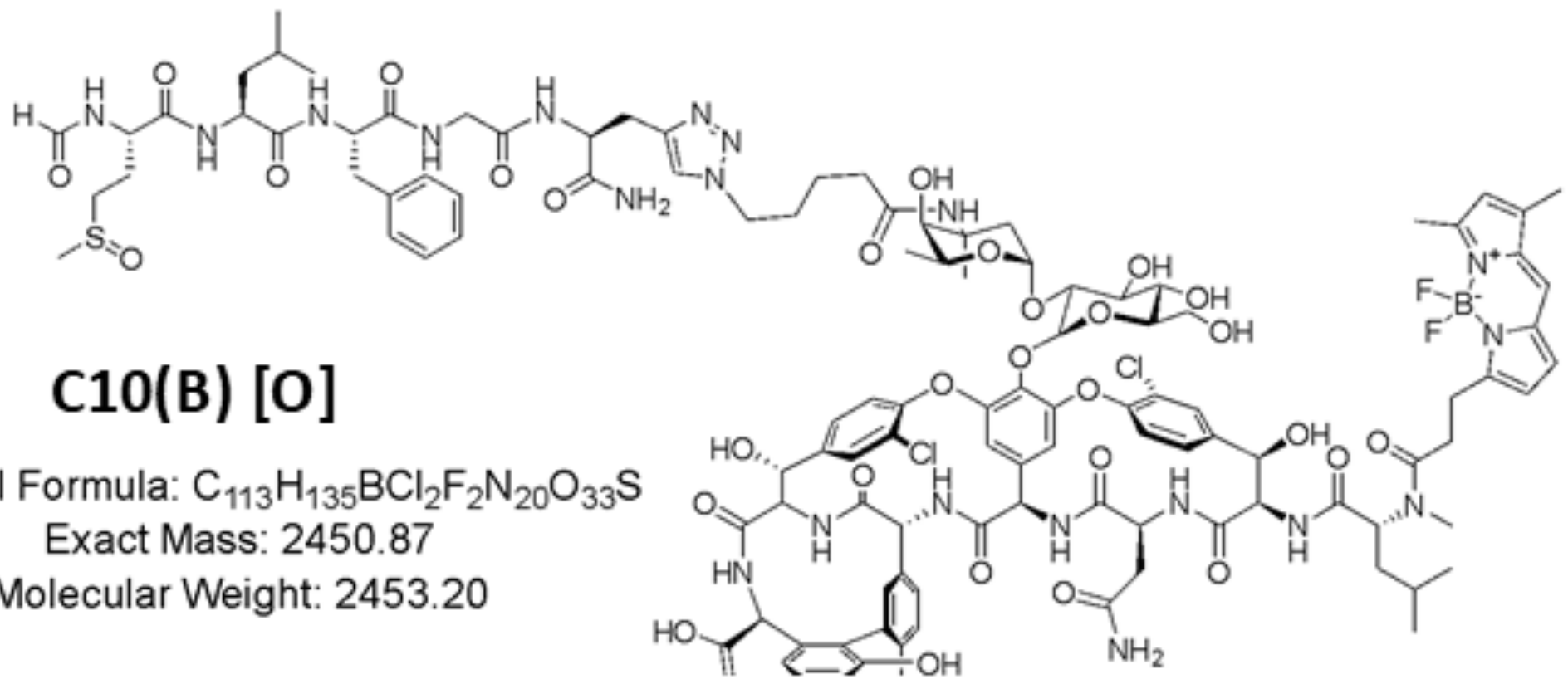


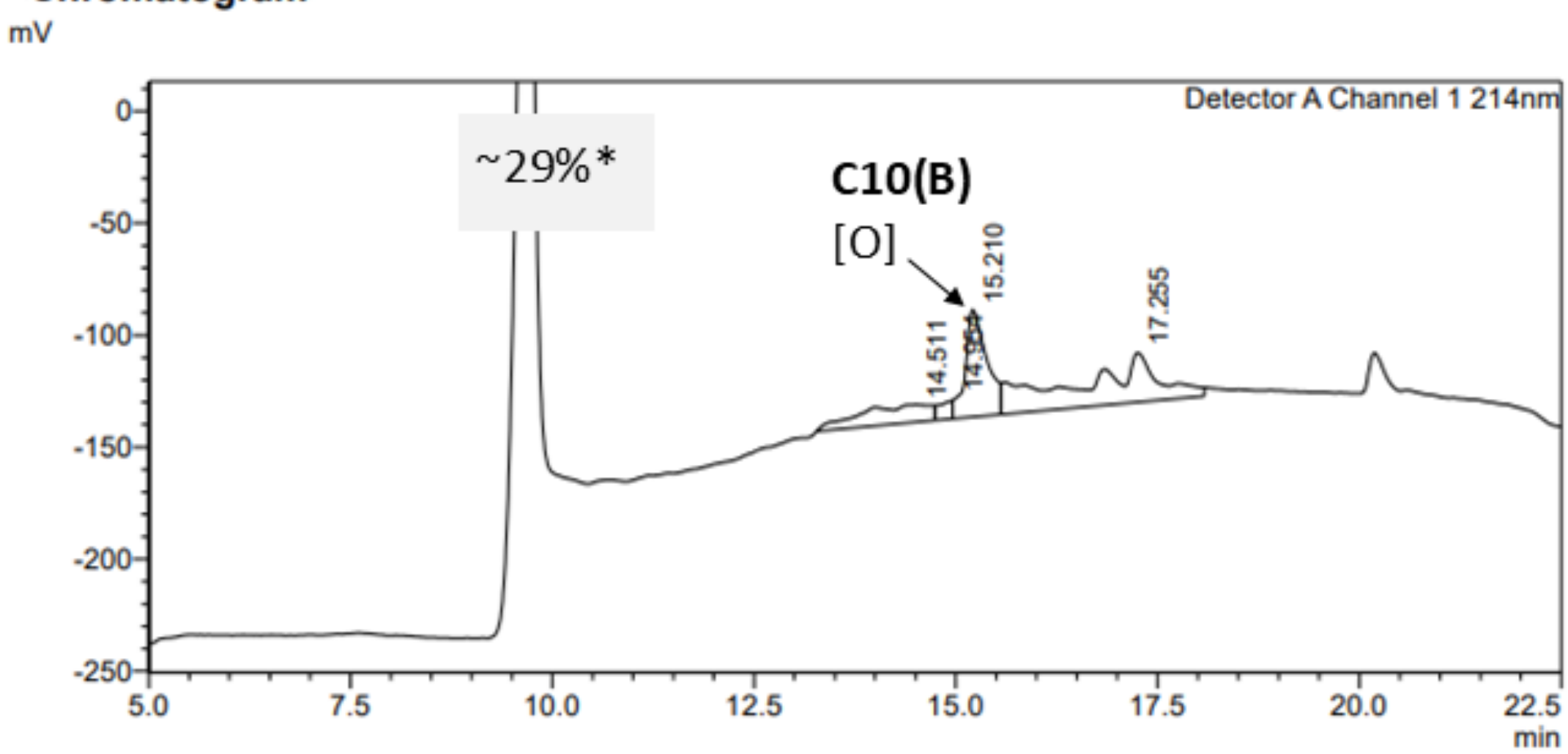


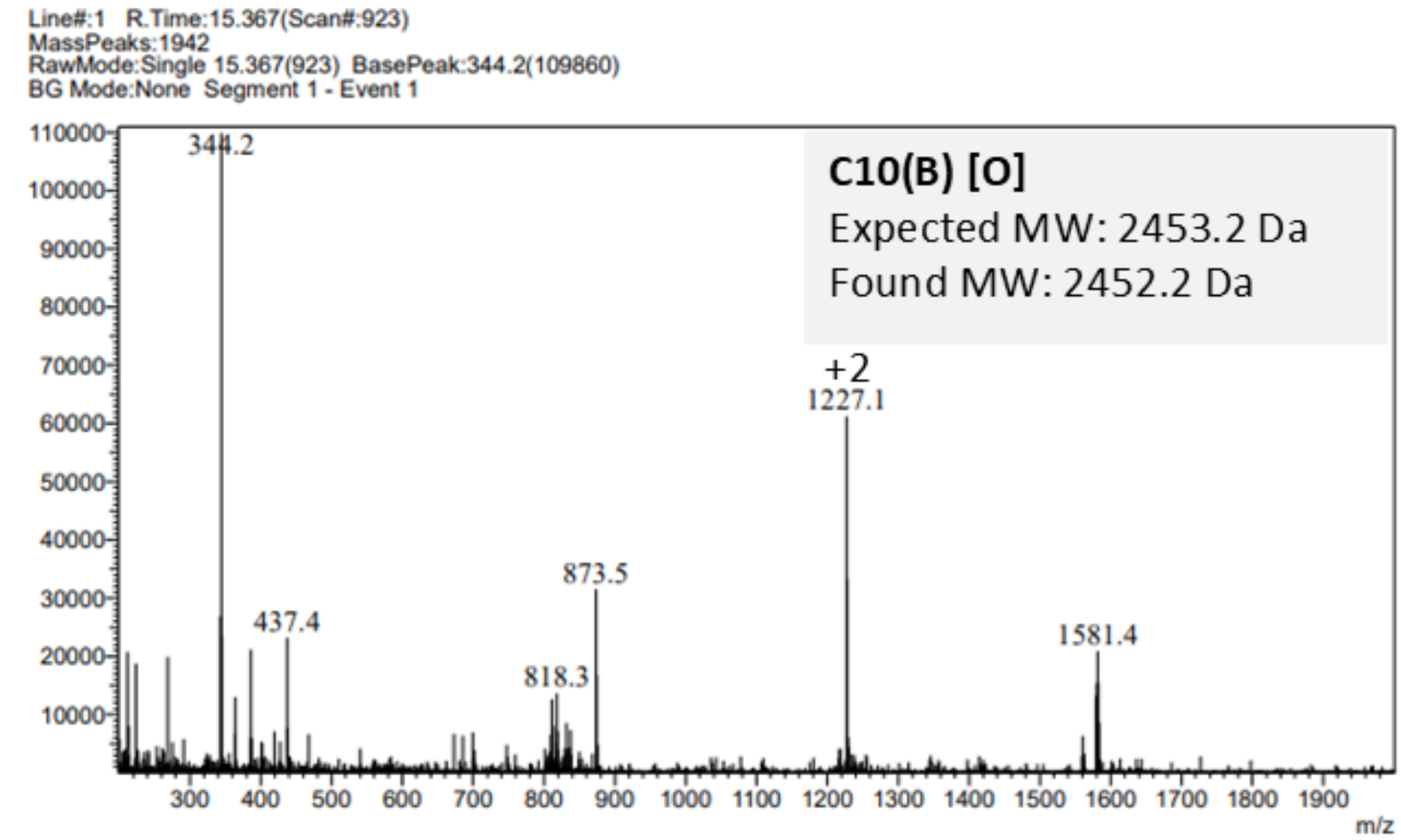


**Supplementary Figure S12.** Characterisation of vancomycin=(V-linked)-fMLFG with 0 PEG spacer. Structure, and LC and MS profiles for **C10(B)**. [O] – oxidised methionine species [M+16 Da].

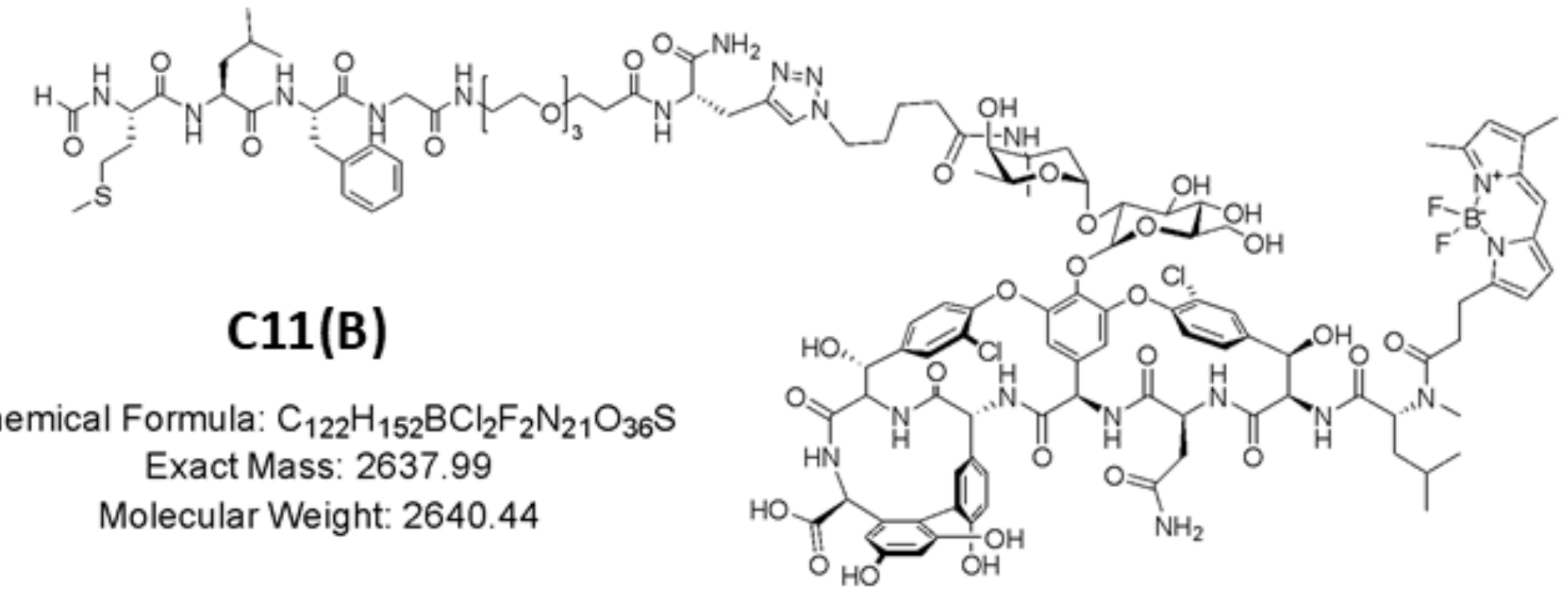

**C11(B)**

Chemical Formula: $C_{122}H_{152}BCl_2F_2N_{21}O_{36}S$
Exact Mass: 2637.99
Molecular Weight: 2640.44

**<Chromatogram>**

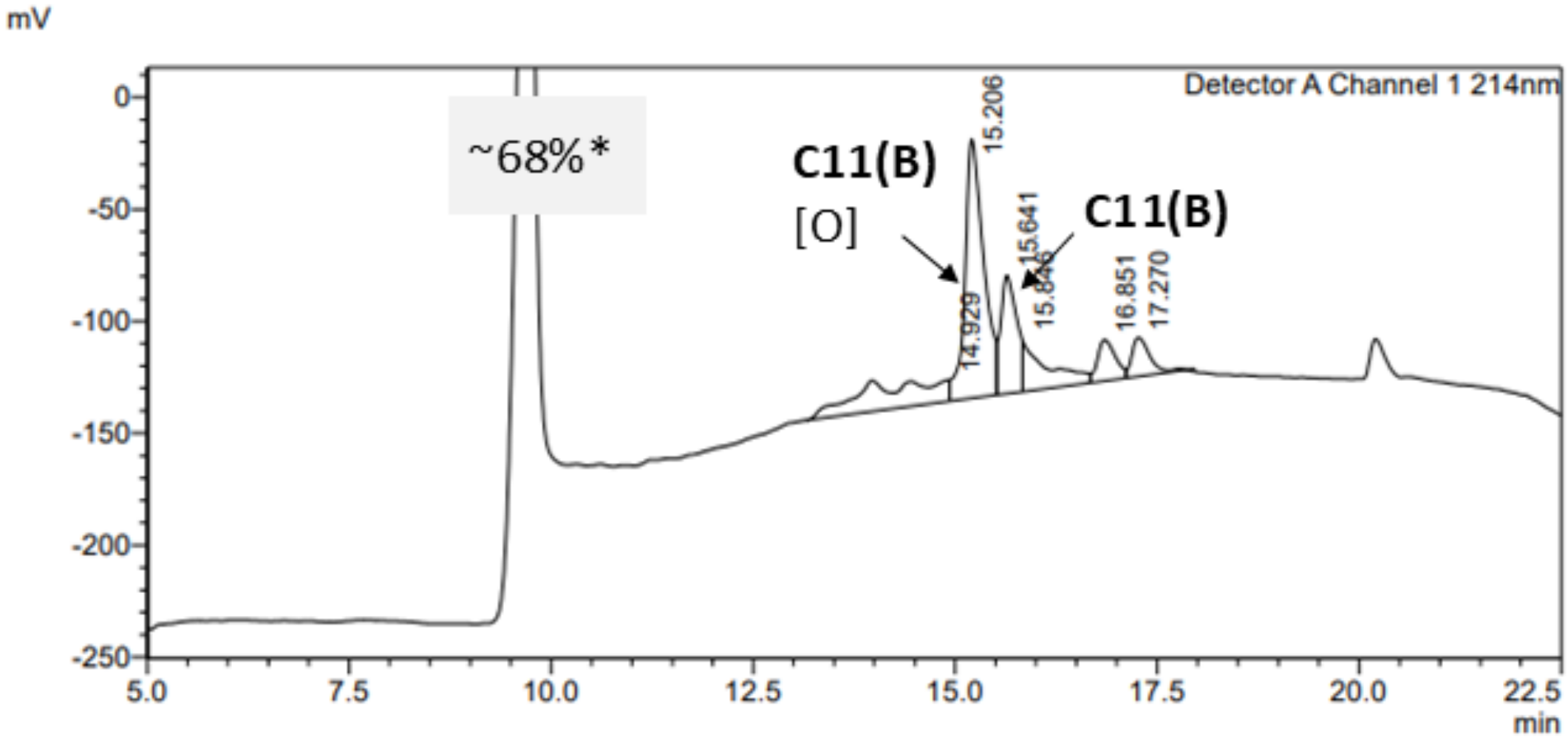


## <Spectrum>

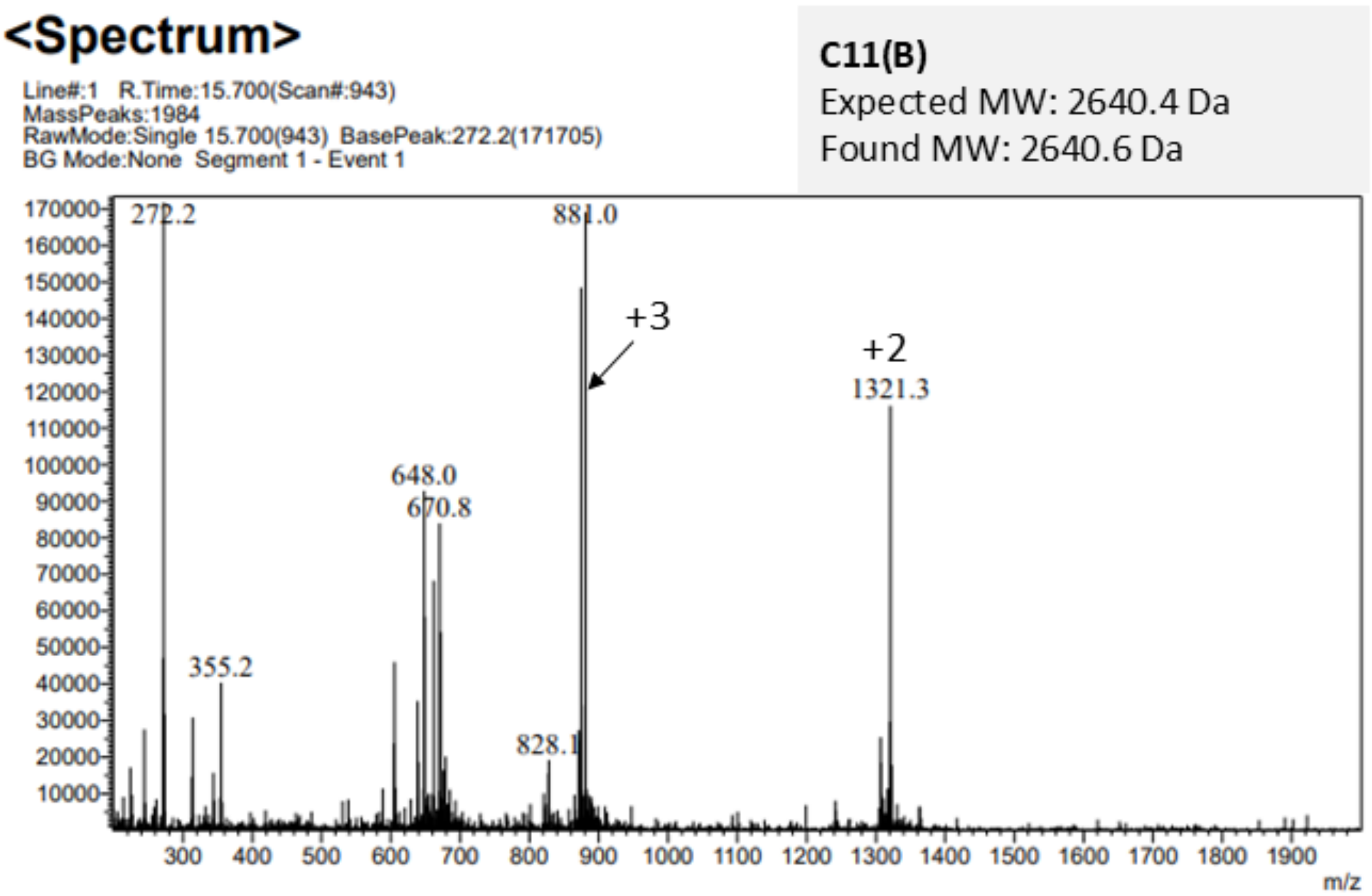


**Supplementary Figure S13.** Characterisation of vancomycin=(V-linked)-fMLFG with 3 PEG spacers. Structure, and LC and MS profiles for **C11(B)**. [O] – oxidised methionine species [M+16 Da].

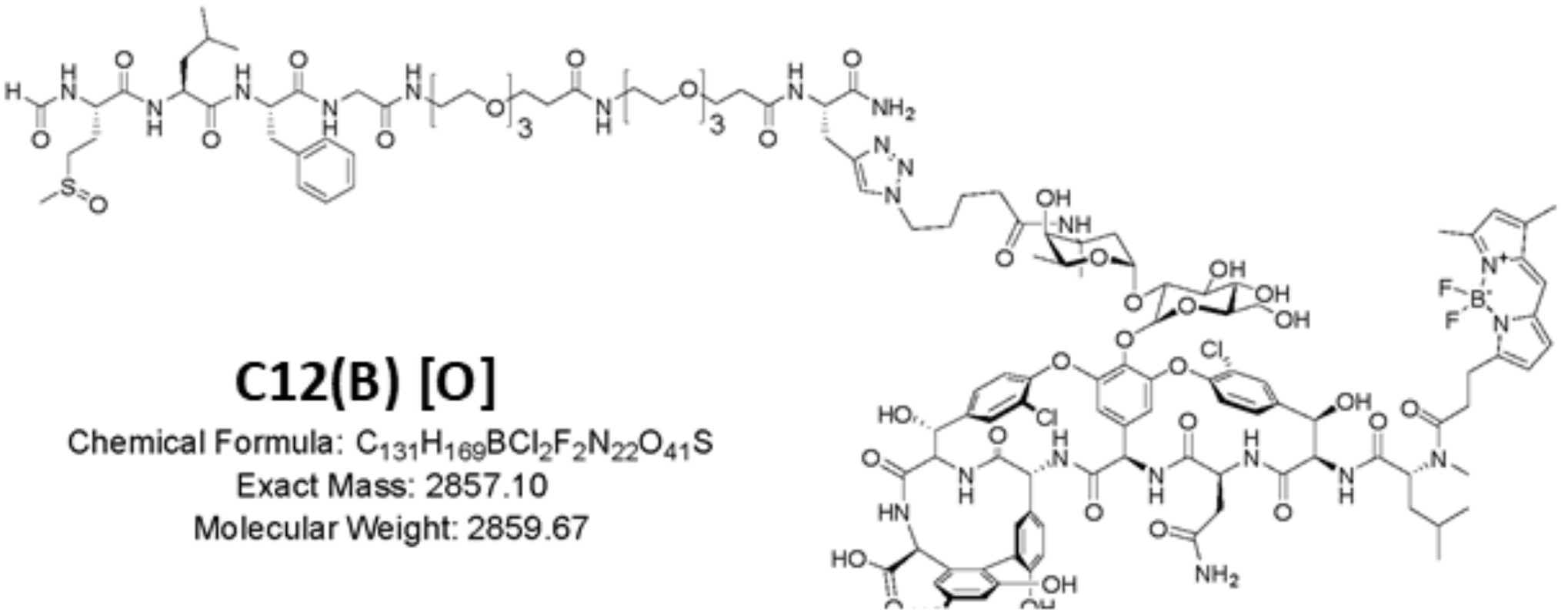


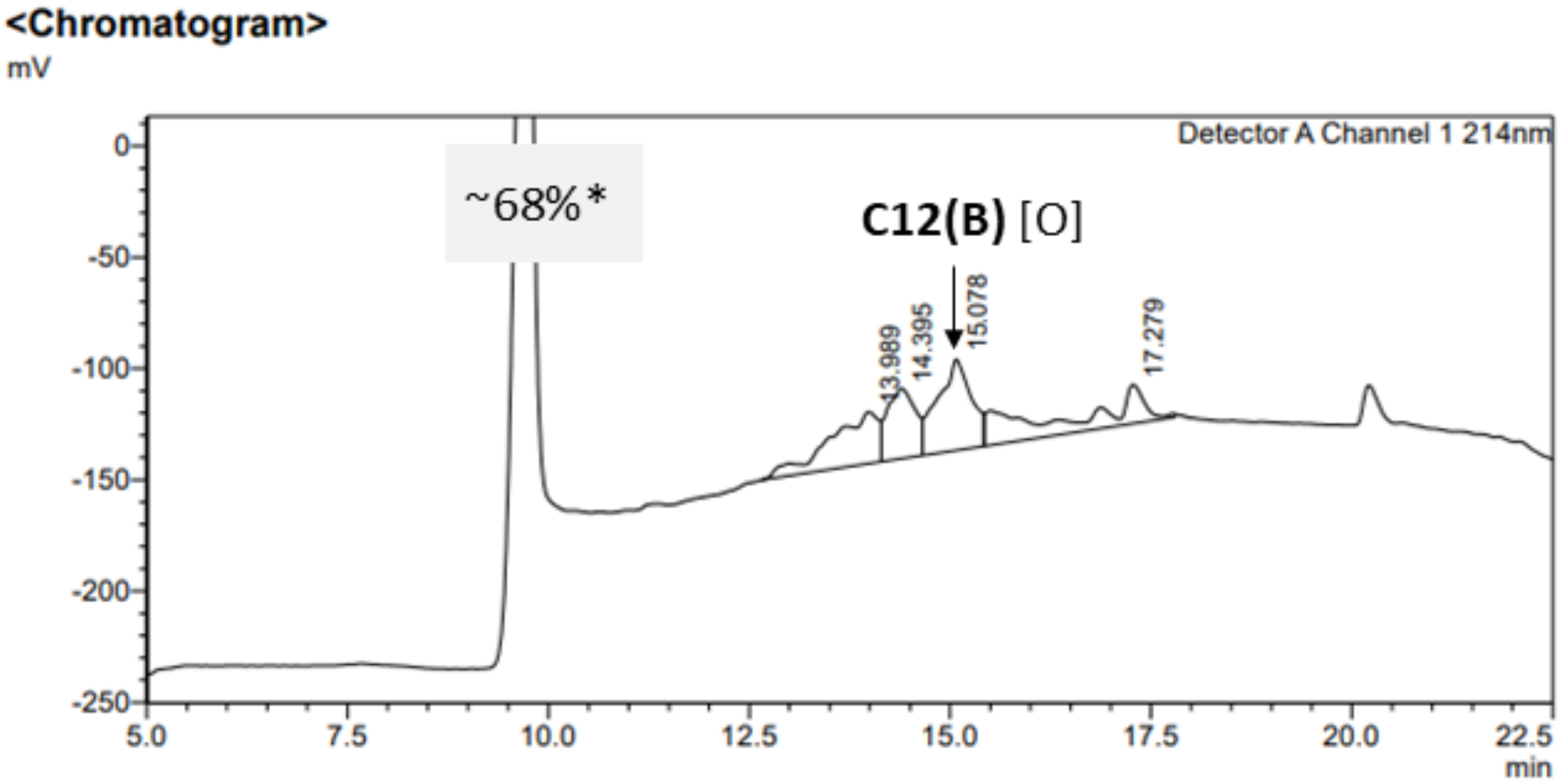


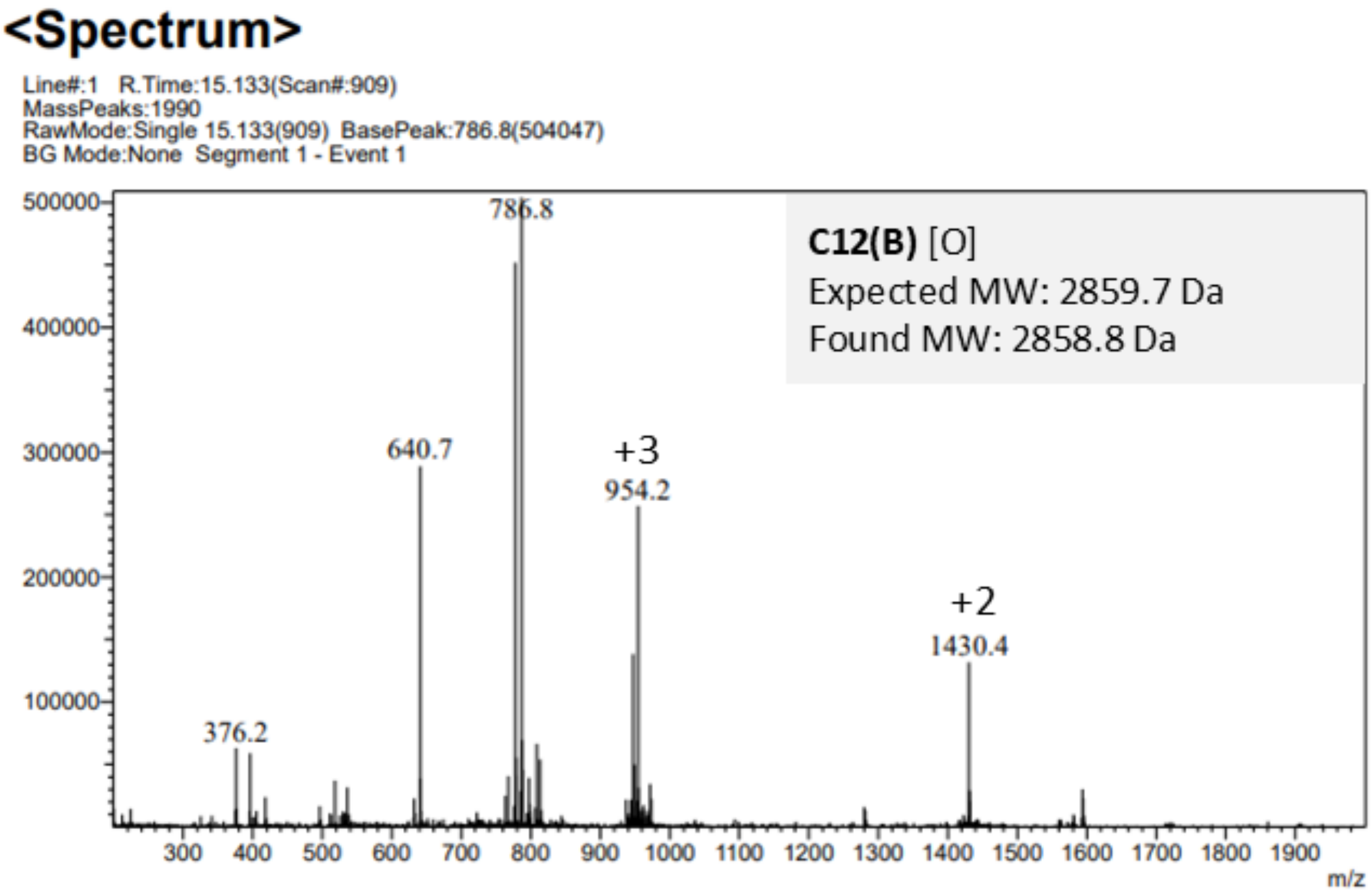


**Supplementary Figure S14.** Characterisation of vancomycin=(V-linked)-fMLFG with 6 PEG spacers. Structure, and LC and MS profiles for **C12(B)**. [O] – oxidised methionine species [M+16 Da].

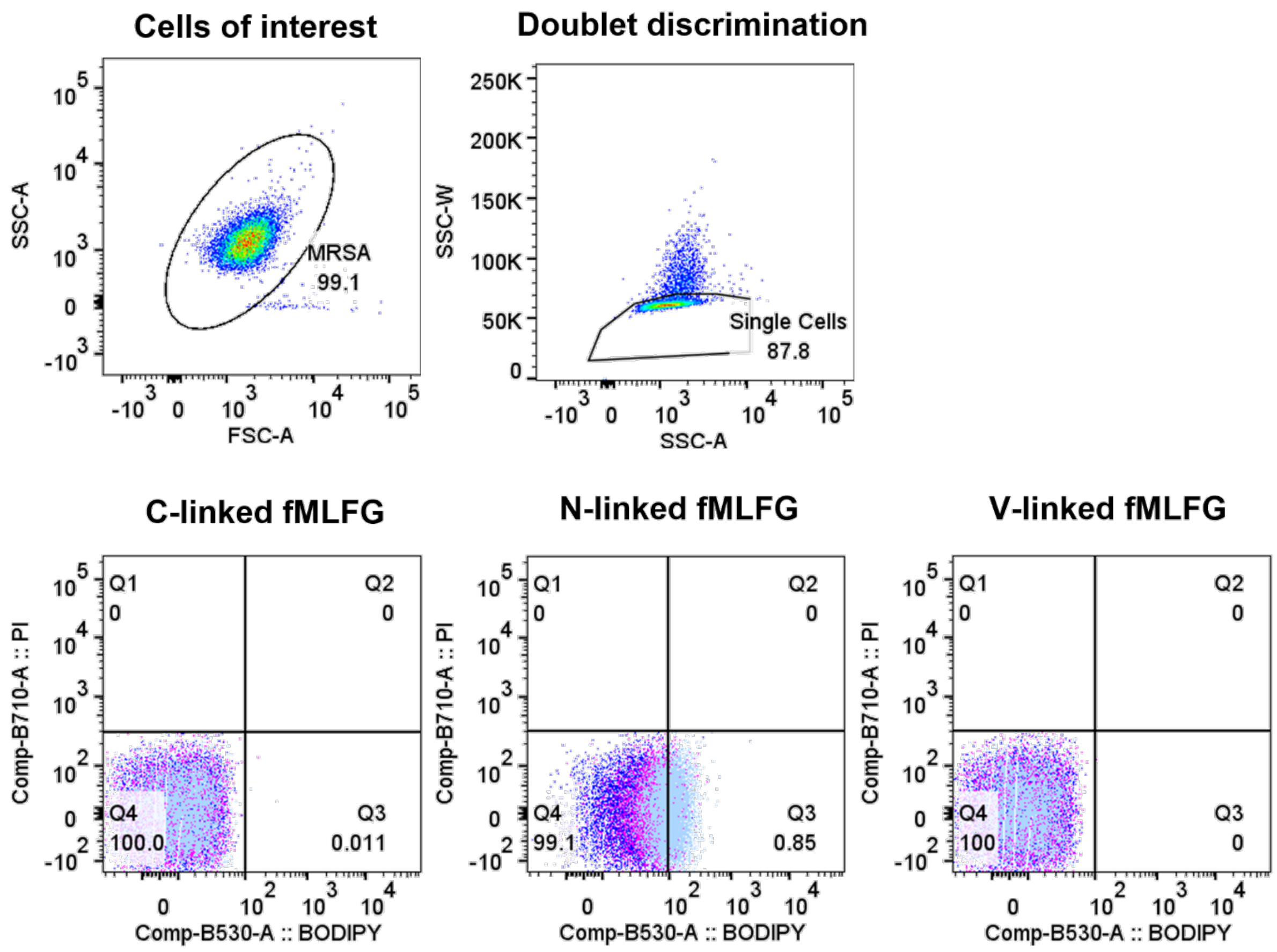


**Supplementary Figure S15.** Flow cytometry gating strategy to measure the binding of BODIPY®-labelled vancomycin and vancomycin=fMLFG conjugates in MRSA A8090. Clinical isolates of MRSA A8090 were treated with BODIPY®-labelled vancomycin=fMLFG conjugates, where fMLFG were conjugated to vancomycin at carboxyl-terminal (C-linked), amino-terminal (N-linked), or vancosamine residues (V-linked). Conjugation was also compared between different PEG linker lengths: no linker (0PEG, light blue), 3 PEG units (3PEG, purple), or 6 PEG units (6PEG, dark blue). After washing to remove excess unincorporated compounds, bacteria were resuspended and added with propidium iodide (PI) as a viability stain prior to sample acquisition on flow cytometer. Bacteria were first gated based on size (FSC-A) and granularity (SSC-A), followed by exclusion of doublets using SSC-W against SSC-A plot. In PI vs. BODIPY dot plots, viable cells would be observed in Q3 and Q4 while non-viable cells (based on positive signal from PI staining) would be observed in Q1 and Q2. Cells were further analysed for BODIPY® fluorescence where population sitting in Q2 and Q3 would indicate binding of compounds to bacteria while population sitting in Q1 and Q4 would indicate no interaction between compounds and bacteria. fMLFG conjugated at the C-terminus of vancomycin with no linkers showed the highest binding affinity to surface of MRSA. BODIPY® fluorescence was then reported as geometric mean fluorescence intensity (gMFI) value to compare the interaction of the compounds to cell surface of bacteria.

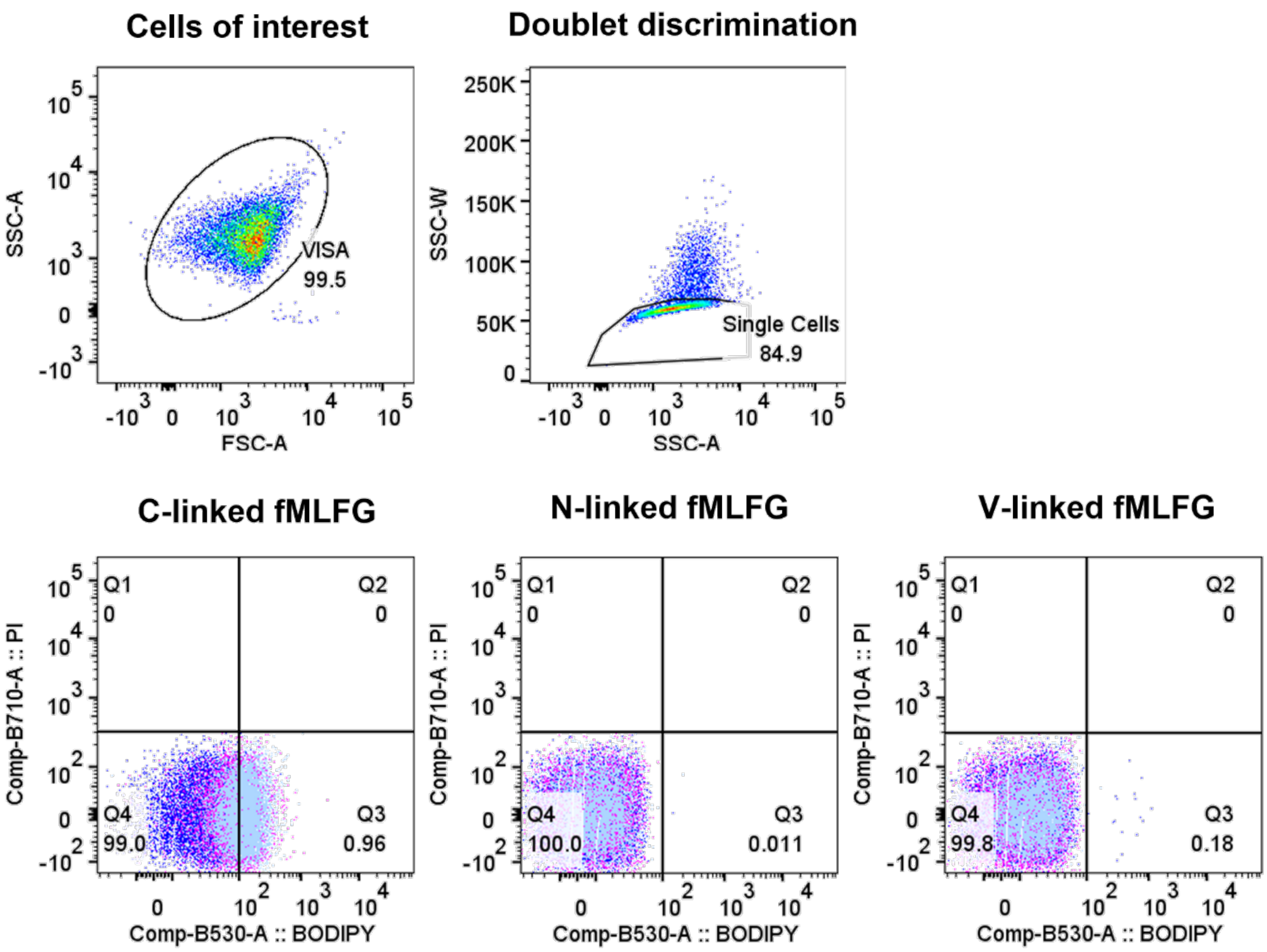


**Supplementary Figure S16.** Flow cytometry gating strategy to measure the binding of BODIPY®-labelled vancomycin and vancomycin=fMLFG conjugates in VISA A8094. Clinical isolates of VISA A8094 were treated with BODIPY®-labelled vancomycin=fMLFG conjugates, where fMLFG were conjugated to vancomycin at carboxyl-terminal (C-linked), amino-terminal (N-linked), or vancosamine residues (V-linked). Conjugation was also compared between different PEG linker lengths: no linker (0PEG, light blue), 3 PEG units (3PEG, purple), or 6 PEG units (6PEG, dark blue). After washing to remove excess unincorporated compounds, bacteria were resuspended and added with propidium iodide (PI) as a viability stain prior to sample acquisition on flow cytometer. Bacteria were first gated based on size (FSC-A) and granularity (SSC-A), followed by exclusion of doublets using SSC-W against SSC-A plot. In PI vs. BODIPY dot plots, viable cells would be observed in Q3 and Q4 while non-viable cells (based on positive signal from PI staining) would be observed in Q1 and Q2. Cells were further analysed for BODIPY® fluorescence where population sitting in Q2 and Q3 would indicate binding of compounds to bacteria while population sitting in Q1 and Q4 would indicate no interaction between compounds and bacteria. fMLFG conjugated at C-terminus of vancomycin with no linkers showed the highest binding affinity to surface of VISA. BODIPY® fluorescence was then reported as geometric mean fluorescence intensity (gMFI) value to compare the interaction of the compounds to cell surface of bacteria.

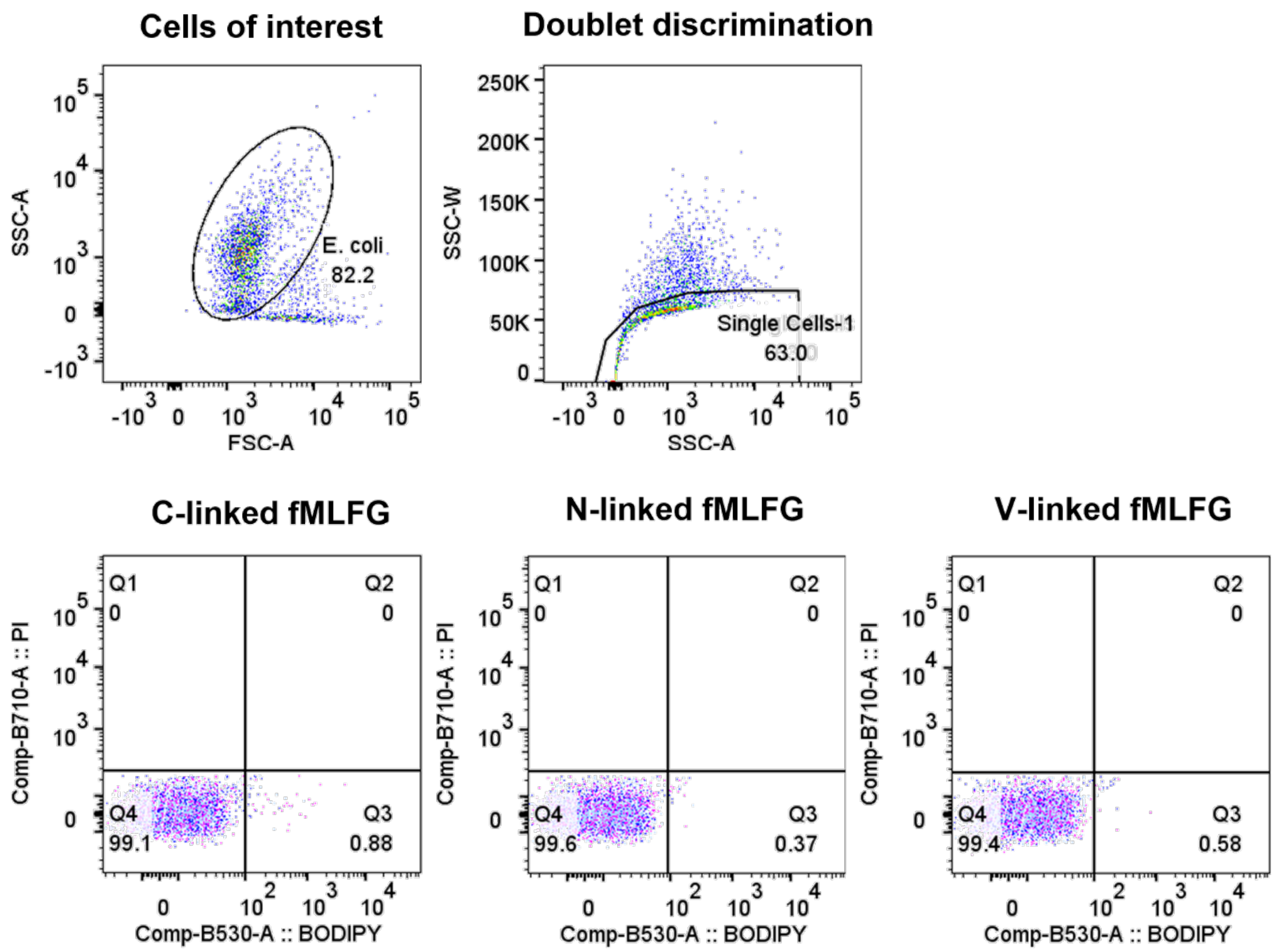


**Supplementary Figure S17.** Flow cytometry gating strategy to measure the binding of BODIPY®-labelled vancomycin and vancomycin=fMLFG conjugates in *E. coli* ATCC 25922. *E. coli* ATCC 25922 was used as a control for negative interactions between targeting element vancomycin and surface of *E. coli*. *E. coli* were treated with BODIPY®-labelled vancomycin=fMLFG conjugates, where fMLFG were conjugated to vancomycin at carboxyl-terminal (C-linked), amino-terminal (N-linked), or vancosamine residues (V-linked). Conjugation was also compared between different PEG linker lengths: no linker (0PEG, light blue), 3 PEG units (3PEG, purple), or 6 PEG units (6PEG, dark blue). After washing to remove excess unincorporated compounds, bacteria were resuspended and added with propidium iodide (PI) as a viability stain prior to sample acquisition on flow cytometer. Bacteria were first gated based on size (FSC-A) and granularity (SSC-A), followed by exclusion of doublets using SSC-W against SSC-A plot. In PI vs. BODIPY dot plots, viable cells would be observed in Q3 and Q4 while non-viable cells (based on positive signal from PI staining) would be observed in Q1 and Q2. Cells were further analysed for BODIPY® fluorescence where population sitting in Q2 and Q3 would indicate binding of compounds to bacteria while population sitting in Q1 and Q4 would indicate no interaction between compounds and bacteria. Vancomycin=fMLFG conjugates do not bind to surface of *E. coli* regardless of the fPep conjugation at different positions on vancomycin and the different PEG linker lengths. BODIPY® fluorescence was then reported as geometric mean fluorescence intensity (gMFI) value to compare the interaction of the compounds to cell surface of bacteria.

## References

[1] A. Y. Peleg, D. Monga, S. Pillai, et al., "Reduced Susceptibility to Vancomycin Influences Pathogenicity in *Staphylococcus aureus* Infection," *Journal of Infectious Diseases* **199**, (2009): 532. DOI: 10.1086/596511.
[2] G. Sakoulas, J. Alder, C. Thauvin-Eliopoulos, et al., "Induction of Daptomycin Heterogeneous Susceptibility in *Staphylococcus aureus* by Exposure to Vancomycin," *Antimicrobial Agents and Chemotherapy* **50**, (2006): 1581. DOI: 10.1128/aac.50.4.1581-1585.2006.
[3] J. A. E. Payne, J. Tailhades, F. Ellett, et al., "Antibiotic-Chemoattractants Enhance Neutrophil Clearance of Staphylococcus aureus," *Nature Communications* **12**, (2021): 1. DOI: 10.1038/s41467-021-26244-5.
